# A genetically regulated SKAP2 switch modulates human T lymphocyte adhesion and migration

**DOI:** 10.1084/jem.20260507

**Published:** 2026-10-20

**Authors:** Mara Cetty Spinella, Francesco Gualdrini, Cristina Leoni, Jun Siong Low, Sara Polletti, Amy Kenyon, Samuele Mauri, Veronica Vallelonga, Mehrpouya Mostanfar, Matteo Pecoraro, Chiara Soriani, Roberta Marzi, Elena Grassi, Fabio Bedin, Elena Foli, Niccolò Bianchi, Emina Džafo, Sandra Jovic, Alessandro Cuomo, Simona Rodighiero, David Jarrossay, Federica Sallusto, Gioacchino Natoli, Silvia Monticelli

**Affiliations:** 1 Institute for Research in Biomedicine, Università della Svizzera italiana, Bellinzona, Switzerland; 2 Department of Experimental Oncology, IEO, European Institute of Oncology IRCCS, Milan, Italy; 3 Institute of Microbiology, ETH Zürich, Zurich, Switzerland; 4 INOC – Istituto Nazionale Oncologico Candiolo, IRCCS, Candiolo, Italy; 5 Department of Oncology, University of Turin, Turin, Italy

## Abstract

Genome-wide association studies investigating autoimmune disorders may reveal previously unrecognized regulators of immune cell functions. A Crohn’s disease risk allele was associated with increased expression in T lymphocytes of the adapter protein Src kinase–associated phosphoprotein 2 (SKAP2), whose functional role in these cells is unknown. By analyzing T cells from individuals homozygous for either allelic variant or engineered to modulate SKAP2 endogenous levels, we found that SKAP2 promotes T cell adhesion while restraining migration, acting nonredundantly with its homologue SKAP1. Consistent with such functional differences, SKAP1 and SKAP2 displayed distinct activation-induced regulation as well as unique protein interactomes. Prolonged TCR activation triggered caspase-mediated SKAP2 cleavage, leading to the disassembly of its interactome and enabling a migratory program. Together, these findings identify SKAP2 as a genetically regulated modulator of T cell function whose expression and activity control the balance between adhesion and migration.

## Teaser

Spinella et al. linked a Crohn’s disease risk allele regulating SKAP2 expression to T lymphocyte function. SKAP2 enhances cell adhesion while suppressing migration. Sustained TCR signaling triggers its proteolytic cleavage to enable migration. Overall, genetic alteration of SKAP2 levels drives T cell dysfunction and promotes autoimmune disease.

## Graphical Abstract

**Figure:**
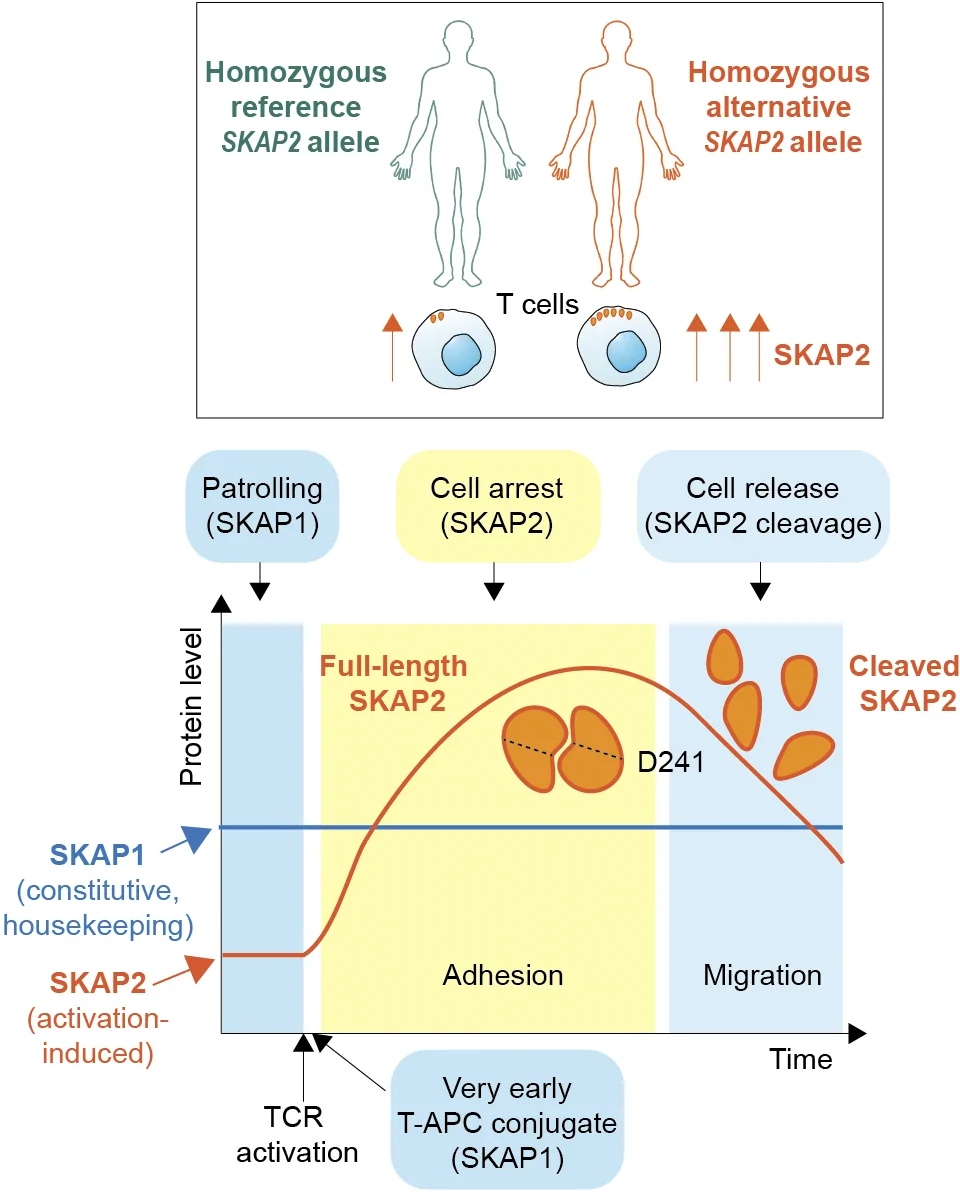

## Introduction

Beyond their clinical relevance, signals identified in genome-wide association studies (GWAS) may in principle unveil the roles of specific genes in normal biological processes. Despite the abundance of genetic risk loci identified for many immunological disorders and the deployment of fine-mapping approaches to identify the causal variant(s), only a minor fraction of risk loci has been linked to a causal gene, a specific immune cell type affected by the genetic variation, and ultimately to a mechanistically defined process. This notion is well exemplified by inflammatory bowel disease (IBD), which encompasses Crohn’s disease (CD) and ulcerative colitis. IBD is a multifactorial disease associated with increased colorectal cancer risk (Jess et al., 2012; Lutgens et al., 2008) and resulting from the interaction between genetic predisposition and local or environmental factors, with a critical role of T lymphocytes in disease pathogenesis (Baumgart and Sandborn, 2012; Maloy and Powrie, 2011). More than ∼200 IBD risk loci have been identified in GWAS cohorts (Huang et al., 2017; Jostins et al., 2012; Liu et al., 2015; Momozawa et al., 2018; Sazonovs et al., 2022), and many of them may point toward previously unrecognized regulators of T cell function. However, owing to the inherent challenges in deconvoluting GWAS signals, the majority of these loci remain orphan in terms of assignment to genes, immune cell types, or biological processes.

Among the identified IBD risk loci, we were drawn to a 0.6-Mbp genomic region that has been linked to an increased risk of CD (Jostins et al., 2012; Liu et al., 2015; Momozawa et al., 2018; Schmiedel et al., 2018) as well as of other T cell–mediated autoimmune diseases such as type I diabetes and multiple sclerosis (Barrett et al., 2009; Beecham et al., 2013). Notably, this region contains a single gene, *SKAP2* (Src kinase–associated phosphoprotein 2). SKAP2 is an adapter protein that shows widespread expression in immune cells and that was initially identified as a homolog of the lymphoid tissue-enriched protein SKAP1 (Liu et al., 1998; Marie-Cardine et al., 1998). However, in addition to different expression profiles, the two proteins share only 42.7% amino acid identity, arguing for functional specialization rather than redundancy.

Both SKAP1 and SKAP2 have been involved in signal-dependent regulation of integrin-mediated adhesion and migration. Integrins are heterodimeric transmembrane proteins that enable adhesive interactions of T lymphocytes with endothelia and other immune cells, as well as extracellular matrix components (Springer, 1990). The critical role of adhesion-mediated mechanisms in T lymphocyte recruitment, migration, and activation is evident in both immune deficiencies and therapeutic interventions. In leukocyte adhesion deficiency (LAD), a group of heterogeneous immunodeficiency syndromes caused by mutations in integrins or associated proteins, defects in cell adhesion impair immune function (Hanna and Etzioni, 2012; Kishimoto et al., 1987). Conversely, integrin blockade has shown therapeutic effects in immune disease models (Gerber et al., 2013; Zundler et al., 2017) and human autoimmune disorders (Shimaoka and Springer, 2003; Slack et al., 2022). These observations highlight the necessity of precise regulation of these adhesion-dependent processes for normal immune system function.

In the context of integrin regulation, SKAP1 was shown to positively, yet only transiently, impact clustering of lymphocyte function–associate antigen-1 (LFA-1), a heterodimer of the integrins ITGAL and ITGB2, during the very early phase of T cell activation (Jo et al., 2005; Wang et al., 2007; Wang et al., 2003), while being dispensable for chemokine-induced migration in the absence of TCR signaling (Wang et al., 2010). SKAP2 was instead shown to be required for integrin-induced cytoskeletal rearrangement in macrophages (Alenghat et al., 2012) and for integrin-mediated recruitment of neutrophils to inflamed tissues in vivo, to the point that loss of Skap2 was sufficient to induce a LAD-like phenotype in mice (Boras et al., 2017). *Skap2* deletion also enhanced motility of dendritic cells, which was attributed to the loss of their ability to adhere to the extracellular matrix (Reinhold et al., 2009), thus suggesting an important role for SKAP2 in regulating immune cell adhesion. These initial data point toward an apparent dichotomy in the roles of these two proteins, with SKAP1 regulating T lymphocyte function and SKAP2 controlling adhesion and motility in myeloid cells. However, *Skap2*-deficient mice exhibited decreased severity of experimental autoimmune encephalomyelitis, a canonical T cell–mediated disease model (Togni et al., 2005), suggesting its possible involvement also in the regulation of T cells. Nevertheless, the role of SKAP2 in T cell activation as well as its interplay with SKAP1 remain unknown.

Given the association of the *SKAP2* locus with autoimmune diseases and the paucity of knowledge regarding the function of SKAP2 in T cells, we set out to investigate the regulation and impact of SKAP2 expression in human T lymphocytes. First, we found that the allelic variant associated with CD risk correlated with elevated SKAP2 protein expression in human T cells. Next, by generating T cells modified either to increase endogenous *SKAP2* gene expression via CRISPR-mediated transcriptional activation (CRISPRa) or to abrogate SKAP2 expression through CRISPR-Cas9 genome editing, we found that SKAP2 plays a nonredundant role in regulating T cell adhesion and functions in response to TCR triggering. While SKAP1 was required for optimal T cell migration in response to chemokine signals, SKAP2 was uniquely responsible for modulating cell adhesion and even opposed migration. Analysis of the SKAP1 and SKAP2 protein interactomes in T cells revealed that, on the one hand, SKAP2 is part of a complex also containing SKAP1, but on the other, it also forms “private” interactions that likely account for the functional divergence of the two proteins. In addition, we found that T cell activation led to the upregulation of SKAP2, but not SKAP1, expression, followed by its caspase-mediated cleavage at later stages of activation. Such cleavage led to the release of the SKAP2 interactome, relieving T cell adhesive capacity and ultimately enabling migration.

Overall, building on GWAS data, we uncovered a nonredundant, SKAP2-dependent mechanism of regulation of activation-induced T cell adhesion.

## Results

### A single-gene CD risk locus containing *SKAP2*

**Figure 1.**
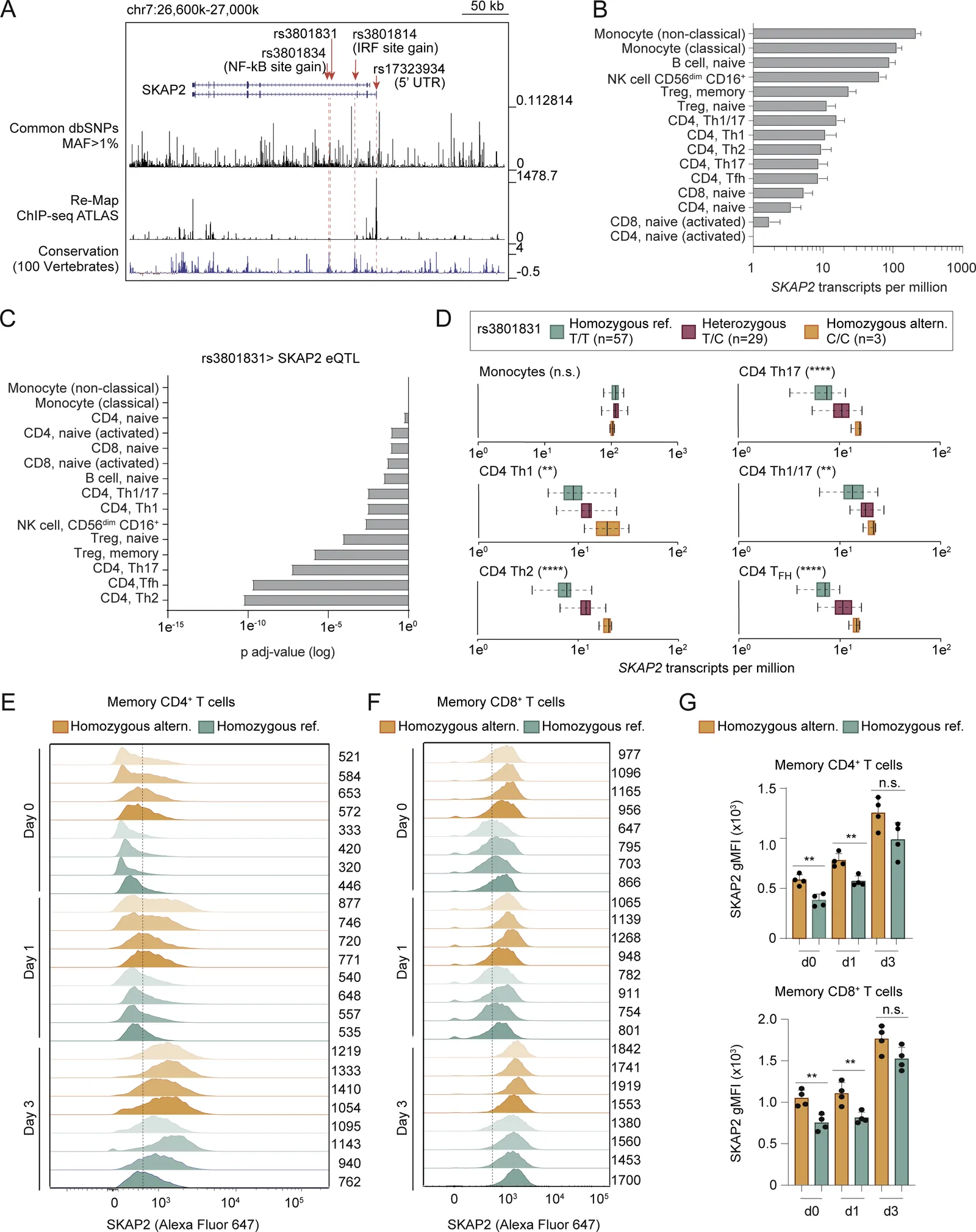
**Genetic variation in the *SKAP2* locus determines the levels of SKAP2 expression in human T lymphocytes. (A)** Snapshot of the *SKAP2* locus showing, from top to bottom: all the SNPs present in the population with a frequency >1% (y-axis values represent the density of SNPs per base pair); the candidate regulatory regions in the locus based on the integrative ReMap ChIP-seq atlas (y-axis values represent the density of overlapping ChIP-seq peaks); and the local DNA sequence conservation in 100 vertebrates. Selected SNPs in candidate regulatory regions, in which the minor-frequency variant is associated with transcription factor–binding site changes or altered 5′UTR, are also shown (red arrows). The location of *rs3801831*, one of the SNPs with the highest ncER score, is also indicated. **(B)** Expression of *SKAP2* in multiple immune cell types. Data were obtained from the DICE database (Schmiedel et al., 2018). **(C)** Bar chart showing the adj. P values (−log_10_ scale) for the association between SNP *rs3801831* and *SKAP2* gene expression across immune cell types from the DICE database. Statistical testing and multiple testing correction follow the eQTL evaluation methodology as reported (Schmiedel et al., 2018). This SNP was selected as a representative example among the 194 variants in linkage disequilibrium since it was one of those with the highest ncER score. **(D)***SKAP2* mRNA expression levels in different immune cell types in subjects heterozygous or homozygous for the major (reference) or minor (alternative) variant of one representative SNP. Data were extracted from the DICE database (Schmiedel et al., 2018). Monocytes: n.s. adj. P = 1.0, CD4 Th17 ****adj. P = 5.1 × 10^−8^, CD4 Th1 **adj. P = 2.8 × 10^−3^, CD4 Th1/17 **adj. P = 2.8 × 10^−3^, CD4 Th2 ****adj. P = 5.3 × 10^−11^, CD4 T_FH_ ****adj. P = 1.8 × 10^−10^. **(E–G)** Intracellular staining for SKAP2 expression in memory T cells. Cells were separated from the peripheral blood of healthy donors homozygous for the reference or the alternative *SKAP2* allele. Intracellular staining was performed ex vivo at day 0 and at different time points following stimulation with aCD3/aCD28. **(E)** Memory CD4^+ ^T cells. **(F)** CD8^+^ T cells. The geometric mean fluorescence intensity (gMFI) is shown on the right. In each line, different color intensities represent different donors (alternative allele: shades of orange; reference allele: shades of green). **(G)** Compiled gMFI results of the donors shown in E and F. Mean ± SD, unpaired *t* test, two-tailed. Each dot represents one donor. *n* = 4 donor, homozygous alternative and *n* = 4 donors, homozygous reference. Memory CD4^+^ T cells from left to right: **P = 0.0027, **P = 0.0030, and n.s. P = 0.0590. Memory CD8^+^ T cells from left to right: **P = 0.0053, **P = 0.0081, and n.s. P = 0.0626.

We focused our attention on a 0.6-Mbp CD risk locus encompassing a CD-associated credible set of 194 noncoding genetic variants in linkage disequilibrium (Fig. 1 A). This locus has previously been linked to an increased risk of developing CD (Liu et al., 2015; Momozawa et al., 2018; Schmiedel et al., 2018), type I diabetes mellitus (Barrett et al., 2009), and multiple sclerosis (Beecham et al., 2013). No single causal variant could be linked to CD, even by fine-mapping analysis (Huang et al., 2017). However, the sum of all the 194 variants reached a posterior probability higher than 95% of being causal, suggesting the likely presence of the disease-causal variant(s). Moreover, since this region contains a single gene, *SKAP2*, this is likely to be the causative one. To gain insights into the regulatory impact of these 194 noncoding variants, we employed a genome essentiality metric, the noncoding essential regulation (ncER) score (Wells et al., 2019). This score estimates the likelihood of each noncoding variant being deleterious by integrating 38 features related to parameters such as evolutionary conservation, functional annotations, as well as regulatory element predictions. The score is provided as percentiles, with a value higher than 90 suggesting likely essentiality. We found that 32 of the 194 single-nucleotide polymorphisms (SNPs) had an ncER score >90 (Data S1), a finding also consistent with their association with regions conserved in evolution and predicted to have regulatory functions based on ReMap, an integrative analysis of hundreds of publicly available chromatin immunoprecipitation sequencing (ChIP-seq) datasets (Griffon et al., 2015) (Fig. 1 A). Hence, many of the SNPs in the *SKAP2* locus are predicted to have regulatory consequences on gene expression. For instance, the gain of canonical motifs for the transcription factors NF-kB and IRF was selectively linked to the risk-associated allelic variant (Fig. 1 A).

Next, we interrogated the EMBL-EBI expression quantitative trait locus (eQTL) catalogue and the DICE database, which report eQTLs in the immune cells of healthy individuals (Schmiedel et al., 2018). All datasets that included T cells revealed that eQTLs are present in the *SKAP2* locus in these cells (Chen et al., 2016; Gutierrez-Arcelus et al., 2013; Schmiedel et al., 2018). Similarly, a few recent studies also showed the presence of *SKAP2* eQTLs in T cells (Schmiedel et al., 2022; Soskic et al., 2022). Although *SKAP2* is more highly expressed in the myeloid compartment and in B lymphocytes compared with T cells (Fig. 1 B), the minor allelic variant (which has a prevalence of ∼17% in the European population, resulting in a frequency of ∼3% of homozygous individuals) (Schmiedel et al., 2018) is associated with an eQTL selective for several subsets of CD4^+^ T lymphocytes, where its presence leads to elevated *SKAP2* mRNA expression (Schmiedel et al., 2018), with no detectable effects in myeloid cells (Fig. 1, C and D).

**Figure S1.**
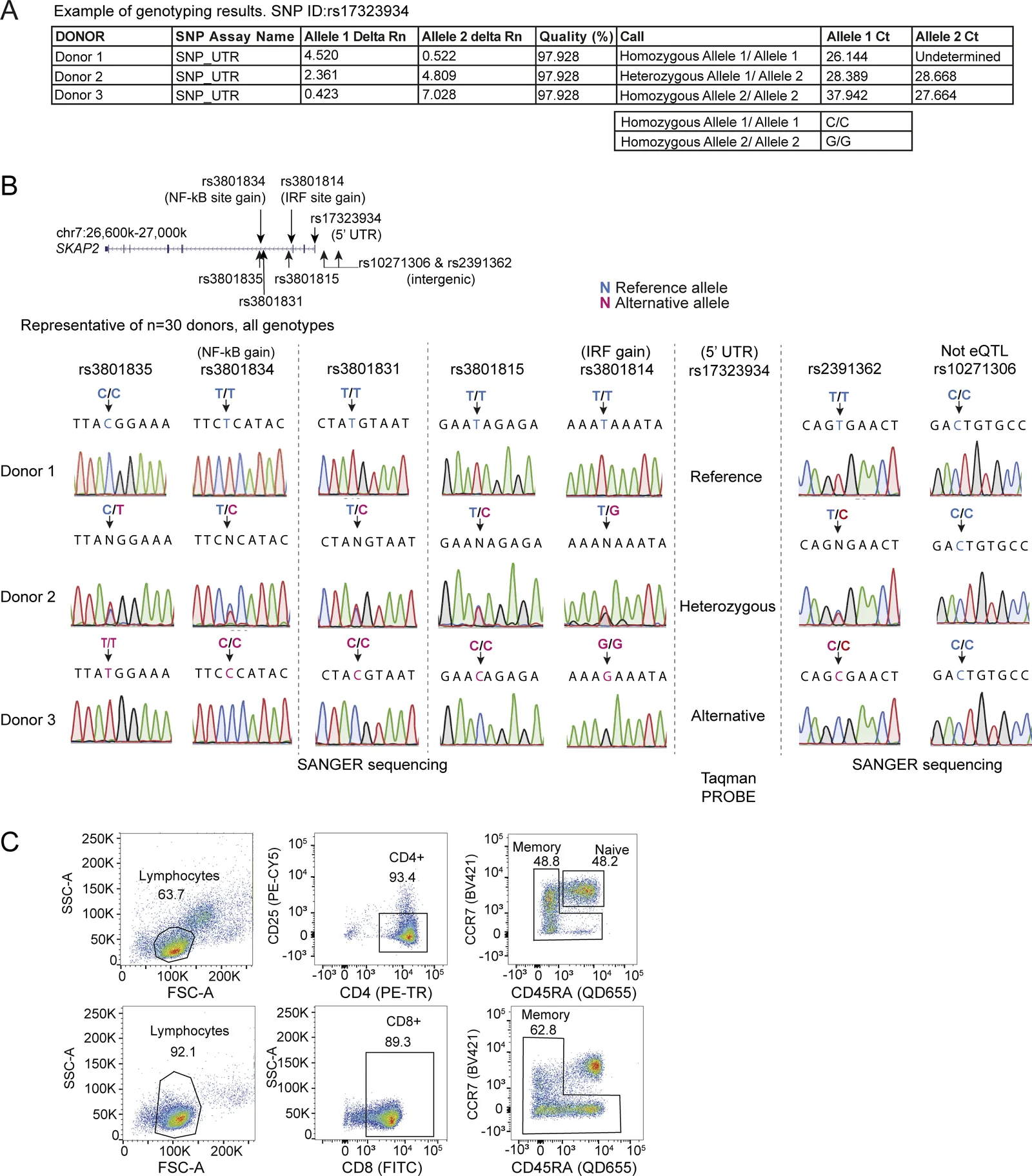
**Genetic variation in the *SKAP2* locus. (A)** TaqMan genotyping results for *rs17323934*. The “Quality” value indicates the confidence of the genotype call. Representative of *n* > 20 independent experiments. **(B)** Sanger sequencing of gDNA regions surrounding the indicated SNPs (schematic representation on top) in the *SKAP2* locus. PCR primers were designed to amplify the selected regions, followed by sequencing. The SNP *rs3801835* is not among the SNPs with the highest ncER score (Data S1), but it was sequenced because in proximity to *rs3801834*. Similarly, *rs2391362* is not among the SNPs with the highest ncER score, but it was sequenced because in proximity to *rs10271306*. The latter was used as a non-eQTL control (not on the list in Data S1). One representative donor of *n* = 30 (all genotypes) is shown. **(C)** Gating strategy for the enrichment of human CD4^+^ memory and naïve T cell subsets (upper panels) and memory CD8^+^ T lymphocytes (lower panels) from human PBMCs.

**Figure S2.**
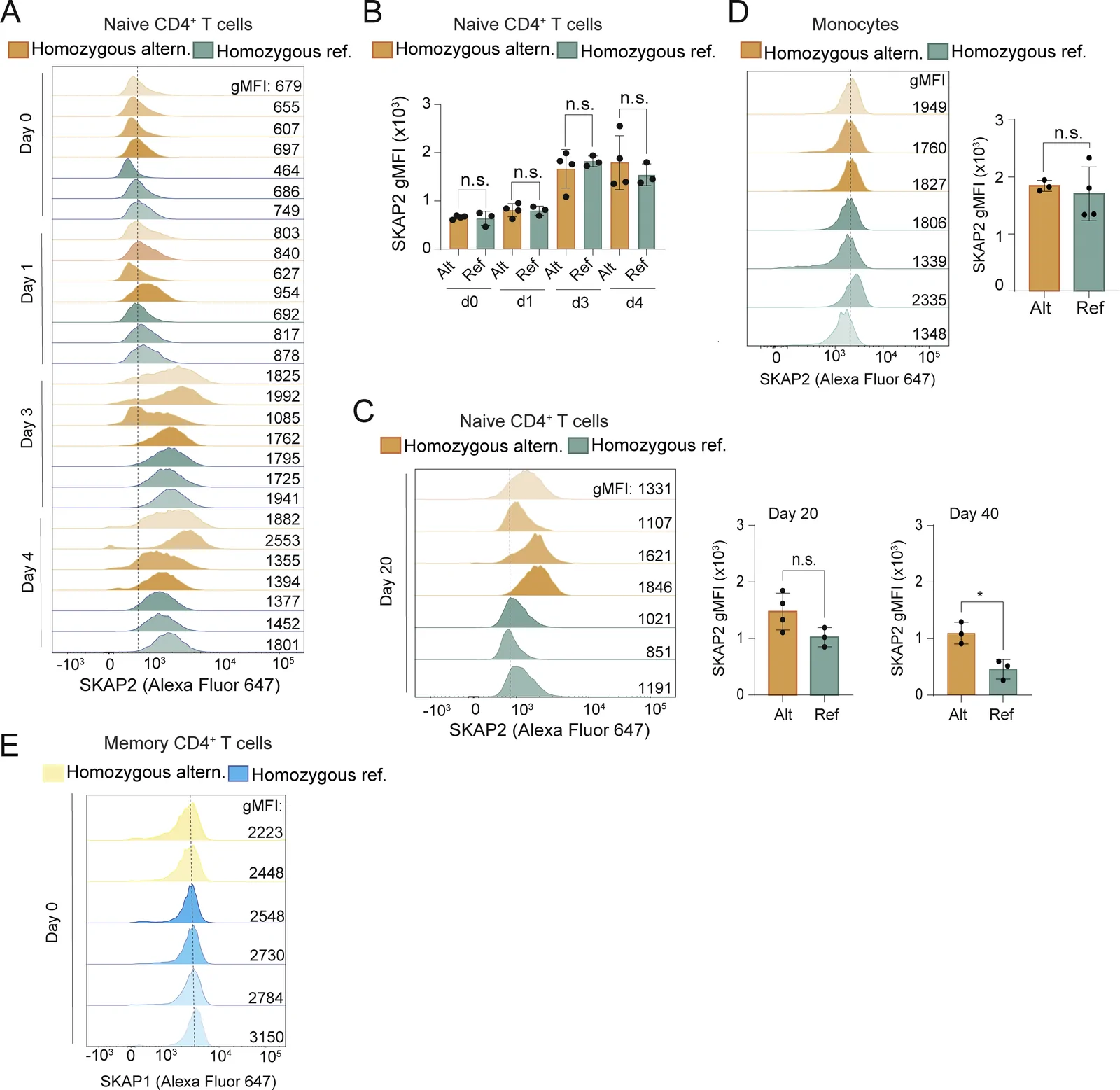
**Genotype-dependent SKAP2 levels in T lymphocytes. (A and B)** Intracellular staining for SKAP2 expression in CD4^+^ naïve T cells from *n* = 4 donors carrying the alternative allele (shades of orange) and *n* = 3 donors carrying the reference allele (shades of green). SKAP2 protein levels are shown at day 0 and at different time points following stimulation with aCD3/aCD28. Each line represents one donor. Mean ± SD, unpaired *t* test, two-tailed. From left to right: n.s. P = 0.7412, n.s. P = 0.9153, n.s. P = 0.5519, and n.s. P = 0.5005. **(C)** SKAP2 intracellular staining of cells as in A and B at day 20 and day 40 after stimulation. Day 40 cells were restimulated once at day 20 with aCD3/aCD28. In each line, different color intensities represent different donors (alternative allele: shades of orange; reference allele: shades of green). Mean ± SD, unpaired *t*-test, two-tailed, n.s. P = 0.0808, *P = 0.0128. **(D)** Intracellular staining for SKAP2 expression in monocytes from donors homozygous for the alternative (*n* = 3, shades of orange) or the reference allele (*n* = 4, shades of green). Protein levels are shown as gMFI, mean ± SD, unpaired *t* test, two-tailed, n.s. P = 0.6457. **(E)** Intracellular staining for SKAP1 expression in CD4^+^ memory T cells from donors homozygous for the alternative (*n* = 2, shades of yellow) or the reference allele (*n* = 4, shades of blue). Each line represents one donor. gMFI, geometric mean fluorescence intensity.

To determine the impact of distinct genotypes on SKAP2 protein expression in T cells, we screened *n* = 217 healthy blood donors to identify individuals homozygous for the minor (alternative) and major (reference) alleles by TaqMan probe-based genotyping (Fig. S1 A). For this analysis, we used a single specific SNP (*rs17323934*) among the many in this locus because multiple neighboring SNPs are inherited together as a haplotype block, as shown by the high linkage disequilibrium across this genomic region. This was also confirmed by sequencing of several additional SNPs (Fig. S1 B). The SNP *rs10271306* was used as a control since it is not a reported eQTL, and in fact, it was not concordant with the other SNPs (Fig. S1 B). Naïve and memory CD4^+^ as well as memory CD8^+^ T lymphocytes from donors homozygous for the reference or minor allele were activated with plate-bound anti-CD3 and anti-CD28 antibodies (hereafter aCD3/aCD28), and SKAP2 expression was measured by intracellular staining. Compared with T lymphocytes carrying the reference allele, memory CD4^+^ and CD8^+^ T lymphocytes homozygous for the alternative allele expressed higher levels of SKAP2 in the resting state and early after activation, while expression at a later time point after activation was similarly induced in both genotypes (Fig. 1, E–G; gating strategy in Fig. S1 C). Instead, naïve CD4^+^ T cells expressed low and variable levels of SKAP2 early after the initial activation (∼1.2-fold at day 1 compared with day 0, in both genotypes), and the difference in SKAP2 expression between the two genotypes only emerged at later time points of culture (day 20–40), likely corresponding to the establishment of an effector/memory-like phenotype (Fig. S2). Consistent with the eQTL data, no significant difference in SKAP2 expression was observed in CD14^+^ monocytes from individuals homozygous for the reference or alternative allele (Fig. S2 D). In addition, SKAP1 expression was similar across CD4^+^ memory T cells of different genotypes (Fig. S2 E).

Overall, these data show a causal link between genotype and SKAP2 protein expression in human T cells.

### Increased SKAP2 expression induces cell cycle and adhesion gene expression programs

**Figure S3.**
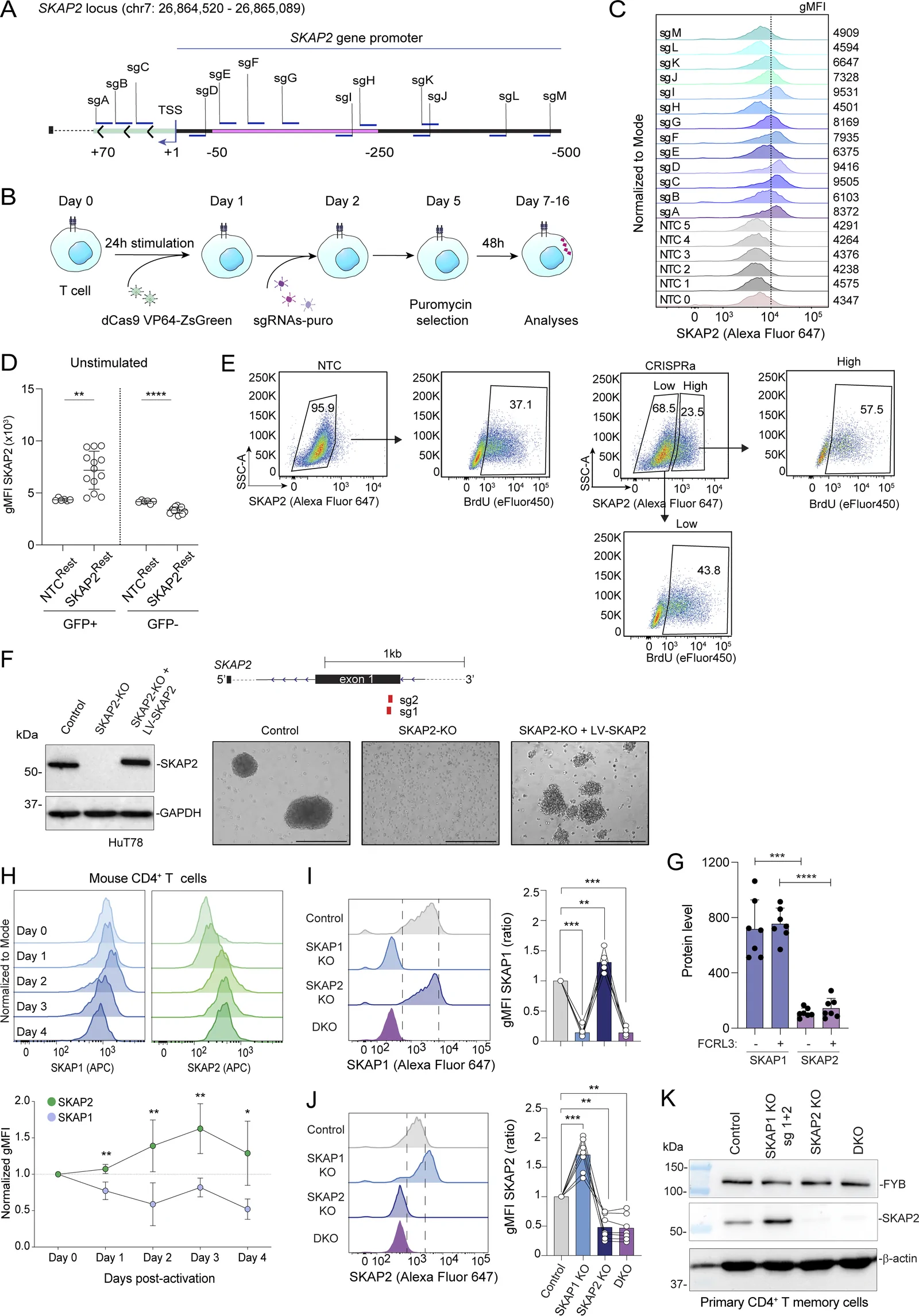
**CRISPRa efficiently induces *SKAP2* expression. (A)** Schematic representation of the *SKAP2* gene promoter region, indicating the sites targeted by the sgRNAs used for CRISPRa. The TSS is indicated by an arrow. **(B)** Experimental workflow for CRISPRa experiments in CD4^+^ T lymphocytes. After separation from peripheral blood, memory T cells were activated for 24 h with plate-bound aCD3/aCD28 antibodies, transduced with dCas9-VP64-ZsGreen lentiviral (LV) particles, followed by a second transduction, 24 h later, with sgRNA-puromycin lentiviruses. After expansion, cells were puromycin-selected at day 5 for 48 h, followed by sorting at day 7 to enrich GFP^+^ cells for downstream analyses. **(C)** SKAP2 expression in T cells from the CRISPRa experiment was determined by intracellular staining. CRISPRa was performed using CD4^+^ memory T cells and sgRNAs targeting the *SKAP2* gene promoter region or NTCs. One representative experiment is shown; each line represents one sgRNA or NTC. **(D)** SKAP2 expression measured in cells as in C. Both the GFP^+^ and GFP^–^ populations are shown. Unpaired *t* test, two-tailed. From left to right: **P = 0.0016, ****P < 0.0001. **(E)** Gating strategy for the analysis of T cell proliferation in CRISPRa-treated samples. CRISPRa-treated T cells transduced with either NTCs or with guide RNAs targeting the *SKAP2* promoter region (CRISPRa) were stained for SKAP2 protein expression, and the percentage of BrdU^+^ cells was determined in low- and high-SKAP2 expressing cells. **(F)** Schematic representation of sgRNAs locations for SKAP2-KO by CRISPR-Cas9 (top) and immunoblot showing SKAP2 expression in HuT78 cells (left). Cells were transfected with Cas9-sgRNAs RNPs targeting exon 1 of *SKAP2*. SKAP2 expression was also reconstituted by LV transduction. GAPDH: loading control. (Right) Cell clustering in HuT78 cells. Representative phase-contrast images are shown for WT, SKAP2-KO, and SKAP2-reconstituted HuT78 cells. Images were acquired with an EVOS Cell Imaging System at 10× magnification. Representative of *n* = 10 experiments. Scale bar: 400 µm. **(G)** Shotgun proteomics data from a previously published dataset (Bianchi et al., 2026), directly comparing SKAP1 and SKAP2 protein expression in resting CD8^+^ memory T lymphocytes freshly isolated from *n* = 7 donors. Each dot represents one donor. Cells were separated by sorting based on the surface expression of the FCRL3 receptor. Mean ± SD, paired *t* test, two-tailed. From left to right: ***P = 0.0002, ****P < 0.0001. **(H)** Intracellular staining for Skap1 (left) and Skap2 (right) expression in mouse T cells. Mouse CD4^+^ T cells were stimulated with aCD3/aCD28 for the indicated times, followed by intracellular staining. One representative result is shown on top, and the relative expression of both Skap1 and Skap2 upon activation of T cells from *n* = 6 mice in *n* = 2 independent experiments is shown in the lower panel. Mean ± SD. Two-way ANOVA. From left to right: **P = 0.004, **P = 0.0091, **P = 0.0068, and *P = 0.0333. **(I)** Intracellular staining for SKAP1 in control, SKAP1-KO, SKAP2-KO and DKO primary human T cells. One representative experiment is shown on the left, with the results of *n* = 6 individual donors on the right. Mean ± SD. Ratio paired *t* test. From left to right: ***P = 0.0002, **P = 0.0030, and ***P = 0.0001. **(J)** SKAP2 intracellular staining in control, SKAP1-KO, SKAP2-KO, and DKO primary human T cells. One representative experiment is shown on the left, with the results of *n* = 7 individual donors on the right. Mean ± SD. Ratio paired *t* test. From left to right: ***P = 0.0002, **P = 0.0029, and **P = 0.0028. **(K)** Western blot showing FYB and SKAP2 protein expression in control, SKAP1-KO, SKAP2-KO, and DKO human T cells. Representative of *n* = 2 independent experiments. Source data are available for this figure: SourceData FS3.

**Figure 2.**
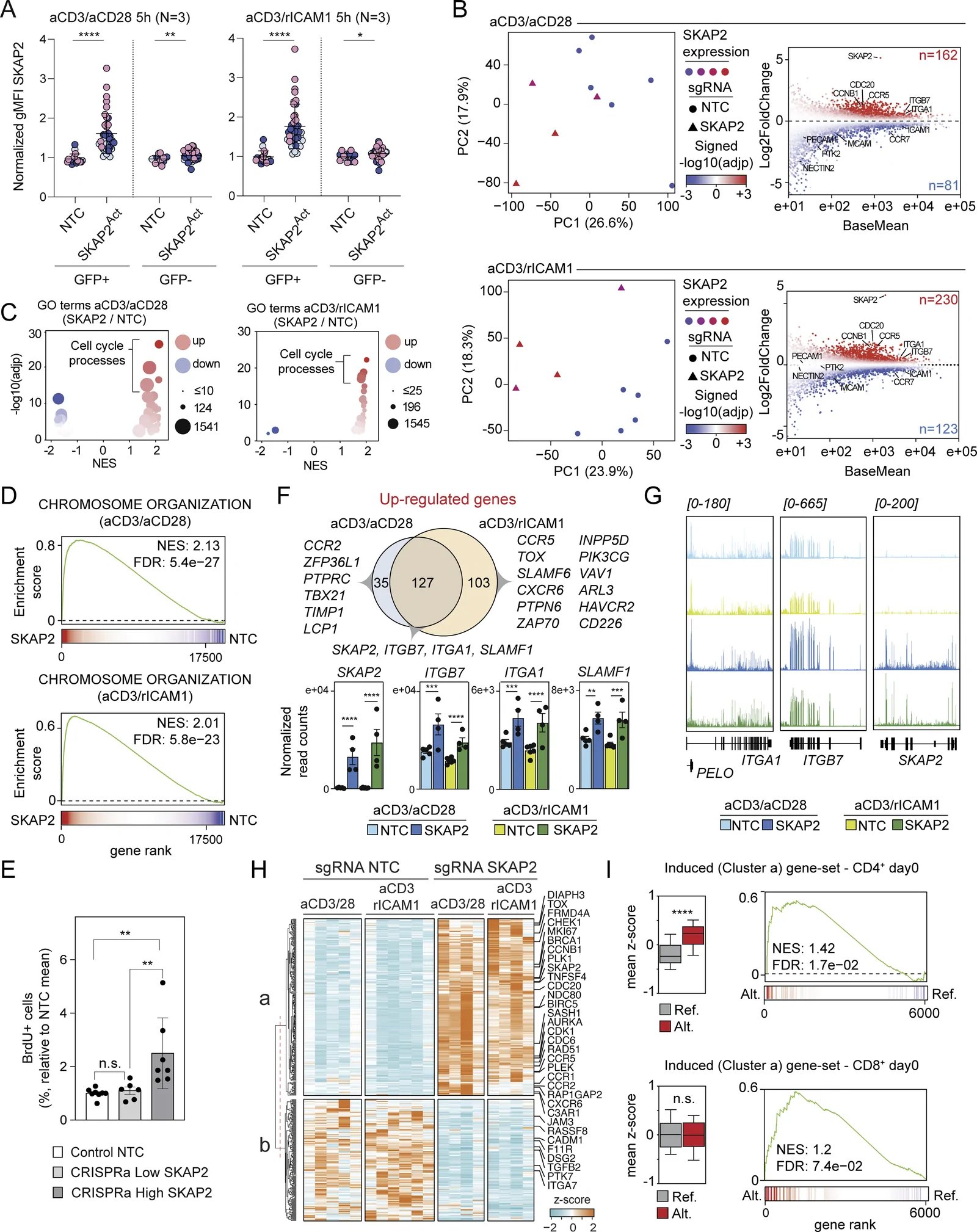
**Induction of SKAP2 expression by CRISPRa modulates T cell proliferation. (A)** SKAP2 expression measured by intracellular staining in CRISPRa-treated T cells, stimulated for 5 h with aCD3/aCD28 antibodies or aCD3/rICAM1. *n* = 3 independent donors; each color represents one donor. Unpaired *t* test, two-tailed. From left to right: ****P < 0.0001, **P = 0.0071, ****P < 0.0001, and *P = 0.0246. **(B)** PCAs (left panels) and scatter plots showing differentially expressed genes (right panels) in CD4^+^ T cells in which endogenous SKAP2 expression was increased by CRISPRa using four different sgRNAs (used as biological replicates). Cells transduced with NTC sgRNAs are shown as controls. In the PCA, SKAP2 expression levels in distinct samples are indicated by different colors. Top: Stimulation with aCD3/aCD28; bottom: stimulation with aCD3/rICAM1. **(C)** Functional categories associated with differentially expressed genes in the same experiment. Color intensities report adj. P values (with higher intensities representing lower values). The complete list is reported in Data S2. **(D)** GSEA showing the impact of increased SKAP2 expression on a cell cycle–related signature. The genes in the lists were sorted (from left to right) based on their relative level of expression in cells transduced with SKAP2 sgRNAs versus cells transduced with NTC sgRNAs. **(E)** Proliferation in CRISPRa-treated T cells and controls was measured by a BrdU incorporation assay. Each dot represents one independent experiment. Mean ± SD. Unpaired or paired *t* test, two-tailed. From left to right: n.s. P = 0.4830, **P = 0.0072, **P = 0.0046. **(F)** Venn diagram showing the overlap between up-regulated genes in the two stimulation conditions used (top). Bar plot showing the magnitude of changes in RNA-seq read counts for *SKAP2*, *ITGA1*, *ITGB7*, and *SLAMF1* genes (bottom). Significance annotations correspond to DESeq2-adj. P values (Benjamini–Hochberg correction): ** FDR < 0.01; *** FDR < 0.001; **** FDR < 0.0001. **(G)** IGV snapshot of merged BigWig tracks across the indicated genes. **(H)** Heatmaps displaying genes differentially regulated by SKAP2 CRISPRa. Cluster a contains genes induced following *SKAP2* upregulation, while cluster b contains genes downregulated following *SKAP2* induction. Representative genes are indicated. **(I)** Comparison of gene expression signatures in CD4^+^ and CD8^+^ memory T cells from donors homozygous for the reference (Ref.) or the alternative (Alt.) allele. Top: CD4^+^ memory T cells. Top left: Box plot showing the mean *z*-score distribution of genes from cluster a (as defined in H) in reference versus alternative allele homozygous donors. Top right: GSEA illustrating the fold change impact of alternative allele donors over references across genes from cluster a. Bottom: CD8^+^ memory T cells. Bottom left: Box plot displaying the mean *z*-score distribution of genes from cluster a in donors homozygous for the reference versus alternative allele. Bottom right: GSEA showing the fold change impact of homozygosity for the minor allele over the reference allele. Statistical significance was assessed using a paired nonparametric Wilcoxon test (****P ≤ 0.0001). FDR, false discovery rate.

Since the risk variants found within the *SKAP2* locus correlated with high SKAP2 expression in T cells, we used CRISPRa to enhance *SKAP2* expression from its endogenous locus (Konermann et al., 2015; Schmidt et al., 2022). We designed 13 different sgRNAs targeting the *SKAP2* proximal promoter, spanning ∼500 bp around the transcription start site (TSS) (Fig. S3 A), and 6 sgRNAs as nontargeting controls (NTCs). Primary memory CD4^+^ T lymphocytes were stimulated with aCD3/aCD28 and sequentially transduced first with a lentivirus encoding the dCas9-VP64-ZsGreen transcriptional activator and then with lentiviruses encoding individual sgRNAs and the puromycin resistance gene. After puromycin selection and cell sorting to enrich GFP^+^ cells, T lymphocytes were expanded and restimulated, followed by intracellular staining to measure SKAP2 protein levels (schematic representation of the workflow shown in Fig. S3 B). Most *SKAP2*-targeting sgRNAs substantially increased SKAP2 protein expression compared with NTC sgRNAs (Fig. S3 C). A significant increase in SKAP2 expression was observed only within the GFP^+^ cells, which co-expressed the dCas9 protein together with *SKAP2* sgRNAs, and not in GFP^–^ cells or in cells (either GFP^+^ or GFP^–^) transduced with NTC sgRNAs (Fig. S3 D), indicating high efficiency and specificity of the experimental workflow. We repeated this experiment using T lymphocytes from *n* = 3 independent donors and confirmed significantly increased SKAP2 expression also upon T cell restimulation for 5 h with aCD3/aCD28 (canonical T cell co-stimulation), or anti-CD3 and recombinant ICAM1 (rICAM1), the adhesion ligand for the lymphocyte integrin LFA-1 (Fig. 2 A).

To comprehensively determine the effect of SKAP2 expression on T cells, we performed RNA sequencing (RNA-seq) comparing four highly active *SKAP2*-targeting sgRNAs with multiple NTCs. Principal component analysis (PCA) and unsupervised clustering revealed that the *SKAP2*-CRISPRa samples clearly separated from NTCs in response to aCD3/aCD28 and aCD3/rICAM1 stimulation (Fig. 2 B). In both experimental groups, approximately twofold more differentially expressed genes were upregulated than downregulated (Fig. 2 B). Several of these differentially expressed genes were part of two main well-defined biological programs, namely cell cycle (e.g., *CDC20*, *PLK1*, *CCNB1*, *CDK1*, and *NDC80*) and T cell trafficking, adhesion, and activation (genes encoding integrins and chemokine receptors, such as integrin-α1 (*ITGA1*)*,* integrin-β7 (*ITGB7*)*, CCR1*, *CCR2*, *CCR5*, *CXCR6*, *RAP1GAP2*, and *TNFSF4*), suggesting an impact of increased SKAP2 levels on the control of activation-induced T cell proliferation (Fig. 2, C and D; and Data S2). Consistent with these findings, analysis of T cell proliferation by BrdU incorporation assay in two additional independent donors revealed a robust increase in proliferation in cells expressing high levels of SKAP2, compared with cells with lower SKAP2 levels (Fig. 2 E, gating strategy in Fig. S3 E).

Next, we analyzed which differentially expressed genes in *SKAP2*-CRISPRa samples versus NTCs were shared between the two stimulation conditions (aCD3/aCD28 and aCD3/rICAM1). Apart from the cell cycle-related genes, shared upregulated genes included two integrin genes, *ITGA1* and *ITGB7*, the integrin essential for T lymphocyte homing to the gut (Wagner et al., 1996) (Fig. 2, F and G). In addition, signaling lymphocytic activation molecule 1 (*SLAMF1* or CD150), a molecule important for homo- and hetero-typic cell-cell interactions (Howie et al., 2002), was also concordantly upregulated in the *SKAP2*-CRISPRa samples. Several additional factors involved in migration and signaling were upregulated selectively or preferentially by each individual treatment, including chemokine receptors (*CCR2*, *CCR5*, *CCR1*, and *CXCR6*), TCR signaling or regulatory molecules (*ZAP70*, *VAV1*, and *PTPN6*, encoding the SHP1 phosphatase, and *CD226* encoding the inhibitory receptor TIGIT), transcription factors (*TOX*, *TBX21*, encoding T-bet), and RNA-binding proteins (*ZFP36L1*). Contrary to the upregulated genes, the downregulated genes common to both treatments were a few and only associated with general cellular functions (Data S2).

Finally, we determined whether the genes upregulated in *SKAP2*-CRISPRa T cells were also differentially expressed in T cells from donors with a genetically different background. We performed RNA-seq of CD4^+^ and CD8^+^ memory T cells from *n* = 4 donors homozygous for either the minor or the major *SKAP2* allele. To limit the impact of variability due to genetic differences between the donors that are not linked to the *SKAP2* locus, we first defined transcriptional modules associated with increased *SKAP2* expression in CRISPRa T cells (cluster a in Fig. 2 H). Cluster a included proliferation-, migration-, and trafficking-related genes induced upon increased SKAP2 expression, while cluster b consisted of adhesion- and cell junction–associated genes that were downregulated upon *SKAP2* upregulation (Fig. 2 H). We then asked whether this signature was recapitulated in donors homozygous for the minor *SKAP2* allele. The cluster a signature (genes upregulated in *SKAP2*-CRISPRa cells) was significantly upregulated in CD4^+^ memory T cells from donors homozygous for the minor *SKAP2* allele (Fig. 2 I, top). CD8^+^ memory T cells from the same donors showed no significant gene upregulation, although a clear trend toward an enrichment of this signature in the gene set enrichment analysis (GSEA) analysis was detected also in this cell subset (Fig. 2 I, bottom, and Data S2). This is consistent with the more prominent effect of eQTLs in this locus in CD4^+^ compared with CD8^+^ T cells (Fig. 1 C).

Overall, increased levels of SKAP2 expression impacted T cell responses, in particular cell cycle–related programs as well as trafficking and integrin-associated functions.

### Distinct requirements for SKAP2 and SKAP1 in T cell adhesion and migration

**Figure 3.**
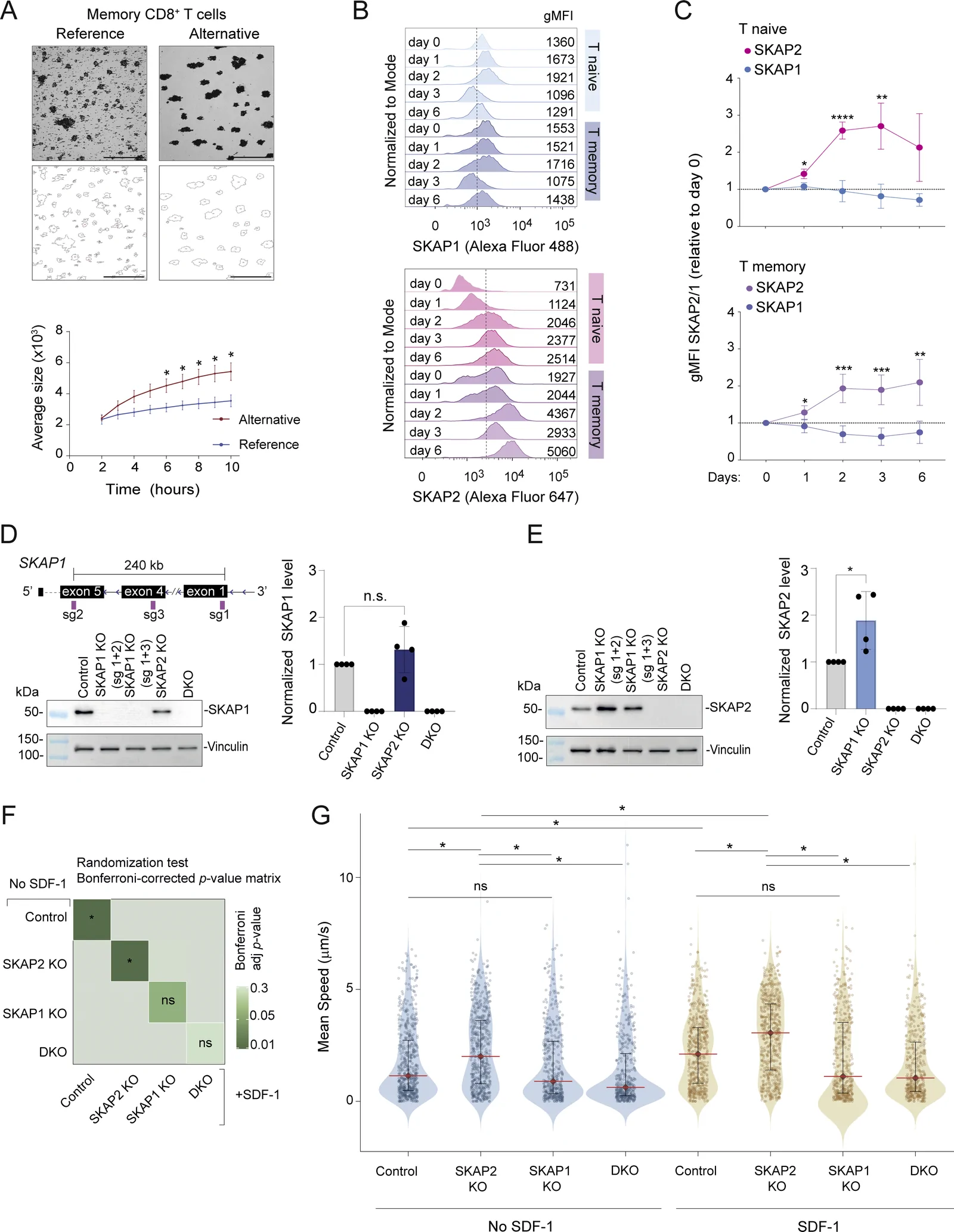
**SKAP1 and SKAP2 play distinct roles in the regulation of T cell adhesion and migration. (A)** Homotypic cell clustering analysis in human CD8^+^ T lymphocytes from donors carrying either the reference or the alternative *SKAP2* allelic variant. Cells were stimulated for 10 h with aCD3/aCD28-coated beads. Images were acquired every hour using a 10× objective. Representative brightfield images (top) and corresponding outlines of cell clusters (bottom) identified by the macro at 6 h following stimulation. Quantification and statistical analysis of data from *n* = 2 independent experiments and *n* = 4 donors with either genotype are shown on the right. Mean ± SEM, multiple unpaired *t* tests. From left to right: (2 h) n.s. P = 0.8304, (3 h) n.s. P = 0.2034, (4 h) n.s. P = 0.0793, (5 h) n.s. P = 0.0549, (6 h) *P = 0.0341, (7 h) *P = 0.0317, (8h) *P = 0.0262, (9h) *P = 0.0258, (10 h) *P = 0.0328. Scale bar: 500 µm. **(B)** Intracellular staining for SKAP1 (top) and SKAP2 (bottom) expression in T cells. Naïve and memory CD4^+^ T cells were stimulated with aCD3/aCD28 for the indicated times, followed by intracellular staining. One representative result of *n* = 5 (naïve) or *n* = 6 (memory) donors is shown. **(C)** Relative expression of SKAP1 and SKAP2 upon activation of naïve (top) and memory (bottom) T cells, by intracellular staining. Naïve T cells: *n* = 5 donors; memory T cells: *n* = 6 donors. Mean ± SD. Two-way ANOVA. Naïve T cells, from left to right: *P = 0.0102, ****P < 0.0001, **P = 0.0046. Memory T cells, from left to right: *P = 0.0244, ***P = 0.0007, ***P = 0.0008, **P = 0.0091. **(D)** Schematic representation (top, not to scale) of part of the *SKAP1* locus, with the locations of the sgRNAs used for CRISPR-editing indicated. Western blot analysis (bottom) of SKAP1 expression in control, SKAP1-KO, SKAP2-KO, and DKO primary T cells. One representative immunoblot is shown on the left, with the results of *n* = 4 individual experiments on the right. Mean ± SD. Ratio paired *t* test, n.s. P = 0.3921. **(E)** Western blot showing SKAP2 expression in control, SKAP1-KO, SKAP2-KO, and DKO primary T cells. One representative immunoblot is shown on the left, with the results of *n* = 4 individual experiments on the right. Mean ± SD. Ratio paired *t* test, *P = 0.0415. **(F and G)** Live imaging migration assay of primary T cells embedded in a 3D matrix of collagen, with or without addition of SDF-1 as chemoattractant. **(F)** Matrix summarizing Bonferroni-corrected P values derived from permutation testing for all pairwise comparisons shown, with and without SDF-1. *P = 0.028 (permutation test). **(G)** Violin plots showing the distribution of mean speed values in each indicated condition. Each dot represents one individual observation. *n* = 450–600 measurements from *n* = 3–4 donors (150 measurements/donor). Shown is the median (red horizontal line) with 25th and 75th percentiles (black vertical bars). Selected Bonferroni-corrected P values derived from permutation testing are indicated. The full matrices summarizing all pairwise comparisons are shown in Fig. S4 A. *P = 0.028 (permutation test). Source data are available for this figure: SourceData F3.

Since SKAP2 modulates cell adhesion in myeloid cells (Alenghat et al., 2012; Boras et al., 2017; Reinhold et al., 2009), we measured whether variation in SKAP2 expression might affect homotypic adhesion in T lymphocytes. Memory CD8^+^ T cells from donors homozygous for either the reference or the alternative *SKAP2* allele were plated as single-cell suspensions and stimulated with aCD3/aCD28-coated beads, while undergoing live imaging over 10 h. By measuring the average size of the cell clusters formed by T cells in culture, we found that T cells carrying the alternative allele (hence with higher SKAP2 expression) formed significantly larger clusters compared with T cells from subjects homozygous for the reference allele (Fig. 3 A), pointing toward a role of SKAP2 in the control of homotypic T cell adhesion. To further assess the impact of SKAP2 expression on adhesion, we deleted the *SKAP2* gene by CRISPR-Cas9 in the T cell line HuT78, which does not express SKAP1, hence eliminating this confounding factor. SKAP2 protein expression was undetectable in SKAP2-KO clones (Fig. S3 F). SKAP2 deletion almost completely abrogated the ability of HuT78 cells to form clusters, indicating a major defect in their adhesive capacity (Fig. S3 F). Cluster formation was restored upon re-expression of SKAP2 in SKAP2-KO cells by lentiviral (LV) transduction (Fig. S3 F).

Overall, these data point toward a key role of SKAP2 in modulating adhesion in T cells. However, primary T cells also express high levels of SKAP1. In fact, a proteomics dataset from human CD8^+^ T cells (Bianchi et al., 2026) showed that in resting T cells from *n* = 7 donors, SKAP1 was expressed at approximately sixfold higher levels compared with SKAP2 (Fig. S3 G). Hence, SKAP1 is most abundant in resting T cells. However, TCR activation of both naïve and memory T cells led to the strong and significant induction of SKAP2 protein expression, while SKAP1 levels remained stable or even slightly diminished (Fig. 3, B and C). Comparable kinetics were also observed in mouse T cells (Fig. S3 H), suggesting conservation of this regulatory pattern.

**Figure S4.**
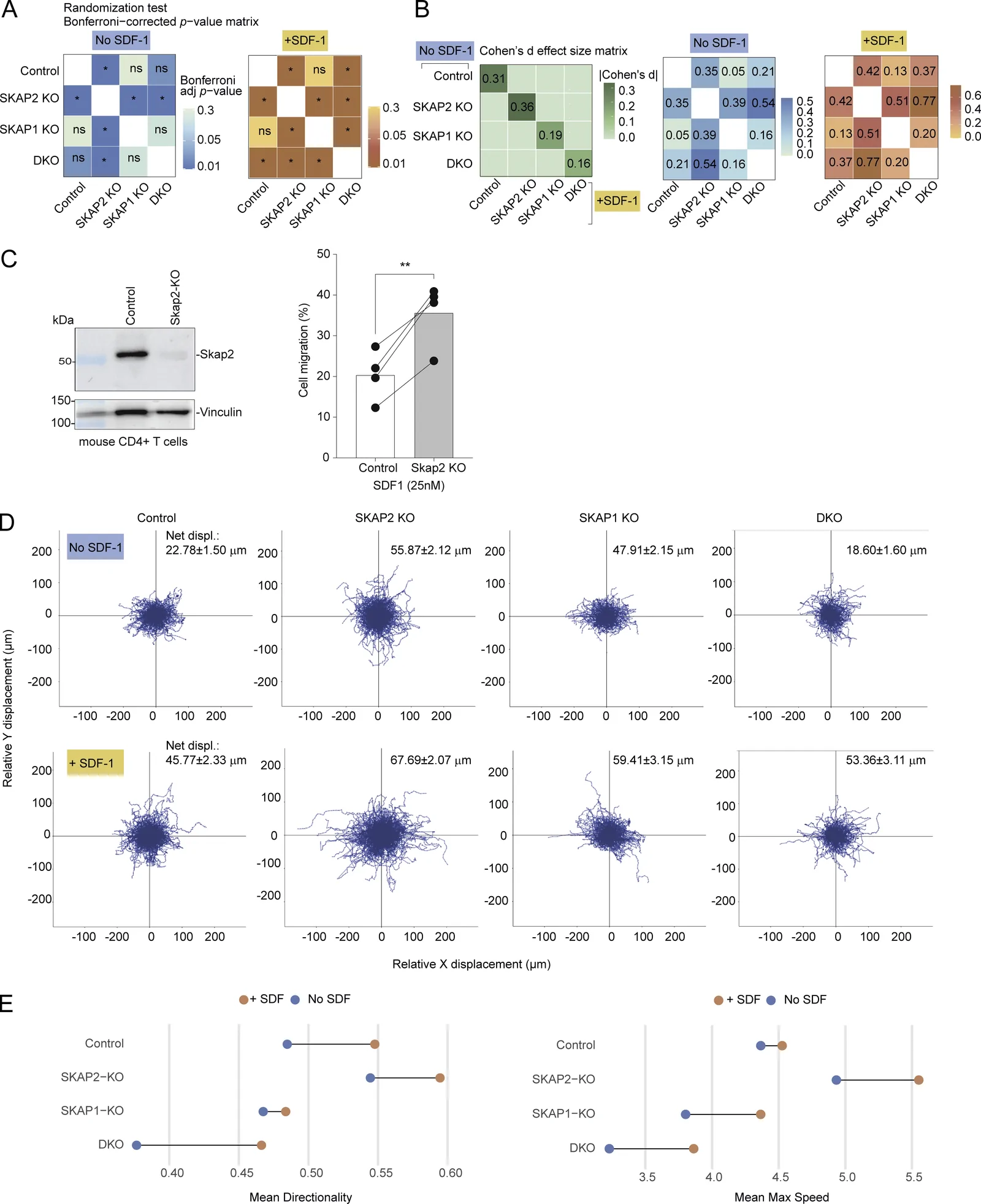
**SKAP2 restrains T cell** **migration** **. (A)** Matrices summarizing Bonferroni-corrected P values derived from permutation testing for all pairwise comparisons shown, with and without SDF-1, relative to Fig. 3 G. *P = 0.028 (permutation test). **(B)** Matrices summarizing Cohen’s *d* effect sizes for mean migration speed across all pairwise comparisons. **(C)** Transwell migration assay of primary murine CD4^+^ WT and *Skap2*-KO T cells. The western blot on the left shows the efficiency of Skap2 protein ablation (one representative immunoblot of *n* = 4 independent experiments). For the migration assay, T cells were seeded in the upper chamber of a transwell and left to migrate toward a gradient of SDF-1 (25 nM, in the bottom chamber) for 3 h. Each dot represents one mouse. The % of cell migration was calculated as the number of cells in the bottom well/the total number of cells × 100. *n* = 4 mice in *n* = 2 independent experiments. Mean, paired *t* test, two-tailed, **P = 0.0077. **(D)** Spider plots depicting T cell migration trajectories under the indicated conditions. Trajectories were ordered by time and filtered to exclude non-moving tracks. For each trajectory, x and y coordinates were mean-centered, transformed into polar coordinates, and replotted in Cartesian space to normalize track origins while preserving movement patterns, enabling direct comparison of migration trajectories across cells. Euclidean displacement from the origin was calculated at each time point as $d=\sqrt{x^{2}+y^{2}}$. Values are shown as mean ± SEM. **(E)** Dumbbell plots illustrating changes in directionality (left) and maximal instantaneous speed (right) for the control, SKAP2-KO, SKAP1-KO, and DKO T cells, comparing conditions with and without SDF-1. Source data are available for this figure: SourceData FS4.

To investigate the relative function of each of these proteins in T cells, we used CRISPR-Cas9–mediated editing to generate primary human T cells lacking either or both proteins at the same time (SKAP1-KO, SKAP2-KO, and double KO, DKO). Ablation of protein expression was very efficient in all cases, as shown by immunoblot and intracellular staining (Fig. 3, D and E; and Fig. S3, I and J). SKAP1 expression was only modestly affected in SKAP2-KO cells (∼30% increase, Fig. 3 D and Fig. S3 I ). On the contrary, SKAP1 ablation led to a large increase (71%) in SKAP2 expression, indicating that SKAP1 somehow limits SKAP2 levels in T cells (Fig. 3 E and Fig. S3 J). The expression of FYB, an adapter protein known to interact with both SKAP1 and SKAP2 (Voisinne et al., 2019), was not affected (Fig. S3 K). To further explore the role of these two proteins in T cell migration, we optimized a live cell-imaging assay in which primary human T cells were embedded in a 3D collagen matrix and then tracked in the presence or absence of SDF-1 as a chemoattractant. We analyzed several distinct readouts, including migration speed and directionality. Analysis of the mean migration speed across *n* = 6 independent donors revealed that control cells displayed minimal basal motility, which increased significantly upon SDF-1 stimulation (Fig. 3, F and G). Similarly, SKAP2-KO cells enhanced their mean migration speed in response to a chemoattractant (Videos 1, 2, and 3), while both SKAP1-KO and DKO cells lost their ability to enhance migration speed in response to SDF-1 (Fig. 3, F and G). Importantly, SKAP2-KO cells showed markedly elevated mean speed both without and with SDF-1, indicating that SKAP2 restrains both basal and chemokine-stimulated motility (Fig. 3 G and Fig. S4, A–E). Effect sizes for the mean speed were small-to-moderate when comparing SKAP2-KO with control cells (Cohen’s *d* ≈ 0.2–0.5) and moderate-to-large relative to SKAP1-KO or DKO cells (*d* > 0.5), underscoring the magnitude of this phenotype (Fig. S4 B). A comparable result was obtained using primary murine T cells, in which *Skap2* was similarly ablated by CRISPR-Cas9–mediated editing. Skap2-deficient T cells migrated more efficiently toward SDF-1 in transwell assays (Fig. S4 C), indicating conservation of this hypermigratory phenotype across species.

**Video 1.** **Basal motility of control human CD4** ^**+**^ **T cells. Images were acquired every 40 s for 1 h and are displayed at 7 frames per second (280X playback speed)**

**Video 2.** **Basal motility of SKAP1-KO human CD4** ^**+**^ **T cells. Images were acquired every 40 s for 1 h and are displayed at 7 frames per second (280X playback speed)**

**Video 3.** **Basal motility of SKAP2-KO human CD4** ^**+**^ **T cells. Images were acquired every 40 s for 1 h and are displayed at 7 frames per second (280X playback speed)**

In contrast, SKAP1-KO and DKO cells showed no significant increase in mean speed in response to SDF-1 (Fig. 3, F and G; and Fig. S4, A–E), indicating that SKAP1 is specifically required for chemokine-induced acceleration and that SKAP2 cannot compensate for its absence, despite its increased expression in SKAP1-KO cells. To analyze migration directionality, we calculated the Euclidean net displacement (straight-line distance from start to end point) for the same trajectories. These analyses revealed that cells of all genotypes retained some levels of SDF-1-driven directional migration. However, SKAP2-KO cells showed enhanced migratory output across all conditions and parameters tested (Fig. S4 D).

SKAP2 was previously shown to be critical for neutrophil extravasation, and in its absence, neutrophils showed increased rolling velocities associated with defects in firm adhesion to the endothelial surface and extravasation (Boras et al., 2017). Because slow rolling is also a prerequisite for firm arrest of T cells and subsequent trans-endothelial migration, we measured rolling directionality and maximal speed in the same experiment. Although these parameters were developed to quantify leukocyte rolling under flow, here they were applied to 3D migration trajectories to describe directionality and maximal instantaneous migration speed. Consistent with their migration speed, SKAP2-KO (but not SKAP1-KO or DKO) cells displayed a pronounced increase in directionality and maximal speed relative to control cells (Fig. S4 E), confirming that SKAP2 loss broadly enhances migratory output across all parameters measured and further reinforcing the notion of opposing and nonredundant roles of SKAP1 and SKAP2 in regulating T cell migratory dynamics.

Together, these findings indicate that SKAP1 is required for the optimal chemokine-induced increase in migration speed. SKAP2, by contrast, restrains migratory output, consistent with its role in promoting cell adhesion. The absence of a hypermigratory phenotype in DKO cells further indicates that the increased migration caused by the loss of SKAP2 is SKAP1-dependent, pointing to a functional division of labor between these two adaptors in the control of T cell motility.

### SKAP1 and SKAP2 control distinct transcriptional programs and are part of shared and private protein complexes

Results shown above indicate that SKAP1 and SKAP2 control T cell adhesion and migration in different and, to some extent, opposing manners, which may also result in distinct gene expression outputs. To determine whether the absence of either SKAP1 or SKAP2 brings about different downstream transcriptional programs, we performed RNA-seq using SKAP1-KO, SKAP2-KO, and DKO primary CD4^+^ T cells, in resting conditions or after re-stimulation with aCD3/aCD28 for 2 and 24 h.

**Figure 4.**
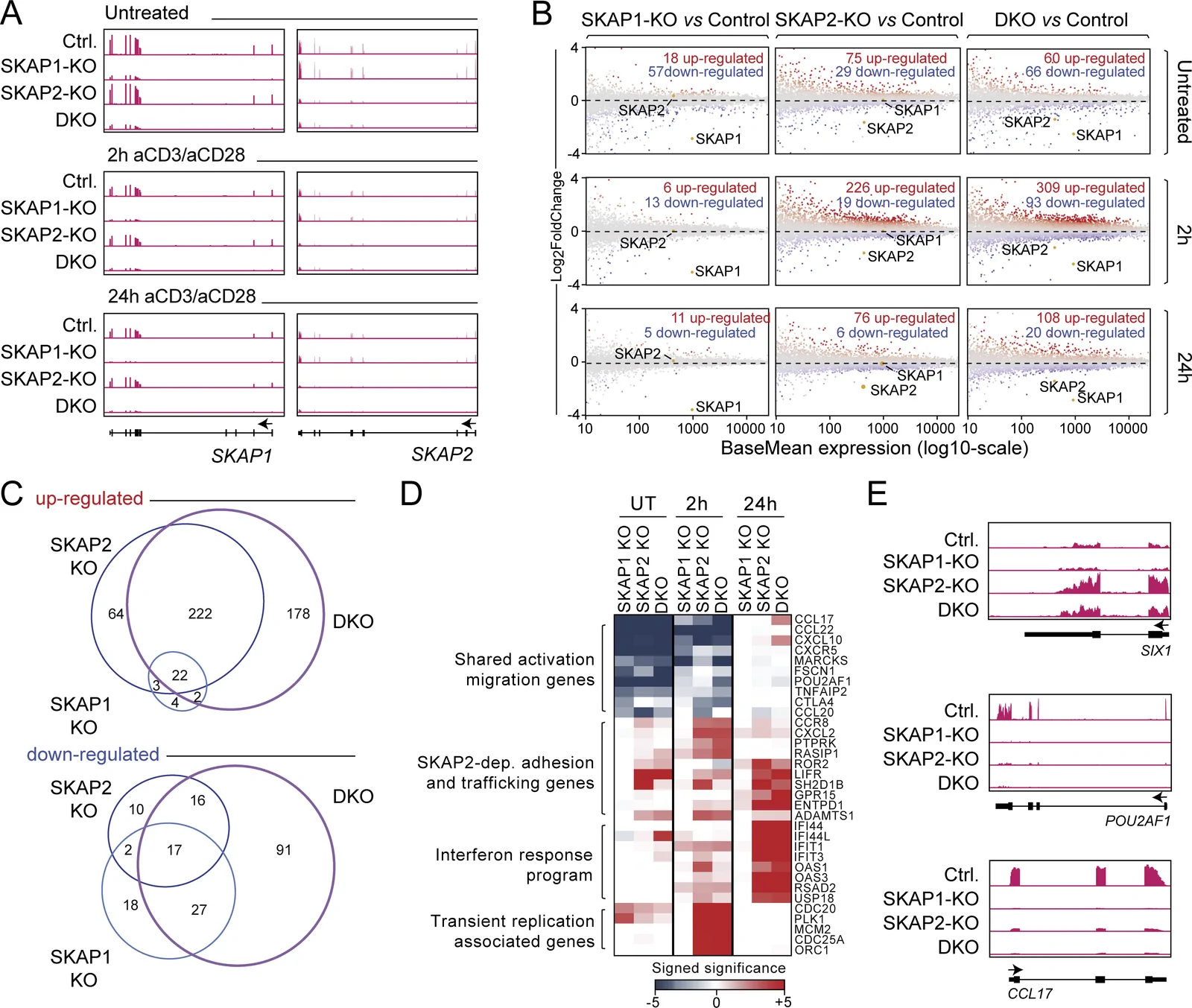
**SKAP2 deficiency drives broad transcriptional reprogramming. (A)** RNA-seq coverage tracks across the *SKAP1* and *SKAP2* loci in control, SKAP1-KO, SKAP2-KO, and DKO cells in untreated (UT), 2-h, and 24-h aCD3/aCD28 stimulation conditions. Loss of transcript coverage confirms successful gene disruption (*n* = 4 independent donors). **(B)** MA plots showing differential gene expression in SKAP1-KO, SKAP2-KO, and DKO cells relative to control cells in UT, 2-h, and 24-h stimulation conditions. Red and blue points indicate significantly upregulated and downregulated genes, respectively (FDR < 0.01). **(C)** Overlap analysis of significantly upregulated (top) and downregulated (bottom) genes across SKAP1-KO, SKAP2-KO, and DKO cells. **(D)** Heatmap of representative differentially expressed genes grouped into biologically coherent transcriptional modules. Values represent a signed differential expression score calculated as sign(log_2_ fold-change) × −log_10_(FDR), where positive values indicate increased expression and negative values indicate decreased expression relative to control cells. **(E)** Representative genome browser tracks illustrating genes representative of distinct transcriptional modules shown in D. FDR, false discovery rate.

First, *SKAP1* transcript levels remained largely unchanged in SKAP2-KO cells, while *SKAP2* transcript levels were only minimally affected in SKAP1-KO cells, indicating limited transcriptional cross-regulation between the two loci (Fig. 4 A and Data S3). This observation suggests that the increase in SKAP2 protein abundance in SKAP1-KO T cells (Fig. 3 E) was likely mediated by posttranscriptional mechanisms. Second, the deletion of *SKAP1* resulted in overall low-amplitude, low-magnitude changes in gene expression, with only a small number of genes being modestly deregulated, and with a prevalence of downregulation events in untreated cells (18 genes upregulated versus 57 downregulated) (Fig. 4 B). In contrast, *SKAP2* deletion produced transcriptional effects that were approximately one order of magnitude higher than those observed upon *SKAP1* deletion, with a strong prevalence of upregulation events and a maximal response at 2 h after simulation (226 genes upregulated versus 19 downregulated) (Fig. 4 B). In keeping with the independent effects of the two individual KOs, the DKO tended to produce mostly additive effects. In fact, a direct comparison of differentially expressed genes across SKAP1-KO, SKAP2-KO and DKO cells revealed that the majority of genes upregulated in DKO cells overlapped those deregulated in SKAP2-KO cells, indicating that the dominant transcriptional component of DKO cells was largely accounted for by SKAP2 deficiency (Fig. 4 C). In contrast, downregulated genes showed broader overlap among all three perturbations, suggesting the existence of a shared SKAP-dependent transcriptional program. Hence, consistent with different functions in the control of T cell adhesion and migration, deletion of *SKAP1* or *SKAP2* led to markedly different transcriptional outcomes.

To identify functional categories associated with differentially expressed genes, we grouped them into biologically coherent transcriptional modules and visualized their behavior across untreated, 2- and 24-h stimulation conditions (Fig. 4 D). This analysis revealed four major transcriptional programs. First, a shared activation- and migration-associated program was consistently downregulated across SKAP1-KO, SKAP2-KO, and DKO cells. Representative genes within this module include various chemokines and their receptors (*CCL17*, *CCL22*, *CXCL10*, and *CXCR5*), and regulators of activation-dependent response and cytoskeletal remodeling such as myristoylated alanine-rich C kinase substrate (*MARCKS*)*,* fascin actin-bundling protein 1 (*FSCN1*), and TNF α-induced protein 2 (*TNFAIP2*), indicating that both SKAP proteins contribute to regulate common transcriptional programs linked to lymphocyte activation and migration. Second, a distinct SKAP2-regulated module was preferentially induced in SKAP2-KO and DKO cells and included genes involved in trafficking and immune signaling, such as *CCR8*, *CXCL2*, receptor-type tyrosine-protein phosphatase α (*PTPRK*), *RASIP1*, *ROR2* (receptor tyrosine kinase-like orphan receptor 2, an orphan transmembrane receptor), and *SH2D1B*, a SLAM family receptor adapter. The enrichment of this program in both SKAP2-KO and DKO cells, but not in SKAP1-KO cells, further supports the conclusion that SKAP2 loss is the principal driver of the broader transcriptional phenotype observed in DKO cells. Third, SKAP2-KO and DKO cells upregulated an IFN-responsive transcriptional program, particularly at 24 h following stimulation. This module included canonical IFN-response genes (e.g., *IFI44*, *IFIT1*, *IFIT3*, *OAS1*, *OAS3*, and *RSAD2*), suggesting that loss of SKAP2 alters inflammatory and cytokine-responsive pathways in activated T cells. Finally, we observed a transient replication-associated transcriptional signature (including, among others, *CDC20*, *PLK1*, *MCM2*, *CDC25A*, and *ORC1*) in SKAP2-KO and DKO cells at 2 h after stimulation. Because SKAP proteins are membrane-proximal adaptor molecules primarily involved in regulating adhesion, migration and signaling rather than in direct transcriptional control, we interpret this signature as a secondary consequence of altered cellular activation states rather than evidence for a direct role of SKAP2 in cell cycle regulation. Notably, this program was largely absent from SKAP1-KO cells and was transient in nature, further emphasizing the distinct transcriptional consequences of SKAP2 deficiency. Genome-browser tracks illustrating genes from the identified transcriptional modules are shown in Fig. 4 E, including the transcription factor *SIX1*, the gene *POU2AF1*, encoding the transcriptional coregulator OCA-B, whose role in T lymphocytes has been recently described (Hughes et al., 2025; Sun et al., 2024), and the chemokine CCL17.

**Figure S5.**
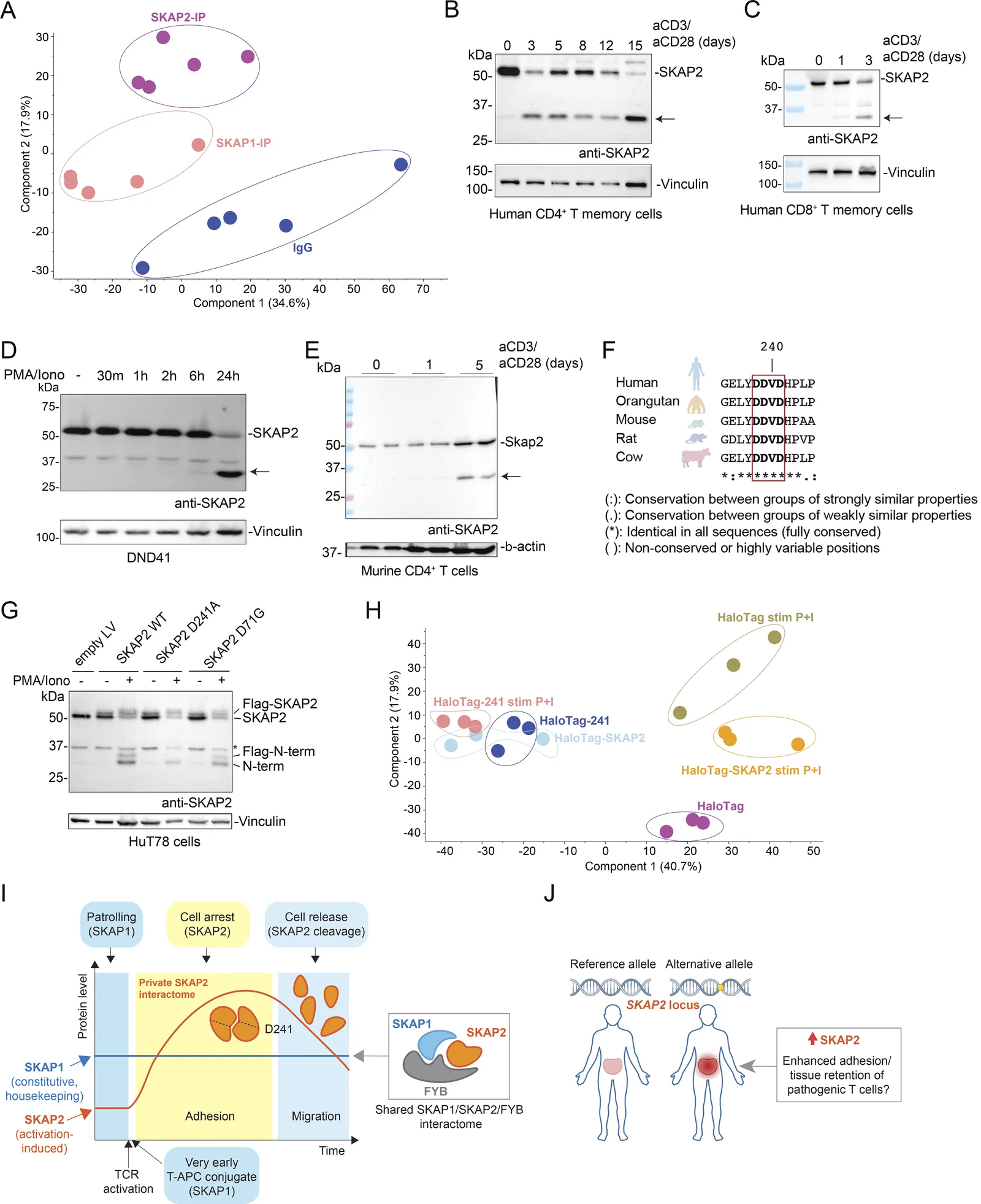
**SKAP2 expression and cleavage in different T cell subsets and conditions. (A)** PCA plot of the IP-MS data described in Fig. 5. **(B)** Immunoblot for SKAP2 expression in memory CD4^+^ T cells, resting or activated with aCD3/aCD28 antibodies for the indicated times. The arrow indicates a fast-migrating, low molecular weight form of SKAP2. Representative of *n* = 2 independent experiments. **(C)** Immunoblot analysis of SKAP2 expression in memory CD8^+^ T cells, resting or activated with aCD3/aCD28 antibodies for the indicated time points. Representative of *n* = 2 independent experiments. **(D)** SKAP2 expression was assessed by immunoblot in the DND41 T cell leukemia line. Cells were treated with PMA and ionomycin for the indicated times. Representative of *n* = 2 independent experiments. **(E)** The presence of the low molecular weight Skap2 protein was assessed by immunoblot in mouse CD4^+^ T cells isolated from the spleen and lymph nodes of C57Bl/6 mice. Cells were stimulated for the indicated times with aCD3/aCD28, followed by lysis and immunoblot analysis for Skap2 expression. Each lane represents a pool of two mice. *n* = 4 mice. **(F)** Conservation of the SKAP2 protein cleavage site across species. Sequence alignment was performed using Clustal Omega (Madeira et al., 2024). **(G)** SKAP2 cleavage was assessed by immunoblotting in HuT78 cells transduced to overexpress WT SKAP2 or its mutant versions D241A and D71G. Cells were either left resting or were stimulated for 24 h with PMA and ionomycin. All proteins were tagged with an N-terminal Flag. Immunoblotting was performed using an anti-SKAP2 antibody. Representative of *n* = 2 independent experiments. **(H)** PCA plot of the IP-MS data described in Fig. 6 K. **(I)** Model of results. In resting T cells, SKAP1 is expressed at higher levels compared with SKAP2, and it is not induced upon TCR engagement. Hence, it represents the housekeeping, constitutive SKAP protein, required for early events during T cell activation, and likely to be important also for patrolling, in the absence of a stimulus. T cell activation leads to the upregulation of SKAP2 protein expression. Such activation-induced upregulation enhances adhesion and restrains T cell migration, counteracting SKAP1 activity. Finally, at later times of activation, SKAP2 proteolytic cleavage disassembles its interacting network and releases SKAP1-mediated migration. While a complex comprising SKAP1, SKAP2, and FYB is likely to regulate common targets, we speculate that the SKAP2-only functions may be mediated by a private SKAP2 interactome. **(J)** Hypothetical role in intestinal inflammation. Because intestinal inflammation in IBD depends on the accumulation of pathogenic T cells in the gut mucosa, genetically determined variation in this pathway may influence disease biology and therapeutic response. Increased SKAP2 expression from the risk allele may alter the normal adhesion/recirculation of activated T cells and favor their persistence within tissues in CD. Figure generated with FigureLabs. Source data are available for this figure: SourceData FS5.

**Figure 5.**
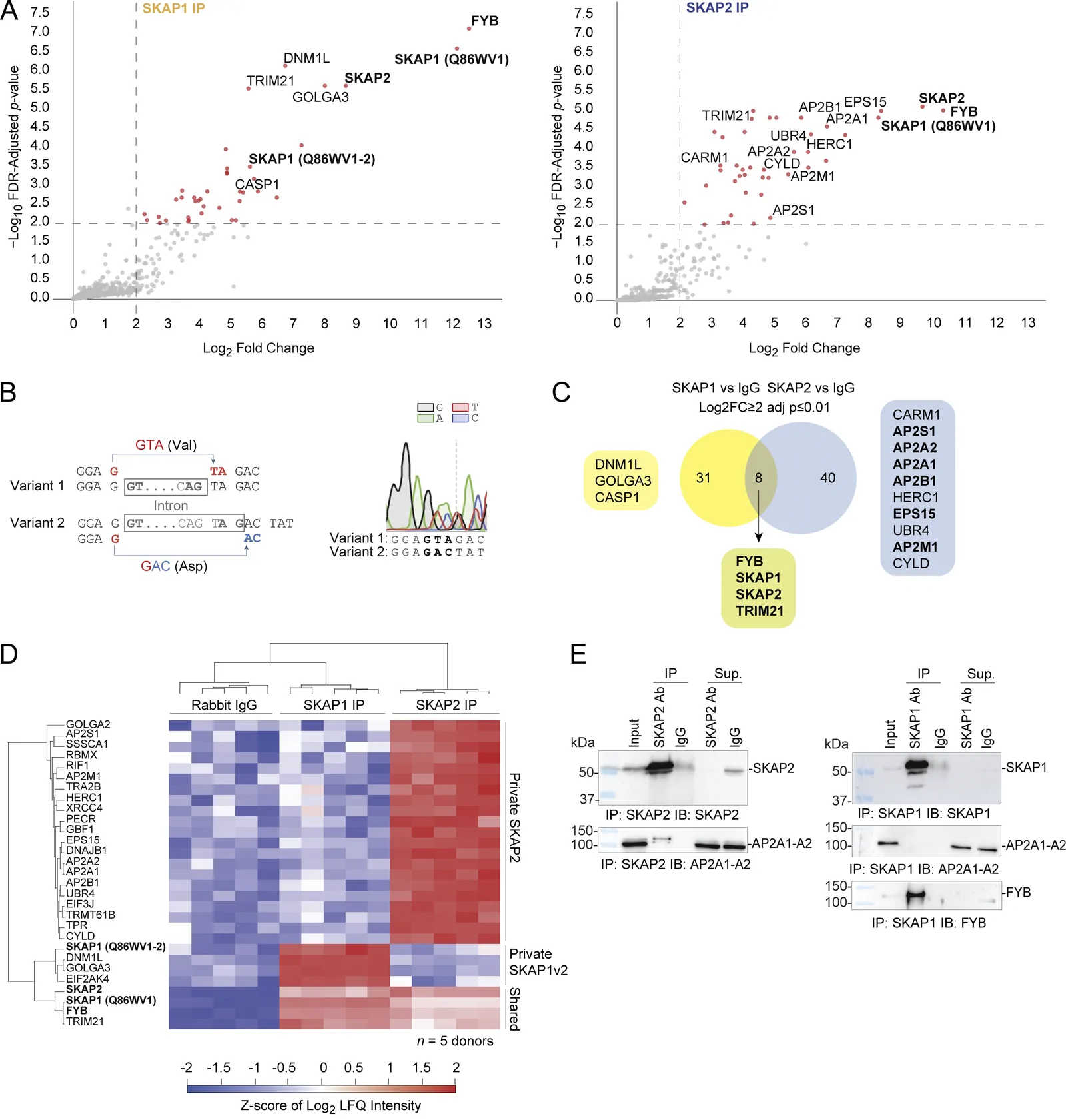
**SKAP1 and SKAP2 have unique interactomes in T cells. (A)** Scatter plots showing the results of IP-MS for SKAP1 versus control IgG (left) and SKAP2 versus control IgG. Thresholds: False discovery rate (FDR)-adj. P value (Benjamini–Hochberg correction) = 0.01 and log_2_FC = 2. *n* = 5 independent donors. **(B)** Schematic representation of alternative splicing in the *SKAP1* transcript around Val 293, and an example of RT-PCR and Sanger sequencing across the same region. Representative of *n* = 2 independent sequencing. **(C)** Venn diagram showing the overlap between proteins recovered in the IP-MS of both SKAP1 and SKAP2. Only proteins with log_2_FC ≥2 and adj. P value ≤0.01 compared with the control IgG were considered. **(D)** Heatmap of SKAP1 and SKAP2 interaction partners identified by IP-MS. Proteins significant by ANOVA after Benjamini–Hochberg FDR correction (FDR < 0.01) were retained, and proteins enriched in the IgG control condition were excluded. Unsupervised hierarchical clustering (Euclidean distance, average linkage) identified three groups of interactors enriched in SKAP1 IPs, SKAP2 IPs, or both. Within each cluster, proteins were further filtered for robust enrichment relative to control IgG (log_2_FC ≥ 4) and low variability within experimental groups (coefficient of variation, CV ≤ 0.03), applied to the corresponding condition(s). **(E)** IP and western blot confirming the interaction of components of the AP-2 complex with SKAP2 (left), but not SKAP1 (right) in primary CD4^+^ T cells. SKAP1 successfully co-immunoprecipitated with FYB, used as a control. Representative of *n* = 6 (SKAP2-IP) or *n* = 2 (SKAP1-IP) independent experiments. Source data are available for this figure: SourceData F5.

Because of the striking functional and transcriptional differences between SKAP1 and SKAP2 in T cells, we sought to determine whether they interact with distinct protein partners. Both SKAP1 and SKAP2 are known to be part of a complex with FYB (Voisinne et al., 2019), but whether they can also interact with additional, unique partners is unknown. We therefore performed immunoprecipitation and mass spectrometry (IP-MS) of SKAP1 and SKAP2 in primary CD4^+^ T cells, using cells from *n* = 5 independent donors. PCA showed correct clustering of the samples by antibody used in the IP (Fig. S5 A). Compared with IgG control, high levels of SKAP2 and FYB were recovered upon IP of SKAP1. Reciprocally, the IP of SKAP2 recovered with high efficiency both SKAP1 and FYB (Fig. 5 A and Data S4). Hence, SKAP1, SKAP2 and FYB can interact with each other. The anti-SKAP1 antibody recovered two described variants of SKAP1: the main variant (Q86WV1) as well as a second variant (Q86WV1-2) that differ by the lack of one single amino acid (V293) due to the alternative usage of two 3′ splice acceptor sites at exon 11 (Fig. 5 B). Unexpectedly, only the main SKAP1 isoform (Q86WV1) was recovered in the IP of SKAP2 (Fig. 5 A). This difference could not be promptly attributed to differences in the expression levels of the two variants. In fact, RT-PCR and Sanger sequencing of the mRNAs encoding the *SKAP1* variants revealed that both are expressed in T cells at relatively comparable levels (Fig. 5 B). This points toward a role for V293 in stabilizing the interactions of SKAP1 with protein partners. Indeed, amino acids 290–295 of SKAP1 are juxtaposed to an SH3 domain and are required for its interaction with FYB (UniProt, https://www.uniprot.org).

Unsupervised clustering further suggested that SKAP1 and SKAP2 on the one hand share some common interactors, and on the other, interact with their unique, private protein network. The shared complex included SKAP1 (main variant, Q86WV1), SKAP2, FYB, and TRIM21 (Fig. 5, C and D) primarily. TRIM21 is an E3 ubiquitin ligase essential for immune regulation, and was shown to act as a negative regulator of intestinal mucosal inflammation in IBD patients (Zhou et al., 2018). While the main SKAP1 isoform was incorporated in the SKAP1/SKAP2/FYB/TRIM21 complex, private SKAP1 interactions clustered with the alternative SKAP1 isoform (Q86WV1-2) and occurred only with relatively few proteins, including caspase-1 (CASP1), the dynamin-like protein DNM1L, involved in mitochondrial fission, and golgin-A3 (GOLGA3), a protein reported to be involved in maintaining the Golgi structure (Fig. 5, C and D). Unlike SKAP1, private SKAP2 interactions included several E3 ubiquitin ligases (HERC1, UBR4), the deubiquitinase CYLD and other enzymes such as the arginine methyltransferase CARM1. The most notable components of the private SKAP2 interactome, however, were multiple proteins of the AP-2 complex for clathrin-mediated endocytosis, as well as the AP-2 complex-associated protein EPS15, which is required for ligand-regulated receptor endocytosis (Fig. 5 A). This preferential interaction of SKAP2 (but not SKAP1) with the AP-2 complex was also independently confirmed by co-IP and immunoblot (Fig. 5 E). Hence, SKAP1 and SKAP2 are part of both common and private protein complexes.

Overall, we established that SKAP1 and SKAP2 have exclusive, nonredundant functions in T cells, which are likely to be mediated through their distinct protein–protein interaction networks.

### Caspase-mediated cleavage of SKAP2 upon T cell stimulation disrupts its interactome

**Figure 6.**
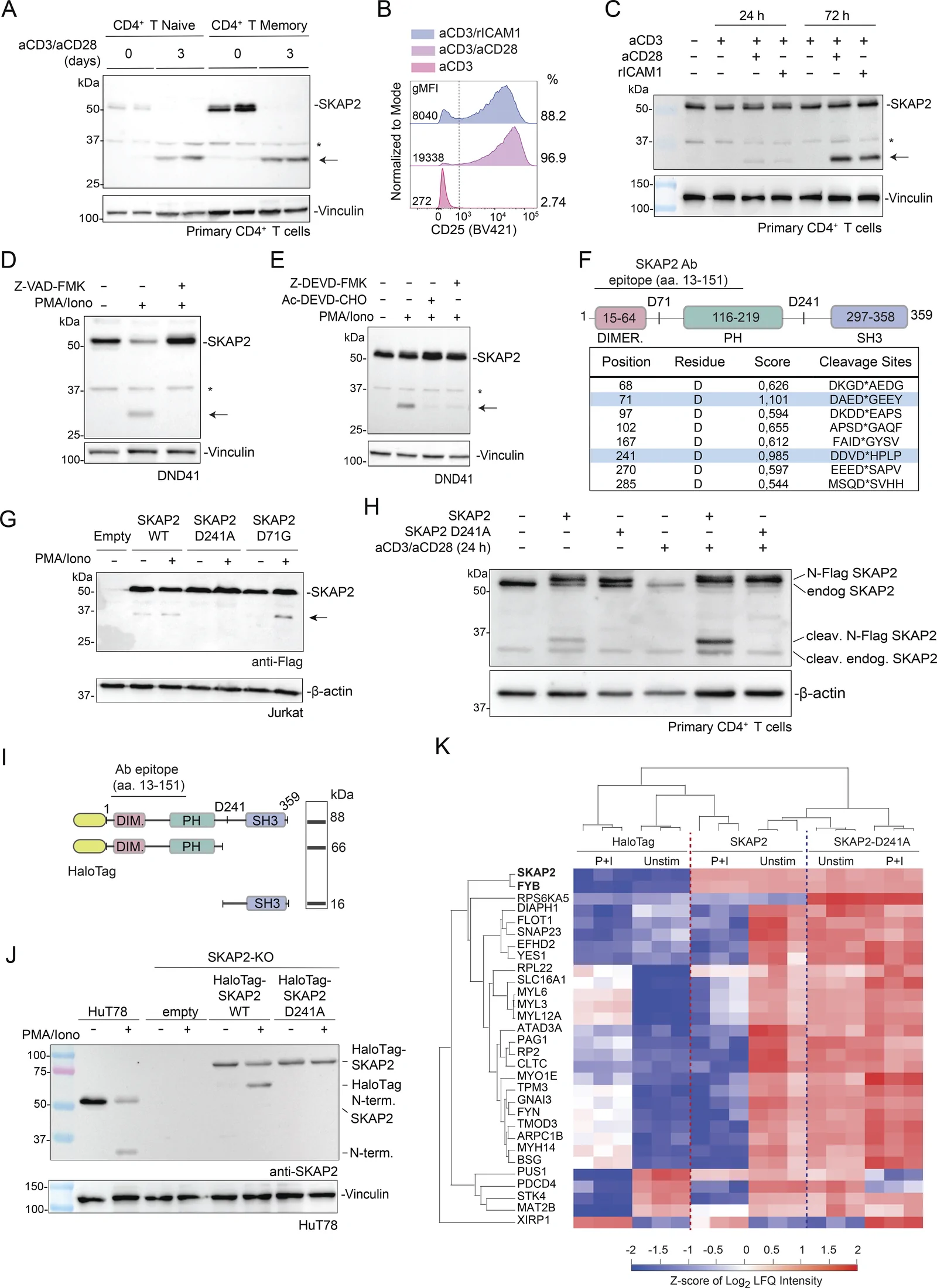
**SKAP2 proteolytic cleavage in response to stimulation leads to interactome disassembly. (A)** Immunoblot showing SKAP2 expression in naïve and memory CD4^+^ T lymphocytes, resting or activated for 3 days with plate-bound aCD3/aCD28 antibodies. Duplicate lanes contain two different, independent donors. The arrow indicates the low molecular weight SKAP2 species appearing only after stimulation. The asterisk (*) indicates a non-specific band. Representative of at least *n* = 4 independent experiments. **(B)** Surface staining to measure CD25 expression in T cells stimulated for 72 h with the indicated stimuli. **(C)** Immunoblot showing SKAP2 expression in T cells as in B, stimulated for 24 or 72 h. The fast-migrating SKAP2 band is indicated by an arrow. The asterisk (*) indicates a nonspecific band. Representative of *n* = 2 independent experiments. **(D)** Effects of the global caspase inhibitor Z-VAD-FMK on the SKAP2 protein in the human T cell line DND41, resting or stimulated with PMA and ionomycin for 24 h. The faster-migrating SKAP2 band is indicated by an arrow. Asterisk: nonspecific band. Vinculin is shown as a loading control. Representative of *n* = 2 independent experiments. **(E)** Effects of the caspase inhibitors Z-DEVD-FMK and Ac-DEVD-CHO on the SKAP2 protein in DND41 cells resting or stimulated with PMA and ionomycin for 24 h. The arrow indicates the low molecular weight SKAP2 species appearing only after stimulation. The asterisk (*) indicates a nonspecific band. Representative of *n* = 2 independent experiments. **(F)** Schematic representation of SKAP2 protein domains, including the dimerization domain (DIMER), the PH domain, the SH3 domain, and the locations of potential caspase cleavage sites (D71 and D241). The location of the epitope (amino acids 13–151) used to raise the anti-SKAP2 antibody used throughout the study is shown. The table below lists the potential caspase cleavage sites within the SKAP2 protein, as determined by the Cascleave tool. Asterisks (*) represent the predicted site of cleavage. The highest-ranking potential caspase cleavage sites are highlighted in blue. **(G)** Immunoblot to assess SKAP2 cleavage upon mutation of D241 or D71. Aspartic acid residues at positions 241 and 71 of SKAP2 were mutated to alanine or glycine. Jurkat cells were transduced to express SKAP2 (fused to an N-terminal Flag epitope), either WT or mutated at the two putative caspase cleavage sites, followed by stimulation for 24 h with PMA and ionomycin to induce cleavage. Immunoblot was performed with an anti-Flag antibody. β-actin is shown as a loading control. The arrow indicates cleaved SKAP2. Representative of *n* = 3 independent experiments. **(H)** Immunoblot to assess D241A SKAP2 cleavage in primary CD4^+^ T memory lymphocytes. T cells were transduced to overexpress either N-Flag-SKAP2 or the mutant N-Flag-SKAP2-D241A. Transduced cells were enriched by FACS sorting for GFP expression 7 days later. 10 days later, cells were either left resting or re-stimulated for 24 h with aCD3/aCD28, followed by immunoblotting with an anti-SKAP2 antibody. Representative of *n* = 2 independent experiments. **(I)** Schematic drawing illustrating the HaloTag-SKAP2 fusion and the fragments generated by its cleavage. **(J)** Expression of the HaloTag-SKAP2 fusion protein (WT or D241A mutant) in SKAP2-KO HuT78 cells, reconstituted by LV transduction and stimulated with PMA and ionomycin for 24 h, as indicated. WT HuT78 cells are included as a control. Representative of *n* = 3 independent experiments. **(K)** Heat map showing unsupervised hierarchical clustering of ANOVA-significant (permutation-based FDR < 0.01) MS results from HaloTag affinity purification in SKAP2-KO HuT78 cells transduced with HaloTag-SKAP2, HaloTag-SKAP2-D241V or HaloTag alone, either unstimulated or after 24 h of stimulation with PMA and ionomycin. *n* = 3 independent experiments. FDR, false discovery rate. Source data are available for this figure: SourceData F6.

By intracellular staining, we found that SKAP2 protein expression increased upon T cell activation (Fig. 3, B and C). Surprisingly, western blot analyses revealed the disappearance of the main SKAP2 band and the concomitant appearance of a low molecular weight (∼32 kDa) SKAP2 species that became prominent 3 days after stimulation (Fig. 6 A) and remained highly abundant at later time points after activation (Fig. S5 B). The ~32 kDa isoform was also observed upon activation of CD8^+^ memory T cells (Fig. S5 C), as well as in T cell lines stimulated with PMA and ionomycin (Fig. S5 D) and in stimulated mouse T cells (Fig. S5 E). TCR stimulation of memory T cells, with or without concomitant co-stimulation with anti-CD28 antibody or rICAM1, showed that full activation with co-stimulation was necessary to induce the low molecular weight isoform. Indeed, cells treated with anti-CD3 antibody alone did not get fully activated, as determined by CD25 expression, and failed to generate it (Fig. 6, B and C). Since the appearance of the low molecular weight isoform was observed also across T cell lines (DND41, HuT78), these were used for some of the downstream biochemical analyses. In DND41 cells, TCR-dependent induction of the low molecular weight SKAP2 species, as well as the disappearance of the full-length 55-kDa species, were both completely inhibited by treatment with a general caspase inhibitor (N-benzyloxycarbonyl-Val-Ala-Asp(O-Me) fluoromethyl ketone [Z-VAD-FMK]), indicating that SKAP2 underwent caspase-mediated cleavage upon T cell activation (Fig. 6 D; note that the antibody used was directed against amino acids. 13–151 in the N-terminal half of SKAP2). A similar result was obtained using the more selective benzyloxycarbonyl-Asp(OMe)-Glu(OMe)-Val-Asp(OMe)-fluoromethylketone (Z-DEVD-FMK) and Ac-DEVD-CHO inhibitors, pointing toward caspase-3-mediated cleavage (Fig. 6 E).

The overall structural organization of SKAP2 (Fig. 6 F) consists of an N-terminal coiled-coil domain, which mediates homodimerization (DIMER) (Swanson et al., 2008), a middle pleckstrin homology (PH) domain, and a C-terminal SH3 domain. To identify putative caspase cleavage sites in SKAP2, we used the Cascleave tool (Caspase substrate cleavage site prediction) (Wang et al., 2014), which returned two high-scoring candidate sites at positions D71 and D241, respectively, with D241 being located between the PH and the SH3 domain (Fig. 6 F) and retaining high evolutionary conservation (Fig. S5 F). Mutation of D241 to alanine completely abrogated SKAP2 cleavage in transduced HuT78 and Jurkat cells (the latter do not express endogenous SKAP2), both basally and upon stimulation with PMA and ionomycin, while mutation of D71 to glycine had no detectable effects (Fig. 6 G and Fig. S5 G). A similar result was obtained upon expression of the mutant protein in primary T cells stimulated with aCD3/aCD28 (Fig. 6 H).

To gain further mechanistic insights into the role of SKAP2 cleavage in T cells, WT or D241A mutant SKAP2 were tagged at the N terminus with a HaloTag and re-expressed in SKAP2-KO HuT78 (Fig. 6, I and J). Protein expression and SKAP2 cleavage in response to stimulation were verified by western blot (Fig. 6 J). Affinity purification of WT or D241A SKAP2 with Halo-Trap beads coupled to MS was carried out before and after stimulation with PMA and ionomycin for 20 h. HaloTag pull-down efficiently retrieved FYB both before and after stimulation, highlighting its stable interaction with SKAP2 (Fig. 6 K and Data S5). SKAP1 was not observed since it is not expressed in these cells. In addition, SKAP2 retrieved an interactome dominated by Src family kinases (FYN and YES1), clathrin machinery components (CLTC), and cortical actin-myosin (ARPC1B, MYO1E, MYH14, TPM3, TMOD3, and multiple myosin light chains, e.g., MYL6/MYL3/MYL12A), pointing toward associations at the cell surface with the actin cortex. Notably, all of these interactions, except the one with FYB, were lost after 24 h of stimulation (Fig. 6 K, Fig. S5 H, and Data S5). Such widespread loss of SKAP2-interacting proteins in response to PMA/ionomycin was completely abrogated in the D241A mutant (Fig. 6 K, Fig. S5 H, and Data S5), indicating that the release of interactors from the FYB/SKAP2 complex is likely to be caused by the proteolytic cleavage of SKAP2 and the subsequent release of the C-terminal SH3 domain. However, since a fraction of uncleaved SKAP2 persisted even after stimulation, we cannot rule out that additional mechanisms, such as posttranslational modifications or cleavage of additional proteins within the complex, may also contribute to the loss of SKAP2-associated proteins upon stimulation.

Overall, following TCR stimulation, SKAP2 undergoes caspase-mediated proteolytic cleavage at D241, which is associated with the loss of protein–protein interactions.

### SKAP2 cleavage modulates T cell adhesion

**Figure 7.**
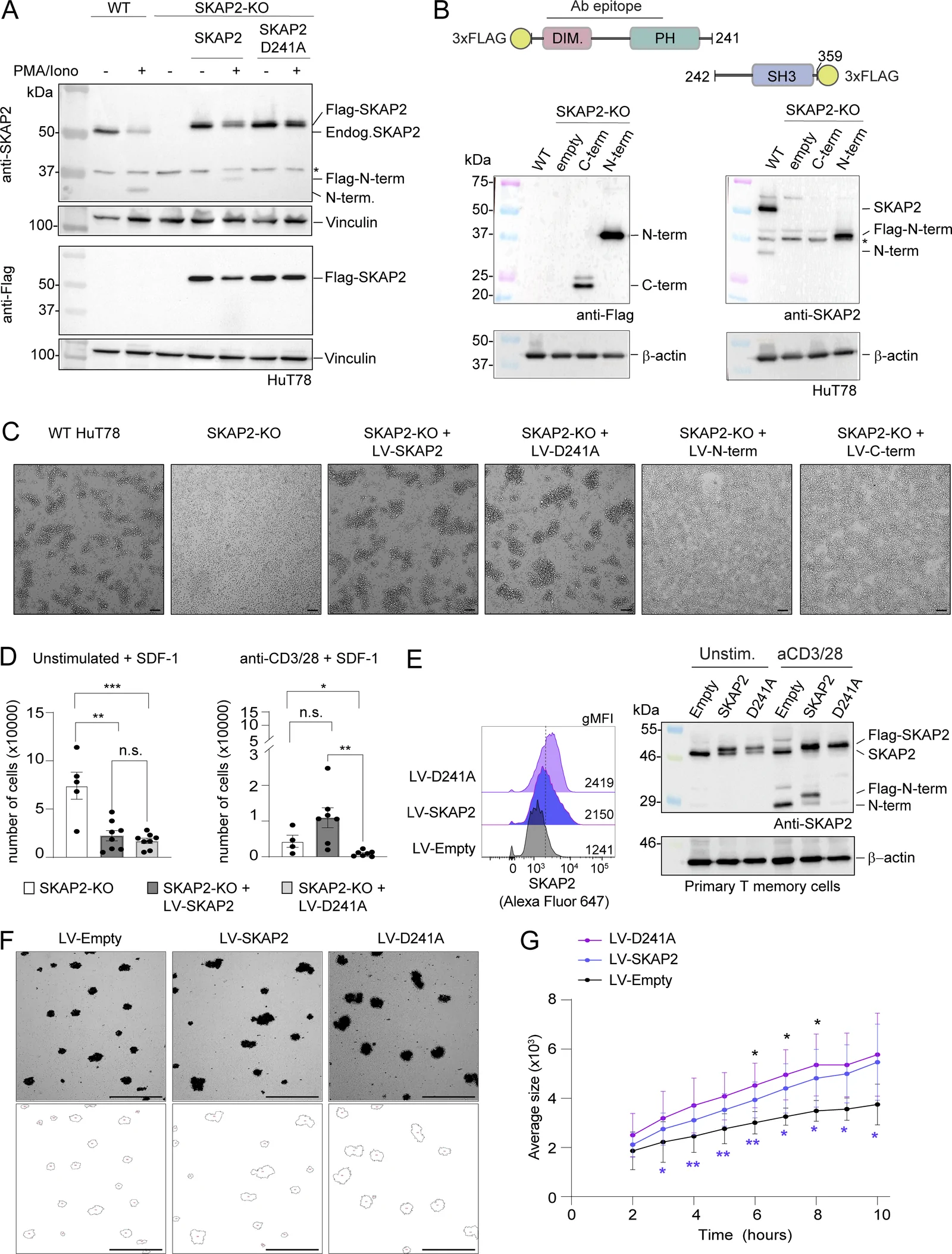
**SKAP2 cleavage relieves T cell adhesion. (A)** Immunoblot analysis showing the reconstitution of SKAP2 expression (either WT or D241A) in SKAP2-KO HuT78 cells. Cells were either left resting or stimulated with PMA and ionomycin for 24 h. Immunoblotting was performed using an anti-SKAP2 (top panel) or an anti-Flag (bottom panel) antibody. Vinculin expression was used as a loading control. The asterisk (*) indicates a nonspecific band. Representative of at least *n* = 3 independent experiments. **(B)** Top: Schematic representation of the two SKAP2 fragments generated by proteolytic cleavage. Fragments were tagged with a 3×-Flag at either the N or C terminus for LV transduction of HuT78 SKAP2-KO cells. Bottom: Immunoblots showing the expression of both fragments in reconstituted SKAP2-KO HuT78 cells. Immunoblot analysis was performed using anti-Flag (left panel) and anti-SKAP2 (right panel) antibodies. β-actin expression was used as a loading control. The asterisk (*) indicates a nonspecific band. Representative of *n* = 4 independent experiments. **(C)** Representative phase-contrast images of SKAP2-KO HuT78 cells reconstituted to express wild-type SKAP2 (LV-SKAP2), the D241A mutant (LV-D241A), and the two SKAP2 fragments generated by caspase cleavage (LV-N-term and LV-C-term). Images were acquired with an ImageXpress Micro 4 using a 10× objective. Scale bar: 100 µm. Representative of at least *n* = 4 independent experiments. **(D)** Migration assay of SKAP2-KO HuT78 T cells reconstituted to express wild-type SKAP2 or the uncleavable D241A mutant. Cells were either left resting or were stimulated for 24 h with aCD3/aCD28-coated beads. Then, cells were left to migrate toward a gradient of SDF-1 for 4 h. Each dot represents one individual clone or clone pool. *n* = 3–4 independent experiments. Mean ± SEM, unpaired *t* test, two-tailed. From left to right: **P = 0.0019, ***P = 0.0004, n.s. P = 0.3817, *P = 0.0257, n.s. P = 0.1324, and **P = 0.0039. **(E)** SKAP2 expression in primary T cells transduced with WT or D241A N-term-Flag-SKAP2, as measured by intracellular staining (left*, n* = 8 donors) and immunoblot (right, *n* = 2 experiments) using an anti-SKAP2 antibody. **(F)** Cell clustering analysis in primary CD4^+^ T lymphocytes transduced with WT or D241A SKAP2. Cells were stimulated for 10 h with anti-CD3 and anti-CD28–coated beads. Images were acquired hourly using a 10× objective with ImageXpress Micro 4. Cluster size was analyzed over time as indicated. Representative brightfield images (top panels) and the corresponding outlines of cell clusters identified by the macro at 8 h after stimulation (bottom panels) and used for quantification are shown. Data represent *n* = 5 independent donors. Scale bar: 500 µm. **(G)** Graph showing the average size of primary T cell clusters over time in experiments as in F. The average size was calculated as the total area occupied by the particles divided by the number of particles at each time point. Data represent *n* = 5 independent donors. Results are expressed as mean ± SD with multiple paired *t* test comparisons. Lower blue asterisks indicate significance of the WT SKAP2 samples compared with the empty vector controls (from left to right: *P = 0.0275, **P = 0.0041, **P = 0.0056, **P = 0.0093, *P = 0.0307, *P = 0.0400, *P = 0.0186, and *P = 0.0288), while upper P values are relative to the statistical difference between WT and D241A SKAP2-expressing samples (from left to right: *P = 0.0129, *P = 0.0126, and *P = 0.0359). Source data are available for this figure: SourceData F7.

To determine the functional effects of SKAP2 cleavage on T cell adhesion, we reconstituted SKAP2-KO HuT78 T cells with either WT or uncleavable SKAP2 carrying the D241A mutation (Fig. 7 A). We also generated constructs expressing the isolated N- and C-terminal SKAP2 fragments corresponding to those produced by cleavage at D241 and used them to reconstitute SKAP2-KO cells (Fig. 7 B). Lack of endogenous SKAP1 expression in this cell line allowed us to explore the functional effects of SKAP2 while eliminating the confounding effects of SKAP1 presence. Homotypic cell–cell adhesion was virtually completely lost in SKAP2-KO cells, while re-expression of both WT and uncleavable SKAP2 restored it (Fig. 7 C). Reconstitution of SKAP2-KO cells with either the N- or the C-terminal fragment of SKAP2 did not rescue cell clustering (Fig. 7 C), indicating that the full-length protein is required for this effect and suggesting that its cleavage may cause the termination of its adhesion-promoting activity.

To assess whether SKAP2 cleavage influences T cell migration following stimulation, we performed transwell migration assays using HuT78 T cells that were either SKAP2-KO or reconstituted with WT SKAP2 or the uncleavable mutant D241A. Consistent with data from primary T cells (Fig. 3, F and G; and Fig. S4), in the absence of TCR stimulation, SKAP2-KO cells exhibited significantly enhanced chemokine-induced migration compared with SKAP2-expressing cells, suggesting that reduced inter-cell adhesion in SKAP2-KO cells facilitates their migration (Fig. 7 D). Both WT and SKAP2 D241A-expressing cells showed similar levels of migration, consistent with the fact that SKAP2 is not cleaved in unstimulated cells. However, following 24 h of stimulation with aCD3/aCD28 to induce SKAP2 cleavage, both SKAP2-KO cells and cells reconstituted with WT SKAP2 retained their ability to migrate toward the SDF-1 gradient. In contrast, cells expressing the uncleavable SKAP2 D241A mutant completely lost their migration capacity (Fig. 7 D). These findings indicate that while SKAP2 expression is necessary for cell adhesion, it hinders cell migration, consistent with our previous findings. Following stimulation, SKAP2 cleavage emerges therefore as a switch contributing to a state permissive for T cell migration.

To determine the impact of SKAP2 cleavage on adhesion, primary T cells were transduced to overexpress WT or D241A SKAP2 (Fig. 7 E), followed by single-cell resuspension, stimulation with aCD3/aCD28 coated beads and imaging over time to assess cluster formation upon homotypic adhesion. Using *n* = 5 independent donors, we found that overexpression of WT SKAP2 was sufficient to enhance clustering, consistent with our initial observations (Fig. 3 A). Notably, expression of the uncleavable SKAP2 D241A led to an additional and significant increase in the average size of the clusters (Fig. 7, F and G), further highlighting that full-length SKAP2 expression enhances cell adhesion.

Taken together, these data indicate that SKAP2 expression is required for T cell adhesion, while its cleavage following T cell stimulation relieves it, thus fully enabling T cell migration.

## Discussion

In this study, building on the identification of common genetic polymorphisms previously linked to autoimmune disease risk (IBD and specifically CD), we characterized a thus far overlooked player and mechanism of stimulus-dependent regulation of T cell functions. Specifically, we show that genetically determined levels of SKAP2 expression control activation-induced T lymphocyte adhesion and migration. Consistent with their overall limited homology, SKAP2 acts in a nonredundant manner with SKAP1, and these two adapter proteins diverge in terms of regulation, protein interactomes and functions.

Our findings can now be reconciled with previous data on the role of SKAP1 in T lymphocyte function within a coherent mechanistic framework. In the very early phases (minutes) of T cell activation, abundant SKAP1 levels are required for LFA-1 clustering and the initial formation of the T cell–APC conjugate (Wang et al., 2003), suggesting the possibility that SKAP1 might be required to induce such initial transient adhesion, while being insufficient to sustain T cell adhesion during activation. In fact, while SKAP1 is constitutively expressed and its levels do not change upon TCR engagement, SKAP2 is inducible. Hence, a coherent picture emerges in which SKAP1 is required for early events during T cell activation, such as the initial interaction with an APC. Upon productive T cell activation and upregulation, SKAP2 contributes to restraining T cell migration by increasing adhesion. Finally, SKAP2 proteolytic cleavage at a later stage enables T cell migration (model in Fig. S5 I). Although still in part speculative, this model is also consistent with the observation that T cell priming by dendritic cells requires three phases: the first one, lasting ∼8 h, is characterized by multiple transient encounters. Over time, T cells progressively decrease their motility. In the following ∼12 h, the second phase is characterized instead by stable contacts, followed by a third phase on day 2 that features reacquisition of high motility and proliferation (Mempel et al., 2004). It would be tempting to speculate that in T cells, the initial SKAP1-dependent, transient integrin activation is rapidly reversed in the absence of SKAP2 induction, leading to dissociation of T cells from APCs or endothelial cells.

The lack of redundancy between SKAP1 and SKAP2 in the control of T cell responses is somewhat surprising given their ability to efficiently interact with the same TCR-proximal adapter, FYB (Griffiths et al., 2001; Liu et al., 1998; Timms et al., 1999; Voisinne et al., 2019). One crucial aspect in this regard is the fact that the two proteins are differentially regulated. The distinct functional roles of SKAP1 and SKAP2 can therefore be partly linked to their temporal regulation in T cells. Additionally, our proteomics data indicate the existence of distinct interactomes involving SKAP1 and SKAP2. On the one hand, SKAP2 is constitutively present together with SKAP1 in a trimolecular complex with FYB. Since SKAP2 possesses an N-terminal dimerization domain, it is tempting to speculate that the private SKAP2 interactome requires SKAP2 homodimerization enabled by its increased expression levels. Interestingly, a SKAP1 isoform lacking a single amino acid (V293) in a small motif required for the interaction with FYB was not co-immunoprecipitated with SKAP2 and instead appeared to account for a small SKAP1 private interactome. What is the functional relevance of this specific additional variant remains to be understood. Finally, we show that post-activation caspase-mediated cleavage of SKAP2 resulted in the release of interacting proteins and relieved its positive effect on T lymphocyte adhesion, enabling migration of activated T cells. Hence, caspase-mediated cleavage of SKAP2 has a specific role in the temporal progression of T cell responses.

Our study characterized a mechanism central to the regulation of TCR-induced human T cell adhesion and migration. How can increased SKAP2 expression lead to an increased risk of CD and other autoimmune disorders? While still speculative, we envision a scenario in which genetic variation at the *SKAP2* locus may enhance adhesion and tissue retention of activated and potentially pathogenic T cells, thereby promoting their abnormal compartmentalization and/or tissue accumulation (Fig. S5 J). By promoting an activation-induced adhesive state, genotype-driven increased SKAP2 expression may alter the normal recirculation of activated T cells and favor their persistence within disease-relevant immune compartments. If this turns out to be accurate using in vivo models, then levels of SKAP2 expression may also potentially define a mechanistically distinct subgroup of CD patients who may successfully respond to anti-integrin therapy. Indeed, given the increasing role of integrin antagonists in the therapeutic arsenal for IBD (Cherry et al., 2015), it will be important to determine if prior knowledge of the genetic status of the *SKAP2* locus can be exploited to select patients who, because of high SKAP2 levels in T lymphocytes, may be particularly susceptible to the therapeutic effects of such inhibitors. One possibility is that homozygosity for the minor, alternative *SKAP2* allelic variant would amplify adhesion-dependent programs and promote tissue retention of T lymphocytes that can potentially be reversed through the inhibitory effects of antagonists of α4β7 integrins, which are required for gut homing. Information regarding the genotype at the *SKAP2* locus may thus assist in the therapeutic stratification of IBD patients. Retrospective and prospective studies exploring this possibility in conjunction with immunophenotyping of patients’ tissue samples are warranted.

Overall, the impact of our work is several-fold. First, using GWAS data for autoimmune diseases as a starting point, our study identified a previously unknown component of the machinery linking TCR signal transduction to the control of integrin activity. While previous work demonstrated the role of SKAP1 in the regulation of early inside-out integrin events (Raab et al., 2010), no function has been assigned to SKAP2 in T cells. In this regard, it is important to stress that to date, GWAS studies have not reported *SKAP1* as a risk locus for autoimmunity. Second, whereas the involvement of caspases in the control of T lymphocyte activation has been known for a long time (Newton and Strasser, 2003), our data shed light on a mechanism consisting of the termination of the communication between the TCR and the integrin system, which we propose may act as a switch enabling activated T cells to be relieved from strong adhesive interactions that would hinder their ability to migrate away from their site of activation. Finally, our study establishes a link between a previously identified autoimmune disease risk locus and a specific gene, a cell type, and a mechanism of action, a task whose notorious complexity hinders the understanding and the clinical translation of the vast majority of the findings reported in GWAS studies. Future studies will be needed to determine the practical relevance of our findings for IBD, and specifically CD patients.

A limitation of this study is that due to their high number and contiguity, the specific SNP(s) responsible for the increased *SKAP2* expression observed in individuals homozygous for the minor allele could not be identified, nor could the precise molecular mechanism underlying this regulatory effect be defined. The *SKAP2* locus spans ∼0.6 Mbp and contains 194 noncoding variants in strong linkage disequilibrium, forming a large haplotype block. As a consequence, the association signal cannot be resolved to individual SNPs by statistical fine-mapping, and these variants are largely inherited together in the human population. Consistent with this genetic architecture, our experimental analyses relied on a representative SNP to stratify donors by genotype. In addition, the relatively low frequency of the minor allele, and the resulting small proportion of homozygous individuals, further limits the power to genetically disentangle candidate causal regulatory variants within this locus. A second limitation is that the study does not address dynamic SKAP2-dependent adhesive interactions in vivo, which will require the generation of suitable animal models, as well as the initiation of human studies in large cohorts of IBD patients.

## Materials and methods

### Isolation and culture of primary human cells and cell lines

Primary human T cells were separated from peripheral blood from healthy donors provided by the Swiss Blood Donation Centers of Basel and Lugano (Switzerland). Informed consent was obtained from all donors (authorization number CE 3428 from the Comitato Etico Canton Ticino). Peripheral mononuclear blood cells (PBMCs) were separated by gradient centrifugation (Ficoll-Paque Plus; GE Healthcare), followed by positive selection of CD14^+^ monocytes, CD4^+^ T cells, and CD8^+^ T cells using magnetic microbeads (Miltenyi Biotec). CD4^+^ T cell subsets were then sorted using a SORP FACSymphony S6 (BD Bioscience) based on the expression of the following surface markers: naïve T cells: CD4^+^CD25^–^CD45RA^+^CCR7^+^; memory T cells: CD4^+^CD25^–^CD45RA^–^CCR7^+/−^. CD8^+^ T cells were sorted as CD8^+^CD45RA^–^CCR7^+/−^. When necessary, sorted T cell subsets were cultured in RPMI-1640 medium supplemented with 5% human serum, 1% nonessential amino acids, 1% sodium pyruvate, 1% glutamine, penicillin, streptomycin, and 50 μM β-mercaptoethanol (complete medium). The human T cell leukemia line DND-41 (DSMZ, ACC 525) and the human T cell lymphoma line HuT78 (ATCC, TIB-161) were cultured in RPMI-1640 medium supplemented with 10% (FBS (GIBCO), 1% penicillin-streptomycin, and 1% L-GlutaMAX. The human Jurkat T lymphoblastoid cell line (ATCC, TIB-152) was cultured in RPMI-1640 medium supplemented with 10% FBS, 1% penicillin-streptomycin, 1% L-GlutaMAX, sodium pyruvate (1 mM), and 4-(2-hydroxyethyl)-1-piperazineethanesulfonic acid (HEPES, 10 mM). All primary cells and cell lines were maintained in a 5% CO_2_ humidified incubator at 37°C.

### T cell activation

Primary human T cells were activated, as previously described (Emming et al., 2020), using 96-well NUNC plates (Thermo Fisher Scientific) coated with anti-CD3 antibody (0.7 μg/ml; clone TR66, recombinant, produced in-house) and anti-CD28 antibody (1 μg/ml, BD Pharmingen). After 48 h, cells were removed from the stimuli and further expanded for up to 25 days. For day 40 cultures, cells were reactivated at day 20 as above. Recombinant human interleukin-2 (rhIL-2) (50 U/ml, produced in-house) was added to the cultures starting on day 5. Human T cell lines were stimulated for 24 h with 20 ng/ml PMA and 2 µg/ml ionomycin or using the Dynabeads T Cell Activation kit (Thermo Fisher Scientific), using 25 μl of Dynabeads for 1 × 10^6^ cells.

### Caspase inhibitors

To inhibit caspase activity, human T cell lines were incubated for 24 h with 100 μM z-VAD-FMK or 50 μM Z-DEVD-FMK, or 100 μM Ac-DEVD-CHO (all from Selleckchem) in the presence of 20 ng/ml PMA and 2 µg/ml ionomycin.

### Western blots

For western blot analysis, cells were lysed in RIPA lysis buffer (10 mM Tris-HCl, pH 8, 140 mM NaCl, 1% Triton, 1 mM EDTA, pH 8, 0.5 mM EGTA, pH 8, 0.1 SDS, and 0.1% N-deoxycholate). Subsequently, detergent-insoluble material was removed by centrifugation at 13,000 × *g* for 10 min at 4°C, and the supernatant was quantified using the DC Protein Assay (Bio-Rad Laboratories). 50 μg of lysate was used for sodium dodecyl sulfate–polyacrylamide gel electrophoresis (SDS-PAGE). The gel was transferred to a nitrocellulose or polyvinylidene fluoride membrane and blocked with 5% BSA or 5% milk in 1× Tris-buffered saline and 0.1% Tween 20 detergent. The primary antibodies used are listed in Table S1 and included: anti-SKAP2 (Sigma-Aldrich), anti-SKAP1 (Santa Cruz Biotechnology), anti-FYB (BD Transduction Laboratories), anti-HaloTag (Promega), anti-FLAG M2 (Sigma-Aldrich), anti-β-actin (Bio-Rad Laboratories), anti-GAPDH (Abcam), and anti-vinculin (Santa Cruz Biotechnology). Immune complexes were visualized by chemiluminescence using the ECL western blotting detection reagents (Bio-Rad Laboratories). Images were captured digitally on a ChemiDoc Imaging System (Bio-Rad Laboratories) or a Vilber Fusion FX blot imager.

### Genotyping

Genomic DNA (gDNA) was extracted from human PBMCs using the DNeasy Blood & Tissue Kit (Qiagen). Purified gDNA (11 ng) was used as a template for a TaqMan SNP Genotyping Assay (Thermo Fisher Scientific), targeting the human SNP rs17323934. The assay was performed using the ABI 7900HT Fast Real-Time PCR system (Applied Biosystems) or QuantStudio 3 Real-Time PCR Instrument (Thermo Fisher Scientific). Additional SNPs located in the *SKAP2* locus were amplified in separate PCR reactions using the Platinum SuperFi II DNA Polymerase (Thermo Fisher Scientific) proofreading polymerase, followed by Sanger sequencing (performed at Microsynth) with the primers listed in Table S1.

### Surface and intracellular staining

Before all surface and intracellular stainings, cells were stained with LIVE/DEAD Fixable Blue Dead Cell Stain (Invitrogen) to exclude non-viable cells. For intracellular staining of SKAP1 or SKAP2, cells were fixed and permeabilized using the IntraSure kit (BD Biosciences) and incubated with anti-SKAP1 (0.625 μg/ml, Santa Cruz Biotechnology) or anti-SKAP2 antibody (1.25 μg/ml, Sigma-Aldrich). SKAP1 and SKAP2 were detected using Alexa Fluor 647–conjugated anti-mouse or anti-rabbit IgG (H+L) (Sigma-Aldrich), respectively. For dual SKAP1/SKAP2 staining, SKAP1 was detected using goat anti-mouse IgG1–FITC (Southern Biotech). The surface staining for CD25 was performed in MACS buffer using the conjugated antibody specified in Table S1. Human monocytes were stained with an anti-CD14-FITC antibody. Samples were acquired on a FACSCelesta Cell Analyzer, a FACSymphony A5, or a Canto 2 (all BD Biosciences), and data were analyzed using the FlowJo Software.

### Gene deletion by CRISPR-Cas9

Gene editing with CRISPR-Cas9 for functional analyses in cells of the immune system was notified to the Federal Coordination Centre for Biotechnology (authorization number A202879). Gene deletion was performed as previously described (Bianchi et al., 2026; Emming et al., 2020). Briefly, to generate SKAP2-KO clones, 5 × 10^5^ HuT78 cells were electroporated with 12.5 µg Truecut Cas9 protein (Thermo Fisher Scientific) along with crRNA:tracrRNA hybrids (75 pmol) using the Neon Transfection System (Thermo Fisher Scientific). All sgRNAs sequences, targeting the first exon of the *SKAP2* gene, are reported in Table S1. 48 h after electroporation, 4,000 cells were serially diluted into a 96-well plate starting from a concentration of 2 × 10^4^ cells/ml. Single colonies were then picked and expanded. Effective SKAP2 deletion was verified by western blot. For experiments using bulk KO populations, SKAP1, SKAP2, or SKAP1/SKAP2 DKO memory CD4^+^ T cells were generated using the Amaxa 4D-Nucleofector system (Lonza). Briefly, 1.5 × 10^6^ T cells were nucleofected 2 days after stimulation with aCD3/aCD28. Cells were transfected with 10 μg of TrueCut Cas9 Protein version 2 (Thermo Fisher Scientific) and 160 pmol of crRNA:tracrRNA, supplemented with polyglutamic acid (PGA; Sigma-Aldrich) at a gRNA:PGA ratio of 1:0.8, in Lonza P3 buffer containing Alt-R Cas9 Electroporation Enhancer (10.8 μM; IDT). Electroporation was performed using the EH-115 program. After nucleofection, cells were grown in antibiotic-free medium for 48 h and then maintained in medium supplemented with 50 U/ml rhIL-2.

### Gene activation by CRISPRa

For CRISPRa, 13 sgRNAs were designed to target the promoter region of the *SKAP2* gene. The location of the TSS was identified using the UCSC Genome Browser (https://genome.ucsc.edu). The sgRNAs were selected to target a genomic region (hg38, chr7:26864520–26865089), covering 70 nucleotides downstream and 500 nucleotides upstream of the TSS. NTC sequences were selected from Refs. (Doench et al., 2016; Shifrut et al., 2018). Complementary oligonucleotides containing either NTC sequences or sgRNAs targeting the *SKAP2* TSS, with BbsI recognition sites at the ends (Table S1), were annealed and cloned into the BbsI-digested pXPR_502 plasmid (#96923; Addgene; Sanson et al., 2018). All sgRNAs used in this study are provided in Table S1. The pZR112_Lenti-SFFV-mCherry-2A-dCas9-VP64 plasmid (#180263; Addgene; Schmidt et al., 2022) was modified by replacing the mCherry reporter with ZsGreen. After validating all plasmids by DNA sequencing, LV particles were produced for both the sgRNA plasmids and the modified dCas9-VP64 constructs. CD4^+^ T lymphocytes were activated using aCD3/aCD28 antibodies and transduced with the dCas9-VP64 lentivirus after 24 h. A second transduction with sgRNA lentiviruses, containing a puromycin-resistance gene, was performed on day 2. Puromycin selection (2 μg/ml) was performed for 48 h (day 5–7), followed by cell sorting to isolate ZsGreen^+^ cells. On day 16, cells were restimulated with plate-bound aCD3/aCD28 or aCD3/rICAM-1 for 5 h to assess SKAP2 expression by intracellular staining.

### Plasmid cloning and mutagenesis

The full-length human *SKAP2* cDNA was amplified from PBMCs and cloned in-frame with an N-terminal Flag-tag into the LV vector pScalps-Puro (#99636; Addgene) (Montagner et al., 2016). In some cases, SKAP2 was N-terminally tagged with a HaloTag by PCR. The D241A and D71G mutations were introduced via site-directed mutagenesis using primers listed in Table S1. Briefly, 50 ng of pScalps Flag-SKAP2 plasmid was mutated by PCR using Pfu DNA polymerase and mutagenic primers. At the end of the amplification, the methylated parental DNA was digested with the DpnI enzyme in Buffer A (Roche) at 37°C for 2 h, followed by inactivation at 80°C for 20 min. The mutated plasmids were transformed into DH10B Competent Cells (Thermo Fisher Scientific). The HaloTag–SKAP2 D241A mutant was generated by site-directed mutagenesis using the Q5 Site-Directed Mutagenesis Kit (New England Biolabs) with primers listed in Table S1, introducing the D241A substitution and a silent PvuI site for colony screening, using the pScalps-Puro HaloTag–SKAP2 plasmid as a template. In some experiments, Flag-SKAP2 and Flag-SKAP2 D241A were subcloned into the pLVX-EF1α-IRES-ZsGreen1 vector (Clontech) using the XbaI and NotI restriction sites. The N-terminal (amino acids 1–241) and C-terminal (amino acids 242–359) fragments of SKAP2 were cloned into pScalps-Puro in-frame with a 3×Flag tag.

### 3D T cell migration and imaging analysis

Chemotaxis experiments were performed using μ-Slide Chemotaxis (Ibidi) chambers. Primary CD4^+^ T cells were enriched from PBMCs, activated with plate-bound aCD3/aCD28, and expression of either SKAP1, SKAP2, or both proteins was ablated by CRISPR-Cas9 RNP transfections as described above. Cells were cultured for a total of 7 days after activation and prior to the migration experiment. To measure migration, cells were embedded in 300 μl of bovine collagen type I (Ibidi) to a final concentration of 1.5 mg/ml. The collagen matrix was prepared according to the manufacturer’s instructions. Briefly, a collagen base solution was prepared by mixing 20 μl of 10× RPMI 1640 Medium (Sigma-Aldrich) with 11.3 μl of NaOH 1M, 5.3 μl of 7.5% (weight/volume) NaHCO_3_ and 50 μl of heat-inactivated FBS. The volume was adjusted to 137.5 μl with sterile nuclease-free water. Bovine collagen type I was diluted to 4 mg/ml in 0.1 M acetic acid, and 112.5 μl were added to the collagen base solution. Subsequently, 9 × 10^5^ T cells were resuspended in 50 μl of RPMI 1640 (Sigma-Aldrich) supplemented with 10% FBS and added to the collagen mix. For each sample, the collagen-cell suspension was prepared individually, and 6 μl of the mix (corresponding to 1.8 × 10^4^ cells) was pipetted into the central reservoir of each chemotaxis chamber. To induce homogeneous collagen polymerization, the mix was prepared on ice followed by slowly raising the temperature from 4 to 37°C within a 45 min timeframe, as previously described (Antonello et al., 2022). After polymerization, 65 μl of RPMI medium with 10% FBS were pipetted into both side reservoirs. Slides were then placed in a humidified Petri dish and left overnight in an incubator at 37°C with 5% CO_2_. The following day, 30 μl of SDF-1 (200 mM) was pipetted into one of the reservoirs, resulting in a final concentration of 100 mM. The slides were then subjected to time-lapse video microscopy (ImageXpress Molecular Devices) for 4 h, with 40-s time intervals between frames. For analysis, transmitted light images were collected and converted to pseudofluorescence using the WID-U plugin on FIJI/ImageJ (Antonello et al., 2023). Images were then batch-processed using TrackMate (Tinevez et al., 2017), employing the Laplacian of Gaussian detector to detect spots and the Sparse Linear Assignment Problem tracker to link spots into trajectories. Tracks shorter than 10 frames were excluded from further analysis. Positional variables of trajectories (TrackNumber, SliceNumber, X-Value, and Y-Value) were ordered according to temporal sequence. For each trajectory, the initial x- and y-coordinates were subtracted from all subsequent positions within the same track to normalize trajectories to the origin (0,0). Normalized coordinates were used to generate spider plots. Euclidean displacement from the origin at each time point was calculated as $d=\sqrt{x^{2}+y^{2}}$, where x and y represent the normalized coordinates at each frame. Downstream trajectory analyses and extraction of additional metrics, including rolling directionality and speed, were performed using CellTracksColab (Gómez-de-Mariscal et al., 2024), a Python-based open-source platform designed for quantitative cell trajectory analysis. Mean speed was calculated for each trajectory as the total distance travelled divided by the total track duration. Visualization was performed in R using the tidyverse and ggplot2 packages, and violin plots were generated to display the distribution of single-cell mean speed values for each condition, with the median indicated and the interquartile range shown as error bars. Statistical significance was assessed using permutation testing, based on random reassignment of group labels to generate null distributions of the corresponding test statistics. Permutation-based P values are discrete and depend on the number of randomizations performed. Correction for multiple comparisons was applied using the Bonferroni method.

### LV transduction

For virus generation, HEK-293T cells were transfected with packaging plasmid vectors psPAX2 and pMD2.G (#12260 and #12259; Addgene) and LV vector using the CaPO_4_ or polyethylenimine methods. After 48 h, the supernatant containing LV particles was filtered and concentrated using a 4× Lentivirus Concentrator Solution (PEG-8000 and NaCl at 40% wt/vol). Concentrated virus was subsequently added to a 6-well plate containing 2 × 10^6^ HuT78 cells and 8 μg/ml polybrene. Cells were spin-infected for 90 min at 800 × *g* and then incubated for 6 h at 37°C. After the incubation, the virus-containing medium was replaced with fresh medium. After 2 days, puromycin (1 μg/ml) was added for selection. Assays were carried out after 4 days of puromycin selection. For transduction of primary human T lymphocytes, 1.5 × 10^5^ cells were seeded into flat-bottom 96-well plates pre-coated with aCD3/aCD28 and transduced with 5 μl of concentrated lentivirus. rhIL-2 (50 U/ml) was added on day 5 of culture, and on day 7 (when a pLVX-EF1α-IRES-ZsGreen1 backbone plasmid was used), GFP^+^ cells were sorted using SORP FACSymphony S6 (BD Bioscience). All experiments were performed starting from day 16 following activation.

### Affinity purification and IP-MS

#### HaloTag affinity purification and MS

SKAP2-KO HuT78 cells were reconstituted with HaloTag-SKAP2, HaloTag-SKAP2-D241A, or HaloTag alone. Cells were either left resting or were stimulated for 20 h with 200 nM PMA and 1 µg/ml ionomycin (Thermo Fisher Scientific). Cells were lysed using a total cell lysis buffer containing 250 mM NaCl, 50 mM Tris-HCl (pH 8.0), 0.5 mM EDTA, 0.5 mM EGTA, and 0.2% NP-40, supplemented with 1 mM PMSF (Sigma-Aldrich), 1× protease inhibitor cocktail (Sigma-Aldrich), and 1× phosphatase inhibitor (Sigma-Aldrich). 25 μl of Halo-Trap Agarose beads (Chromotek) were added to 2 mg of total cell lysate and incubated in rotation end-over-end for 2 h at 4°C. Precipitated samples were washed with total cell lysis buffer for five times, followed by two washes with PBS. After washing, samples were processed directly for on-bead protein digestion for MS analysis exactly as previously described (Bianchi et al., 2026). Briefly, precipitated proteins on beads were resuspended in 8 M urea and 50 mM ammonium bicarbonate, followed by reduction with 10 mM dithiothreitol for 60 min at 37°C and alkylation with 50 mM iodoacetamide for 30 min at room temperature. Proteins were digested with 1 µg Lys C (Wako Fujifilm) for 2 h at 37°C, diluted to 2 M urea, and further digested overnight with 1 µg trypsin (Promega) at 37°C. Digestion was quenched by the addition of 2% acetonitrile and 0.3% trifluoroacetic acid. Peptides were purified using C18 StageTips (Rappsilber et al., 2007), dried by vacuum centrifugation, resuspended in 2% acetonitrile, 0.5% acetic acid, and 0.1% trifluoroacetic acid, and analyzed by single-shot LC–MS/MS. Peptides were separated on an EASY-nLC 1200 HPLC system (Thermo Fisher Scientific) coupled online via a nanoelectrospray source (Thermo Fisher Scientific) to a Q Exactive HF mass spectrometer (Thermo Fisher Scientific). Peptides were loaded in buffer A (0.1% formic acid) into a 75-µm inner diameter, 50-cm long column, packed in-house with ReproSil-Pur C18-AQ 1.9-µm resin (Dr. Maisch HPLC GmbH), and eluted over a 150-min linear gradient of 5–30% buffer B (80% acetonitrile, 0.1% formic acid) at a flow rate of 250 nl/min. The mass spectrometer was operated in a data-dependent (DDA) mode with a survey scan range of 300-1,650 m/z, resolution of 60,000 at 200 m/z, maximum injection time of 20 ms, and an AGC target of 3e6. The top 10 most abundant ions with charge 2–5 were isolated with a 1.8 m/z isolation window and fragmented by higher-energy collisional dissociation at a normalized collision energy of 27. MS/MS spectra were acquired with a resolution of 15,000 at 200 m/z, a maximum injection time of 55 ms, and an AGC target of 1e5. Dynamic exclusion was set to 30 s to reduce repeated sequencing.

#### IP-MS

For IP of SKAP2 and SKAP1 from primary T cells, human memory CD4^+^ T cells were stimulated with plate-bound aCD3/aCD28, followed by expansion for 12 days. After lysis using the same buffer as for HaloTag affinity purification, 1.5 mg of lysate was incubated for 3 h at 4°C with 4 µg of rabbit anti-SKAP1 (Proteintech), rabbit anti-SKAP2 (Proteintech), or rabbit IgG as control, followed by overnight incubation at 4°C with 50 μl of Protein G beads (Thermo Fisher). Immunocomplexes were washed five times with lysis buffer and twice with PBS, then processed for on-bead digestion and LC-MS/MS analysis as above. When co-IPs were analyzed by western blot, immunocomplexes were resuspended in 50 μl of Laemmli Sample Buffer (Bio-Rad), heated at 95°C for 5 min, and the beads were removed magnetically prior to western blot analysis.

### MS data analysis

The mass spectrometric raw data were analyzed with the MaxQuant software (version 2.4.2.0) (Cox and Mann, 2008), using the Andromeda search engine. Peptides were searched against the Human UniProt database (release February 2024) combined with a common contaminants library (247 entries). A false discovery rate of 1% for proteins and peptides was applied, and a minimum peptide length of 7 amino acids was required. Enzyme specificity was set to “Trypsin/P,” and a maximum of two missed cleavages were allowed. Carbamidomethylation of cysteine residues was specified as a fixed modification, while N-terminal protein acetylation and methionine oxidation were included as variable modifications. Label-free protein quantification (LFQ) was performed with the MaxLFQ algorithm (Cox et al., 2014), where a minimum peptide ratio count of 1 was required for quantification. The analysis of search results was performed with the Perseus software version 1.6.2.3 (Tyanova et al., 2016). The datasets were pre-processed by removing proteins identified only by site, reverse hits, and potential contaminants, and log_2_ transformation of LFQ intensities was applied. Biological replicates of each experimental condition were grouped, and proteins were filtered for a minimum of 3 (HaloTag affinity purification) or 4 (SKAP1 and SKAP2 IP) valid values in at least one group. For statistical analysis, missing data points were replaced by imputation from a normal distribution with 0.3 width and 1.8 downshift, and two-sided two-sample Student’s *t* tests or ANOVA multiple-sample tests were employed to identify significant changes in protein intensity between the experimental conditions.

### Bright-field imaging and analysis of cluster formation

Imaging of HuT78 cell lines was performed using the EVOS cell imaging system (Thermo Fisher Scientific) or the ImageXpress Micro 4 instrument. Cells were maintained in culture without fixation and visualized at 10× magnification. For cluster formation experiments in primary human T cells, cells were pipetted to achieve single-cell suspensions and stimulated for 10 h with anti-CD3 and anti-CD28–coated Dynabeads (Thermo Fisher Scientific) at a bead-to-cell ratio of 4:1. Images were captured every hour using a 10× objective on an ImageXpress Micro 4 instrument. Cell clustering over time was analyzed with an ImageJ macro, using size settings ranging from 500 pixels to ∞.

### Cell proliferation

To measure cell proliferation, 1 × 10^5^ primary T cells were restimulated with plate-bound aCD3/aCD28 in the presence of BrdU (3 μg/ml) for 24 h. After this time, cell viability was determined using Live/Dead staining (Invitrogen), and BrdU incorporation was analyzed using the BrdU Flow Kit (BD Biosciences) according to the manufacturer’s protocol. For intracellular staining, cells were incubated with anti-SKAP2 antibody (rabbit, 1.25 ng/μl, Sigma-Aldrich) and anti-BrdU-eFluor450 antibody (mouse, 1:100 dilution, Invitrogen) in 1× BD Perm/Wash buffer (BrdU Flow Kit, BD Biosciences) for 20 min at room temperature. After washing, cells were further incubated with anti-rabbit IgG (H+L) Alexa Fluor 647 (1:500 dilution, Sigma-Aldrich) in 1× BD Perm/Wash buffer for 30 min at 4°C. Following a washing step, the samples were analyzed on the FACSymphony A5 cytometer (BD Biosciences).

### Transwell migration assay with human T cells

Migration of HuT78 cells toward SDF-1 was measured using Transwell chambers (8-μm pore size; Corning Inc.). Briefly, 1.5 × 10^5^ cells were either left resting or were stimulated for 24 h with the Dynabeads T Cell Activation kit (Thermo Fisher Scientific) at a 1:1 cell-to-bead ratio. After bead removal, unstimulated and stimulated cells (100 μl) were transferred into the upper chamber of the Transwell system, while the lower chamber was filled with 600 μl of medium containing 50 ng/ml SDF-1 (Sino Biological). After 4 h of incubation at 37°C in a humidified atmosphere with 5% CO_2_, cells that migrated to the lower chamber were collected, and the medium volume was adjusted to 150 μl. The migrated cells were quantified by manual cell counting and by using a BD FACSymphony A5 flow cytometer, with acquisition performed at a medium flow rate with a fixed time of 110 s.

### Separation of murine CD4^+^ T cells and CRISPR-Cas9–mediated editing

CD4^+^ T cells were separated from spleen and lymph nodes of C57Bl/6J mice using magnetic CD4 (L3T4) microbeads (Miltenyi Biotec), followed by activation with plate-bound anti-CD3 (2 μg/ml; cl. 145-2C11, Thermo Fisher Scientific) and anti-CD28 (2 μg/ml; cl. 37.51, BioLegend) antibodies. Cells were collected for intracellular staining and/or protein extraction at different time points after activation. C57BL/6J mice were obtained from Charles River Italy. Organ collection was performed in accordance with Swiss Federal Veterinary Office guidelines and with approval from the Cantonal animal experimentation committee, Dipartimento della Sanità e della Socialità Cantone Ticino (authorizations numbers 31152–TI10/2019 and 38023–TI45/2025). For CRISPR-Cas9–mediated editing, 2 × 10^6^ murine CD4^+^ T cells were activated for 48 h with aCD3/aCD28, followed by nucleofection using the Amaxa 4D-Nucleofector system (Lonza). T cells were transfected with 10 μg of TrueCut Cas9 Protein version 2 (Thermo Fisher Scientific) and 160 pmol of crRNA:tracrRNA, supplemented with PGA (Sigma-Aldrich) at a gRNA:PGA ratio of 1:0.8, in Lonza P3 buffer containing Alt-R Cas9 Electroporation Enhancer (10.8 μM; IDT). Electroporation was performed using the program CM-137. After nucleofection, cells were grown in antibiotic-free medium for 48 h and then maintained in medium supplemented with 100 U/ml rhIL-2.

### Transwell migration assay with murine CD4^+^ T cells

Migration of murine CD4^+^ T cells was measured toward SDF-1, used as a chemoattractant, in Transwell chambers (3-μm pore size; Corning Inc.). Briefly, CD4^+^ T cells were isolated, activated, and transfected to ablate Skap2 expression as described above. 4 days after transfection, 150,000 T cells were resuspended in 80 μl chemotaxis buffer (RPMI-1640 supplemented with 10% heat-inactivated FBS (GIBCO), 1% penicillin/streptomycin, 1% GlutaMAX, 1% nonessential amino acids, 1% sodium pyruvate, and 50 µM β-mercaptoethanol) and seeded into the upper chamber. The lower chamber was filled with 235 μl of chemotaxis buffer supplemented with 25 nM SDF-1 (Sino Biological). After 3 h of incubation at 37°C in a humidified atmosphere with 5% CO_2_, cells that migrated to the lower chamber and cells that remained in the upper chamber were collected, and the medium volume was adjusted to 150 μl. Cells from both chambers were quantified using a BD FACSymphony A5 flow cytometer, with acquisition performed at a medium flow rate with a fixed time of 110 s. The frequency of migration was calculated as the number of cells migrated into the lower, SDF-1–containing chamber divided by the total cell number (upper + lower chamber) × 100.

### RNA-seq

#### CRISPRa experiment

One donor homozygous for the major allele was selected for a CRISPRa-RNAseq experiment. On day 16, cells were restimulated with plate-bound aCD3/aCD28 antibodies or aCD3/rICAM-1 for 5 h. Following stimulation, total RNA was extracted using the Zymo Direct-zol RNA Microprep Kit (Zymo Research) according to the manufacturer’s protocol. RNA quality was assessed using the High Sensitivity RNA ScreenTape Assay (Agilent) on the Agilent TapeStation system.

#### RNA-seq of donors with different genotypes

Total RNA was extracted from CD4^+^ and CD8^+^ memory T cells isolated from four donors homozygous for the major allele and four donors homozygous for the minor allele. Total RNA was extracted using TRIzol Reagent (Invitrogen) with glycogen added as a carrier to improve RNA recovery. DNA contamination was removed using the RNase-Free DNase Set (Qiagen), and RNA was precipitated with sodium acetate.

#### RNA-seq of SKAP1-KO, SKAP2-KO, and DKO T cells

On day 12, cells were restimulated with aCD3/aCD28 antibodies for 2 h or 24 h. At each time point, cells were lysed in TRI Reagent RT (Molecular Research Center, Inc.), and total RNA was extracted using the Zymo Direct-zol RNA Microprep Kit (Zymo Research) according to the manufacturer’s protocol. For all the experiments, RNA quality was evaluated using the High Sensitivity RNA ScreenTape Assay on the Agilent TapeStation system. For the CRISPRa experiment and for RNA-seq of donors with different genotypes, stranded RNA-seq libraries were prepared using the Illumina TruSeq Stranded Total RNA Library Prep Gold Kit, following the manufacturer’s protocol. For the RNA-seq of SKAP1-KO, SKAP2-KO, and DKO T cells, libraries were prepared using the Watchmaker mRNA library prep kit. The size distribution of all libraries was assessed using the Agilent TapeStation D5000 HS ScreenTape Assay, confirming an average fragment size of ∼300 bp. Library quantification was performed with the QuantiFluor dsDNA System (Promega). Normalized libraries were pooled and sequenced as 61-bp paired-end reads on the Illumina NovaSeq 6000 platform.

### RNA-seq analysis

The analysis of RNA-seq data were conducted using a custom, open-access Nextflow pipeline available at https://github.com/fgualdr/mmrnaseq. Briefly, FASTQ files were filtered and trimmed via FASTP (version 0.23.2). Reads were then aligned to the human reference genome (hg38, Genome Reference Consortium Build GRCh38: GCF_000001405.26) utilizing STAR (version 2.7.9a) with specific parameters: --alignEndsType EndToEnd, --quantMode TranscriptomeSAM, --twopassMode Basic, --outSAMtype BAM Unsorted, --readFilesCommand zcat, --runRNGseed 0, --outFilterMultimapNmax 100, --winAnchorMultimapNmax 100, --alignEndsProtrude 100 DiscordantPair, --outFilterScoreMinOverLread 0.4, --outFilterMatchNminOverLread 0.4, --alignSJDBoverhangMin 1, --outSAMattributes All, --quantTranscriptomeBan Singleend, and --outSAMstrandField intronMotif. Duplicate reads were identified and marked using PICARD (version 3.0.0). Multi-mapped reads were labeled with PICARD (version 3.0.0) and excluded from further analysis. Gene-level read counts were computed using the summarizeOverlaps function from the R/Bioconductor package GenomicAlignments (version 1.26.0) based on the UCSC RefSeq annotation (*Homo sapiens* GRCh38.109). To normalize the data, invariant genes across samples and experimental conditions were identified using the R package GeneralNormalizer (https://github.com/fgualdr/GeneralNormalizer). Differential gene expression analysis was performed using DESeq2(R/Bioconductor version 1.38.0; R version 4.2.2), with the default normalization disabled. Genes meeting the criteria of an absolute log_2_ fold change exceeding 1 and an adj. P value ≤0.01 were considered significantly differentially expressed. Dimensionality reduction techniques, including PCA conducted with the R package FactoMineR (version 2.11) and Uniform Manifold Approximation and Projection implemented via uwot (version 0.1.16), were applied to analyze unsupervised clustering of differentially expressed genes. Clusters were refined using k-nearest neighbor algorithms provided by the R package FNN (version 1.1.4.1) and further grouped with Louvain community detection via the igraph R library (version 1.4.2). Heatmaps were generated with the ComplexHeatmap package (version 2.14.0). Gene set enrichment was assessed through fast GSEA using the R package fgsea (version 1.24.0). Both raw and processed RNA-seq data have been deposited in the Gene Expression Omnibus (GEO) database under accession number GSE288974.

### Statistical analysis

Statistical analyses for RNA-seq, migration, and MS experiments are described in the corresponding Methods sections. All other statistical analyses were performed using paired or unpaired two-tailed Student’s *t* tests or ANOVA, as appropriate, using Prism (GraphPad Software). Details of sample sizes and statistical tests are indicated in the figure legends for each experiment. P < 0.05 was considered statistically significant.

### Online supplemental material

Fig. S1 provides additional information on the genetic variation in the *SKAP2* locus and the gating strategies for T cells. Fig. S2 shows additional results on the genotype-dependent SKAP1/SKAP2 expression in T cells and monocytes. Fig. S3 contains additional controls for the CRISPRa experiments and the expression of mouse Skap1 and Skap2 in activated mouse T cells. Fig. S4 shows additional controls regarding the impact of SKAP2 expression on migration in both human and mouse T cells. Fig. S5 contains results regarding SKAP2 expression and proteolytic cleavage in different T cell types and conditions and the overall model of results. Data S1 contains the list of SNPs in the *SKAP2* locus with ncER score >90. Data S2 provides the RNA-seq datasets of CRISPRa T cells and of T cells from donors homozygous for the reference or alternative *SKAP2* allele. Data S3 provides the RNA-seq datasets of T cells control, SKAP1-KO, SKAP2-KO, and DKO in different conditions of stimulation. Data S4 contains the results of IP-MS experiments for endogenous SKAP1 and SKAP2 in primary human T cells. Data S5 provides the results of the HaloTag affinity purification and MS of SKAP2-KO HuT78 cells, reconstituted with the indicated constructs. Table S1 provides the materials used in this study. Videos 1, 2, and 3 show an example of the migration and imaging experiments for control, SKAP1-KO, and SKAP2-KO cells, respectively.

## Supplementary Material

Table S1 shows materials used in this study.

Data S1 shows SNPs in the *SKAP2* locus with ncER score >90.

Data S2 shows RNA-seq of CRISPRa T cells and RNA-seq of donors homozygous for the alternative or reference *SKAP2* allele.

Data S3 shows RNA-seq of control, SKAP1-KO, SKAP2-KO, and DKO primary T cells, unstimulated and stimulated for 2 and 24 h with aCD3/aCD28.

Data S4 shows IP-MS of SKAP1 and SKAP2 (versus IgG control) in primary human T cells.

Data S5 shows HaloTag affinity purification and mass spectrometry of SKAP2-KO HuT78 cells, reconstituted with the indicated constructs.

SourceData F3 is the source file for Fig. 3.

SourceData F5 is the source file for Fig. 5.

SourceData F6 is the source file for Fig. 6.

SourceData F7 is the source file for Fig. 7.

SourceData FS3 is the source file for Fig. S3.

SourceData FS4 is the source file for Fig. S4.

SourceData FS5 is the source file for Fig. S5.

## Data Availability

All raw data are either contained within this manuscript or are deposited in GEO with accession number GSE288974. All unique reagents generated in this study are available from the lead contact with a completed materials transfer agreement. Requests for further information and resources should be directed to and will be fulfilled by the lead contact, Silvia Monticelli (silvia.monticelli@irb.usi.ch).
