# Supplementary material for: A genetically regulated SKAP2 switch modulates human T lymphocyte adhesion and migration
Source: J Exp Med. 2026 Oct 20;223(12):e20260507. doi: 10.1084/jem.20260507 (PMC13682646; doi:10.1084/jem.20260507)
Supplement: Supplementary file 1 [file jem_20260507_sourcedataf3.pdf]

D

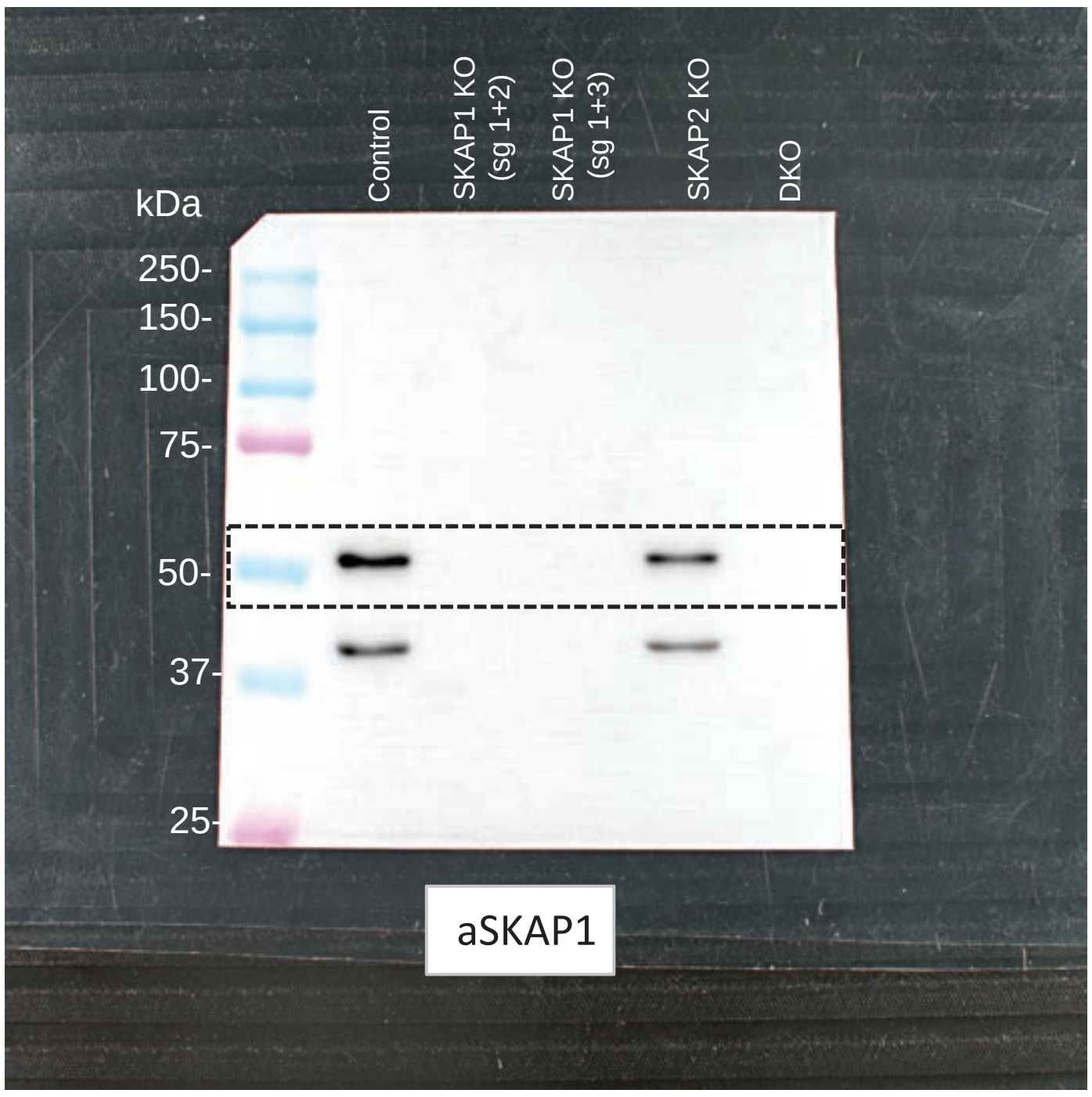
Control
SKAP1 KO (sg 1+2)
SKAP1 KO (sg 1+3)
SKAP2 KO
DKO
kDa
250-
150-
100-
75-
50-
37-
25-
aSKAP1

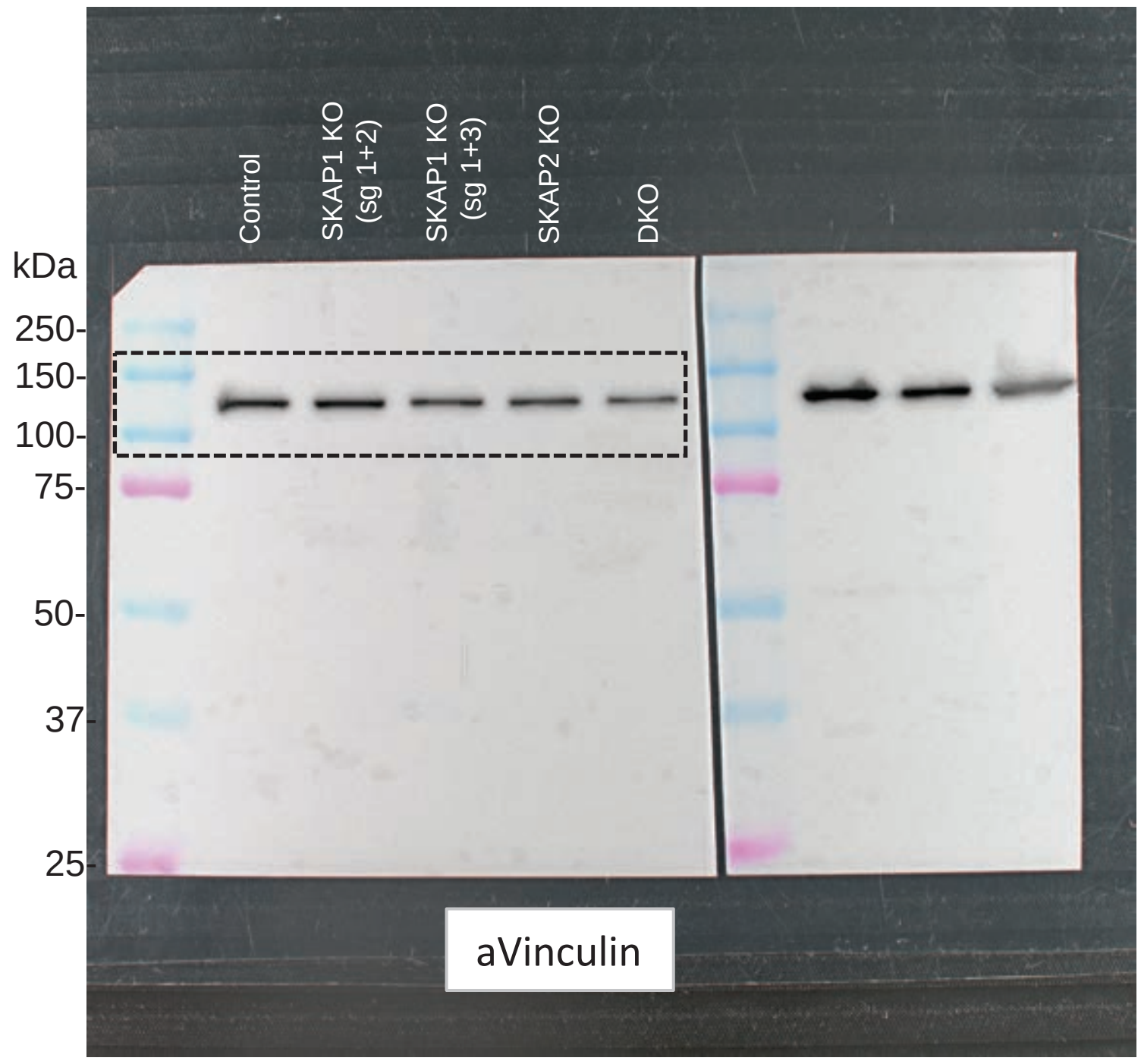
Control
SKAP1 KO (sg 1+2)
SKAP1 KO (sg 1+3)
SKAP2 KO
DKO
kDa
250-
150-
100-
75-
50-
37-
25-
aVinculin

E

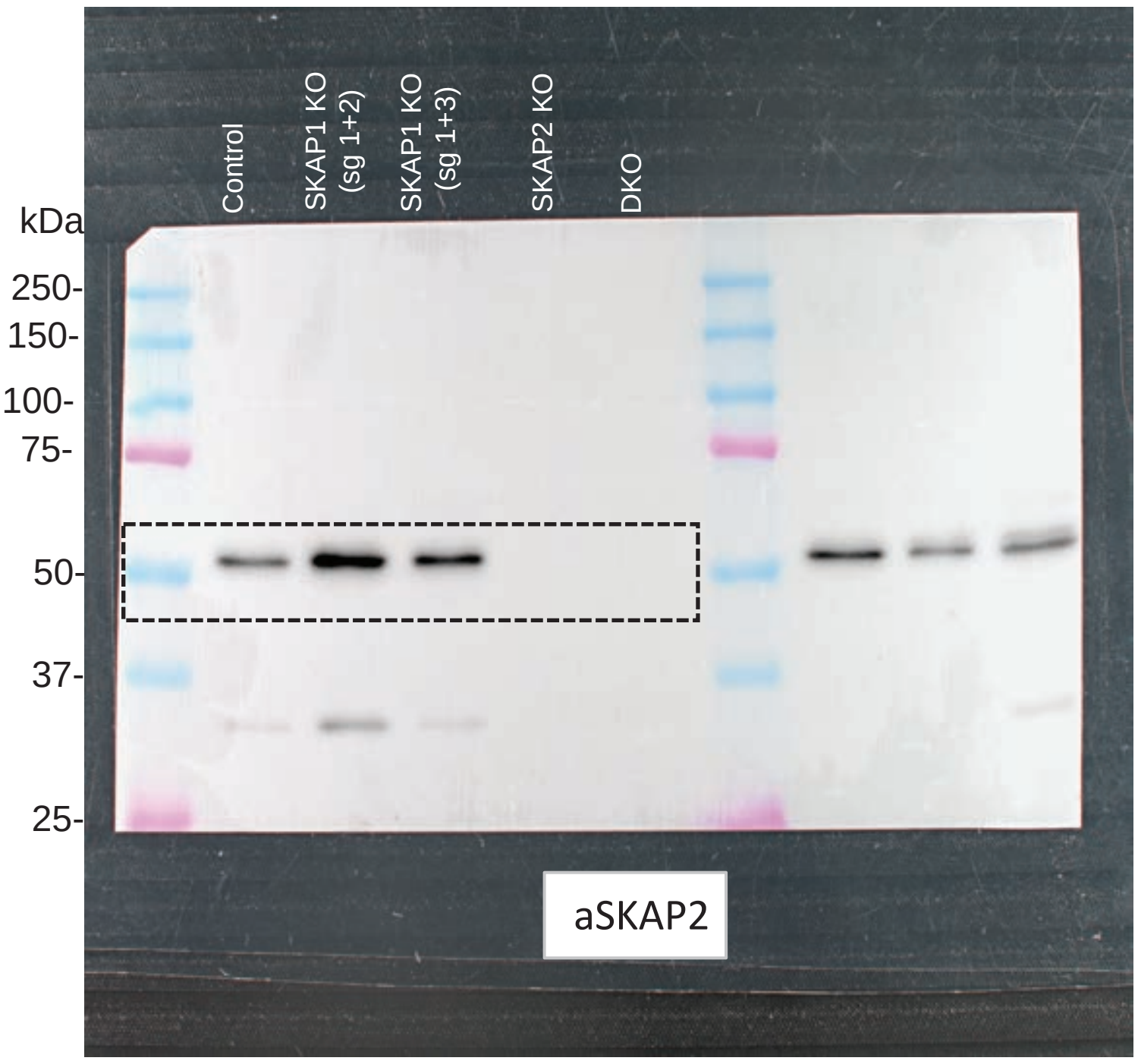
Control
SKAP1 KO (sg 1+2)
SKAP1 KO (sg 1+3)
SKAP2 KO
DKO
kDa
250-
150-
100-
75-
50-
37-
25-
aSKAP2

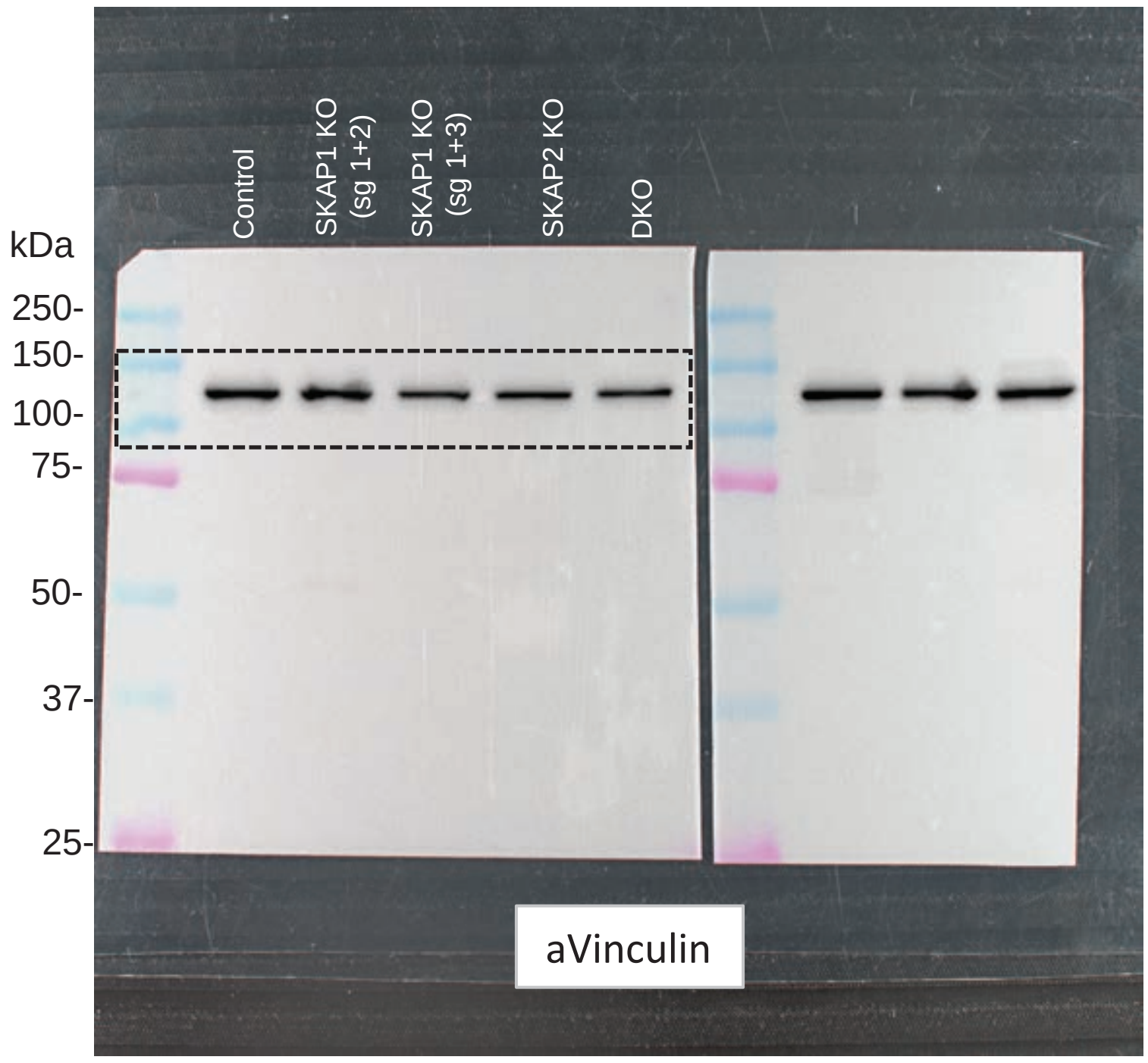
Control
SKAP1 KO (sg 1+2)
SKAP1 KO (sg 1+3)
SKAP2 KO
DKO
kDa
250-
150-
100-
75-
50-
37-
25-
aVinculin
