# Supplementary material for: A genetically regulated SKAP2 switch modulates human T lymphocyte adhesion and migration
Source: J Exp Med. 2026 Oct 20;223(12):e20260507. doi: 10.1084/jem.20260507 (PMC13682646; doi:10.1084/jem.20260507)
Supplement: Supplementary file 2 [file jem_20260507_sourcedataf5.pdf]

E

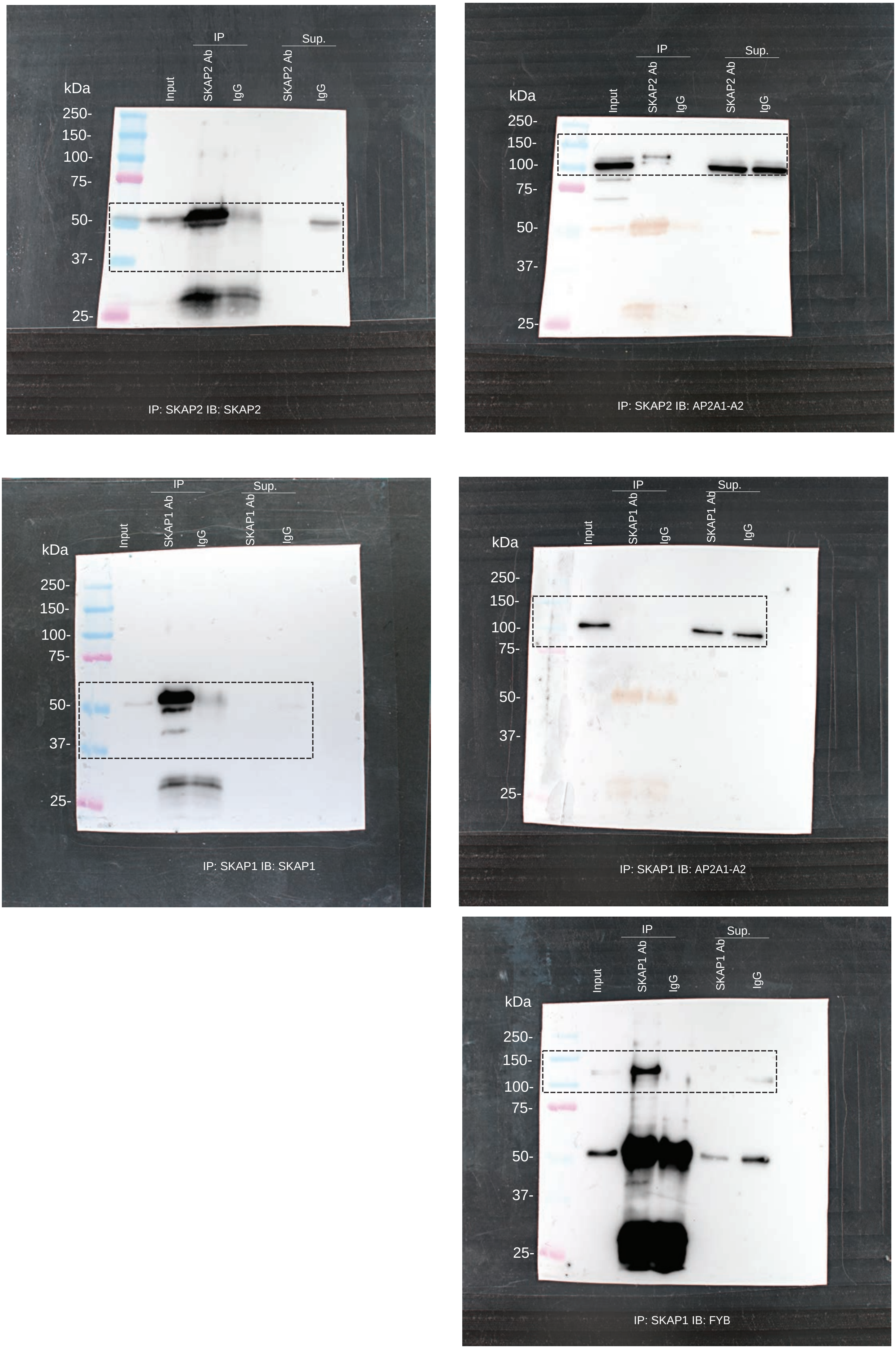

kDa
IP
Sup.
Input
SKAP2 Ab
IgG
SKAP2 Ab
IgG
250-
150-
100-
75-
50-
37-
25-
IP: SKAP2 IB: SKAP2
IP: SKAP2 IB: AP2A1-A2
SKAP1 Ab
IP: SKAP1 IB: SKAP1
IP: SKAP1 IB: AP2A1-A2
IP: SKAP1 IB: FYB
