# Supplementary material for: A genetically regulated SKAP2 switch modulates human T lymphocyte adhesion and migration
Source: J Exp Med. 2026 Oct 20;223(12):e20260507. doi: 10.1084/jem.20260507 (PMC13682646; doi:10.1084/jem.20260507)
Supplement: Supplementary file 3 [file jem_20260507_sourcedataf6.pdf]

A

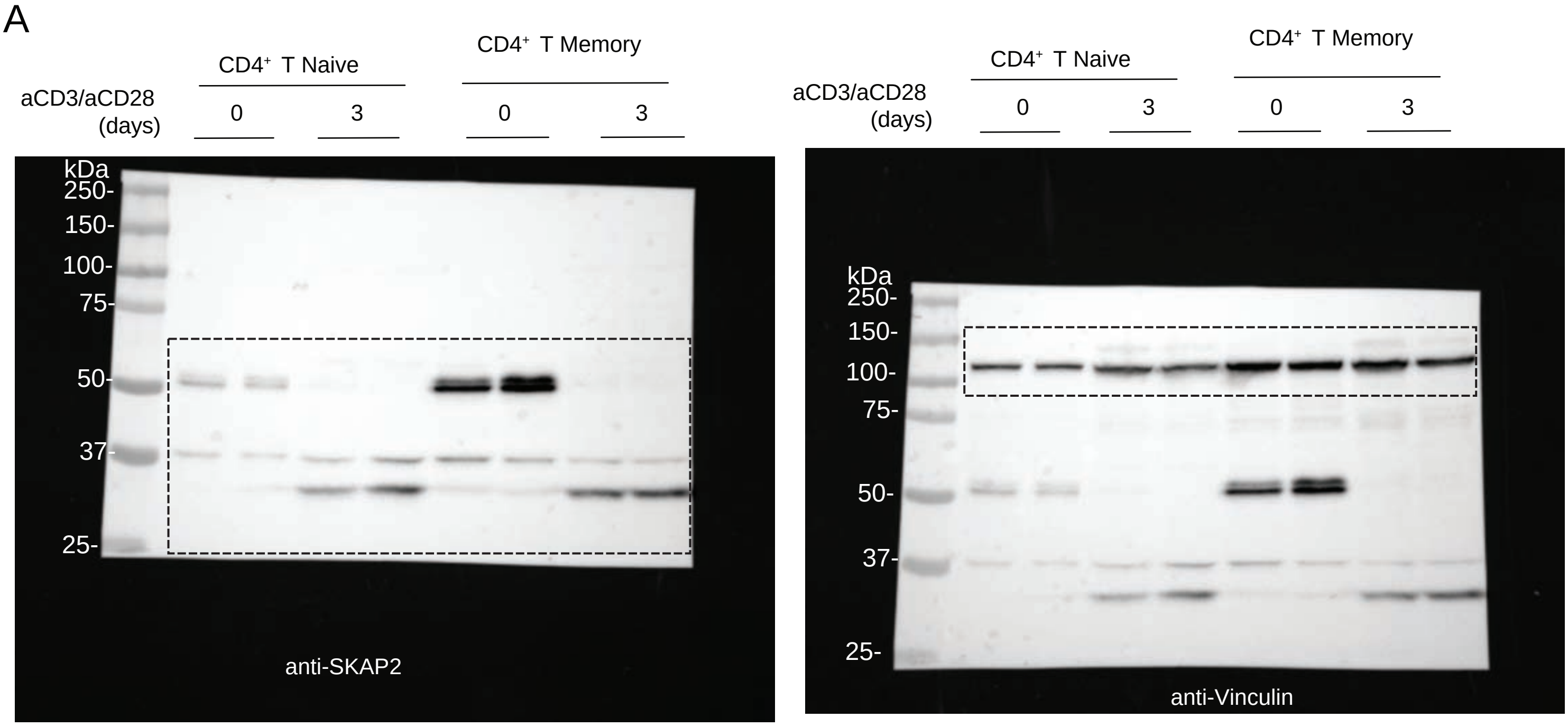

CD4⁺ T Naive
CD4⁺ T Memory
aCD3/aCD28 (days)
0 3 0 3
kDa
250-
150-
100-
75-
50-
37-
25-
anti-SKAP2
CD4⁺ T Naive
CD4⁺ T Memory
aCD3/aCD28 (days)
0 3 0 3
kDa
250-
150-
100-
75-
50-
37-
25-
anti-Vinculin

C

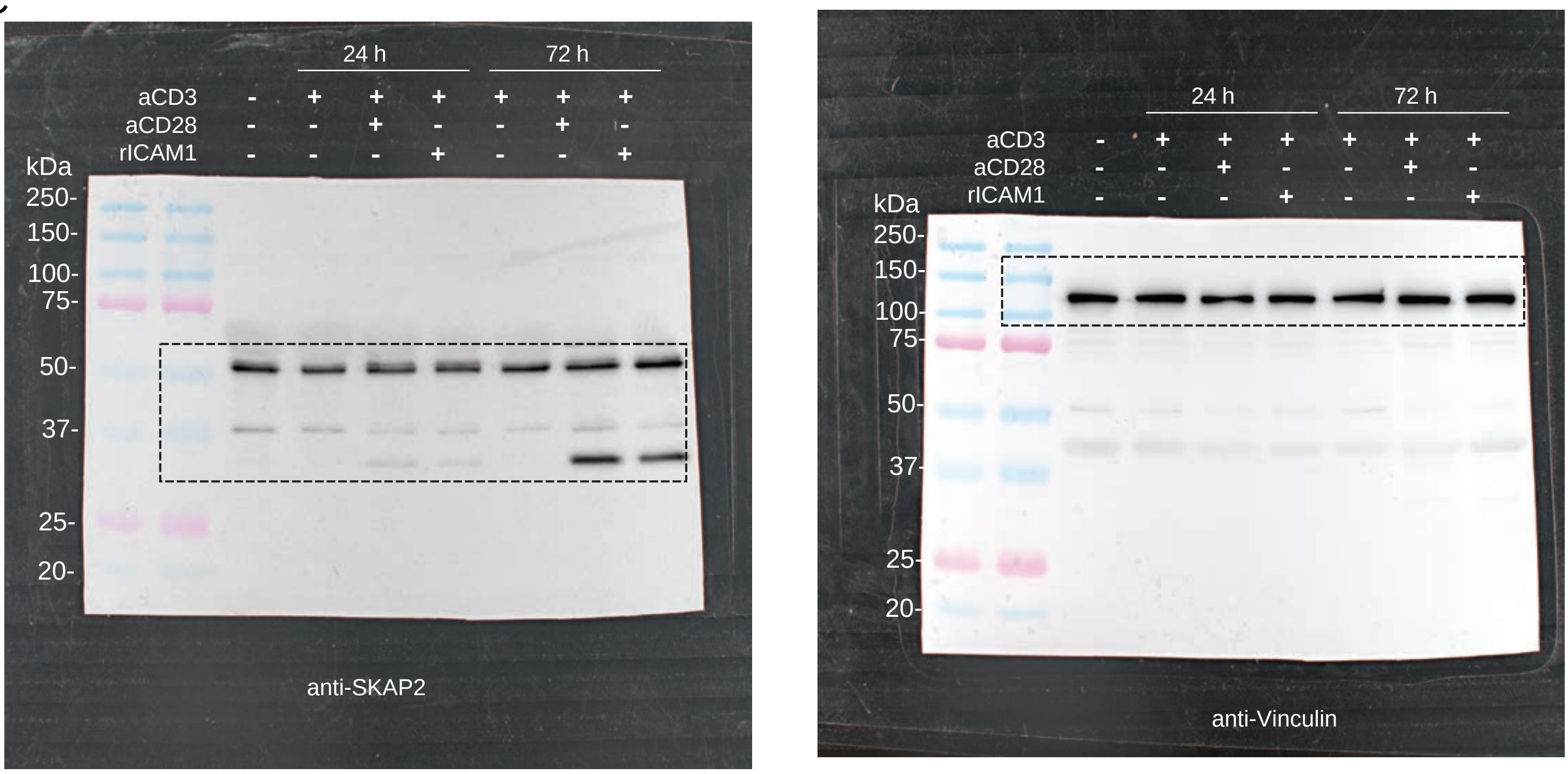

24 h
72 h
aCD3 - + + + + + +
aCD28 - - + - - + -
rICAM1 - - - + - - +
kDa
250-
150-
100-
75-
50-
37-
25-
20-
anti-SKAP2
24 h
72 h
aCD3 - + + + + + +
aCD28 - - + - - + -
rICAM1 - - - + - - +
kDa
250-
150-
100-
75-
50-
37-
25-
20-
anti-Vinculin

D

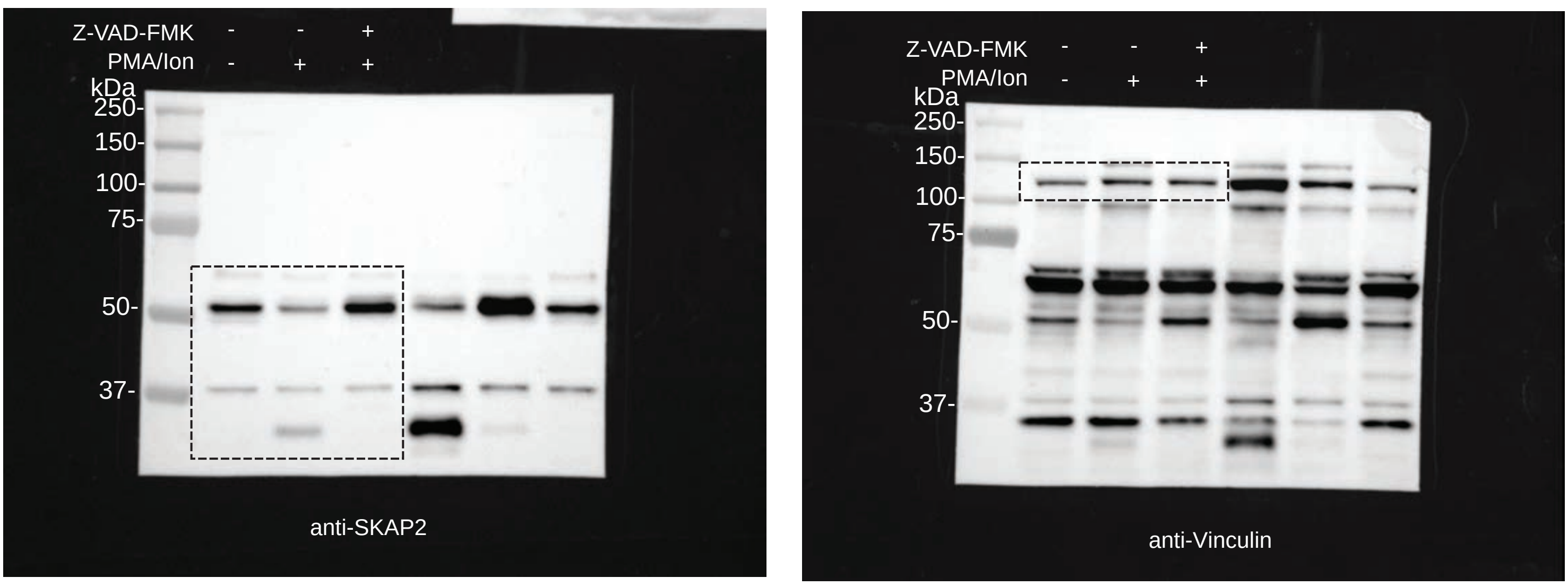

Z-VAD-FMK - - +
PMA/Ion - + +
kDa
250-
150-
100-
75-
50-
37-
anti-SKAP2
Z-VAD-FMK - - +
PMA/Ion - + +
kDa
250-
150-
100-
75-
50-
37-
anti-Vinculin

E

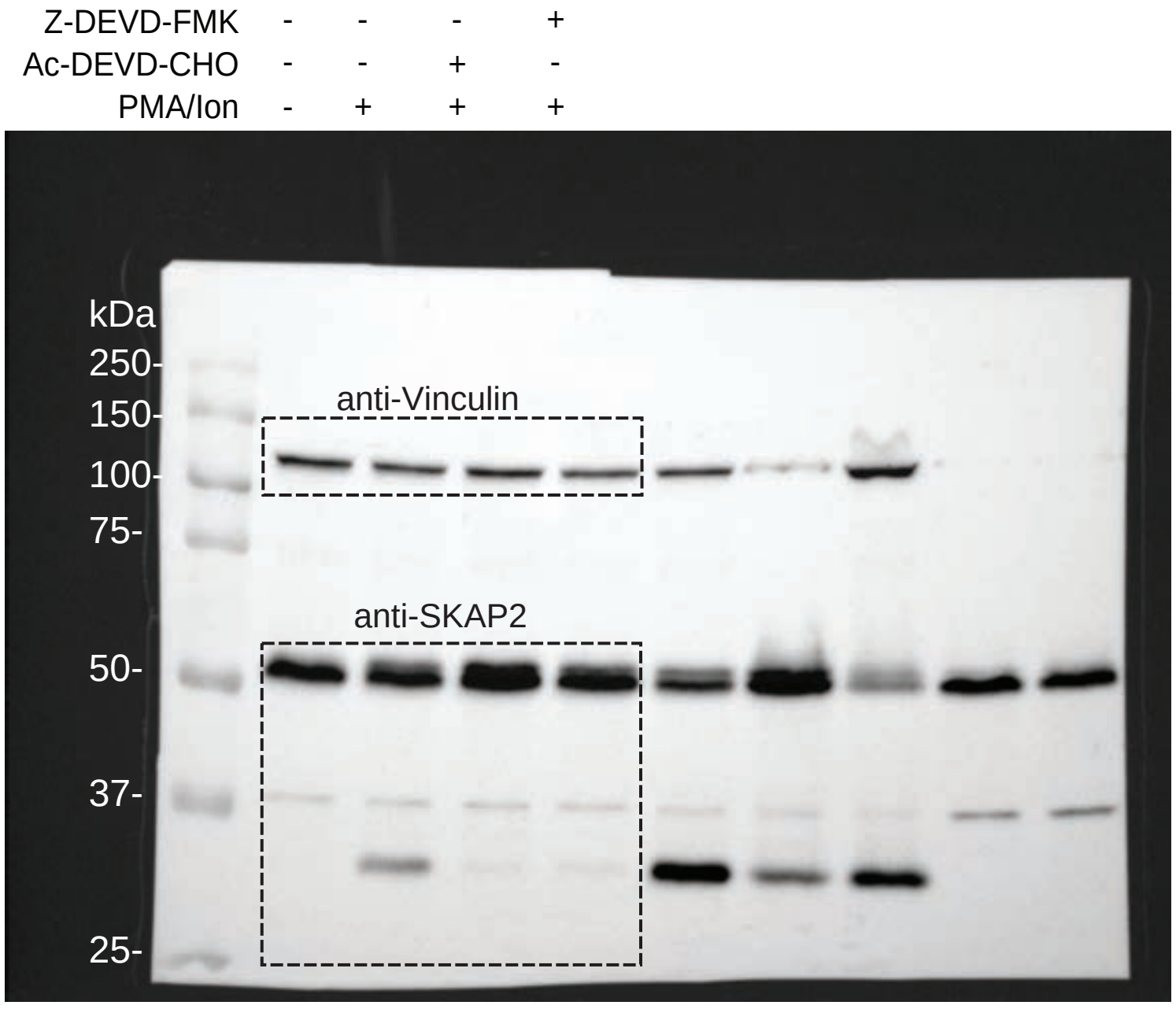

Z-DEVD-FMK - - - +
Ac-DEVD-CHO - - + -
PMA/Ion - + + +
kDa
250-
150-
100-
75-
50-
37-
25-
anti-Vinculin
anti-SKAP2

G

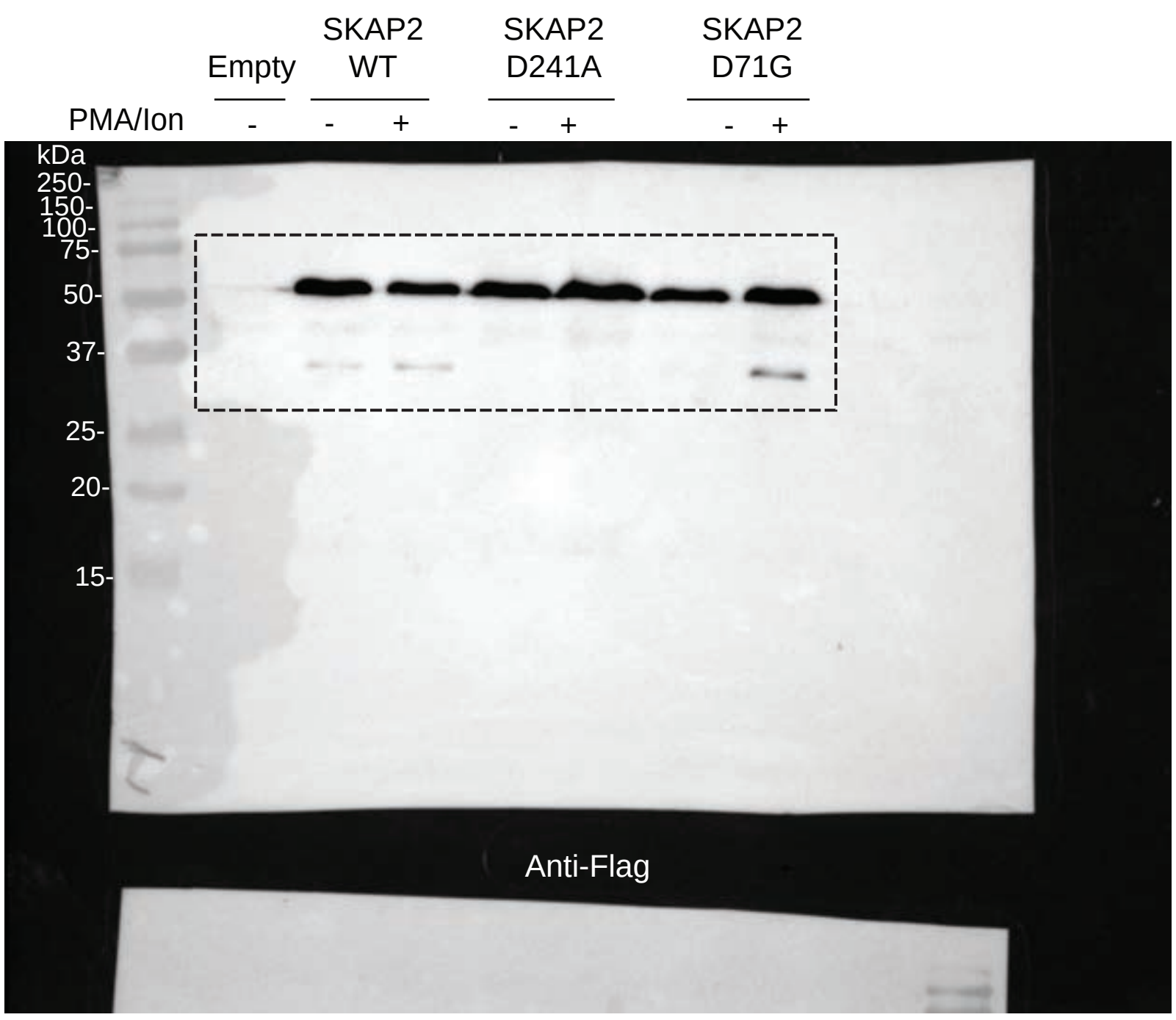

Empty
SKAP2 WT
SKAP2 D241A
SKAP2 D71G
PMA/Ion - - + - + - +
kDa
250-
150-
100-
75-
50-
37-
25-
20-
15-
Anti-Flag

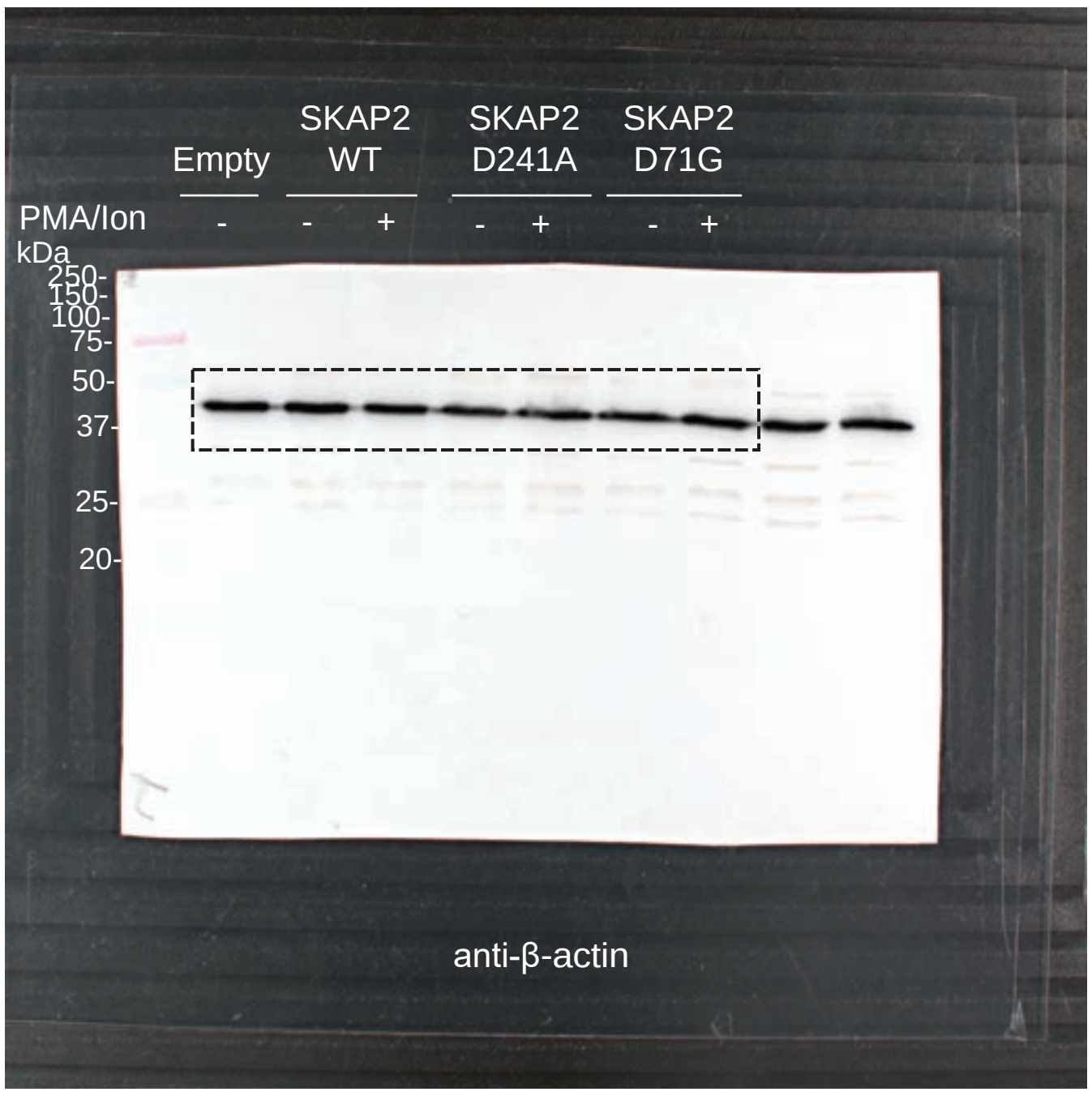

Empty
SKAP2 WT
SKAP2 D241A
SKAP2 D71G
PMA/Ion - - + - + - +
kDa
250-
150-
100-
75-
50-
37-
25-
20-
anti-β-actin

H

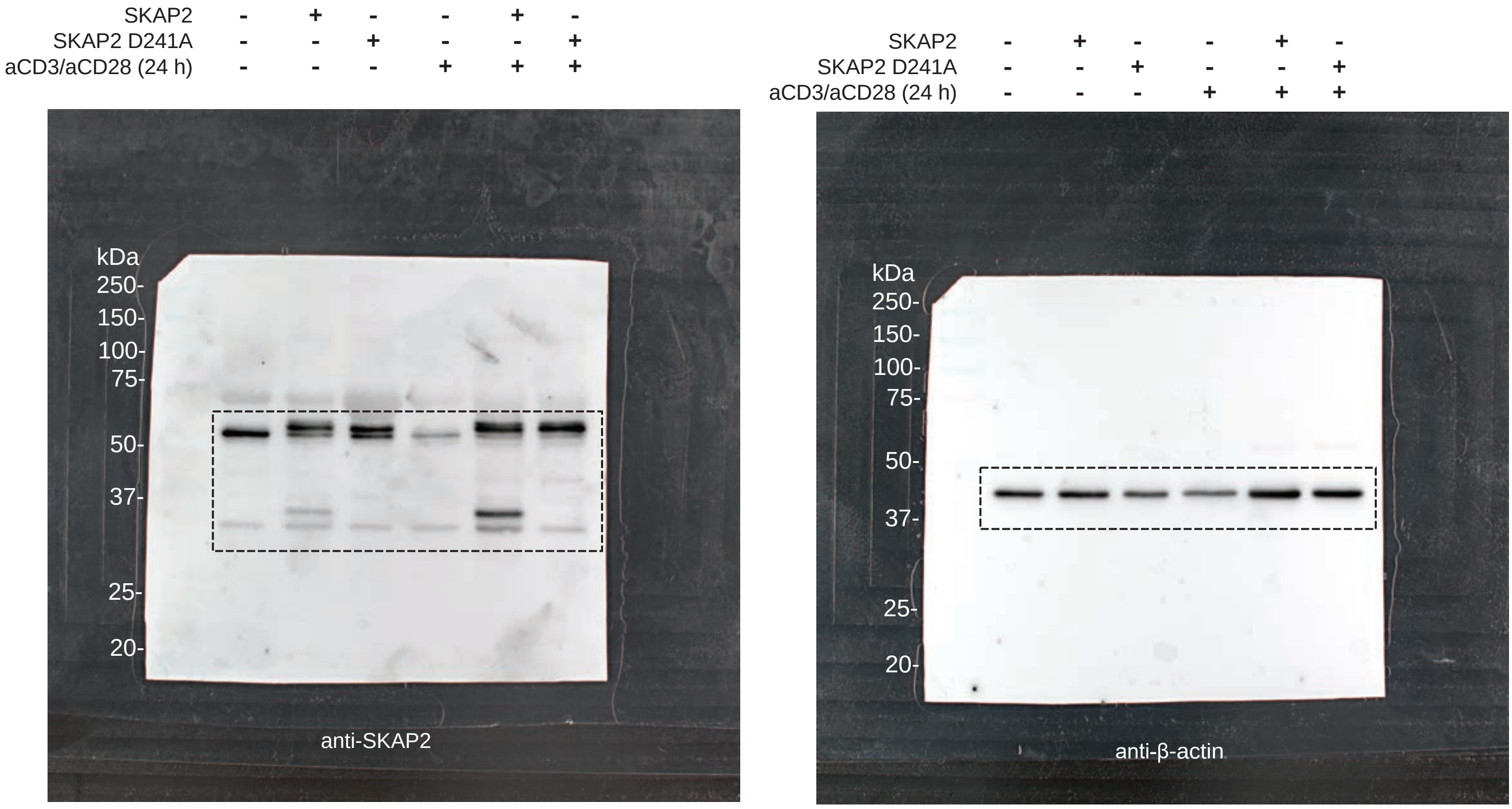

SKAP2 - + - - + -
SKAP2 D241A - - + - - +
aCD3/aCD28 (24 h) - - - + + +
kDa
250-
150-
100-
75-
50-
37-
25-
20-
anti-SKAP2
SKAP2 - + - - + -
SKAP2 D241A - - + - - +
aCD3/aCD28 (24 h) - - - + + +
kDa
250-
150-
100-
75-
50-
37-
25-
20-
anti-β-actin

J

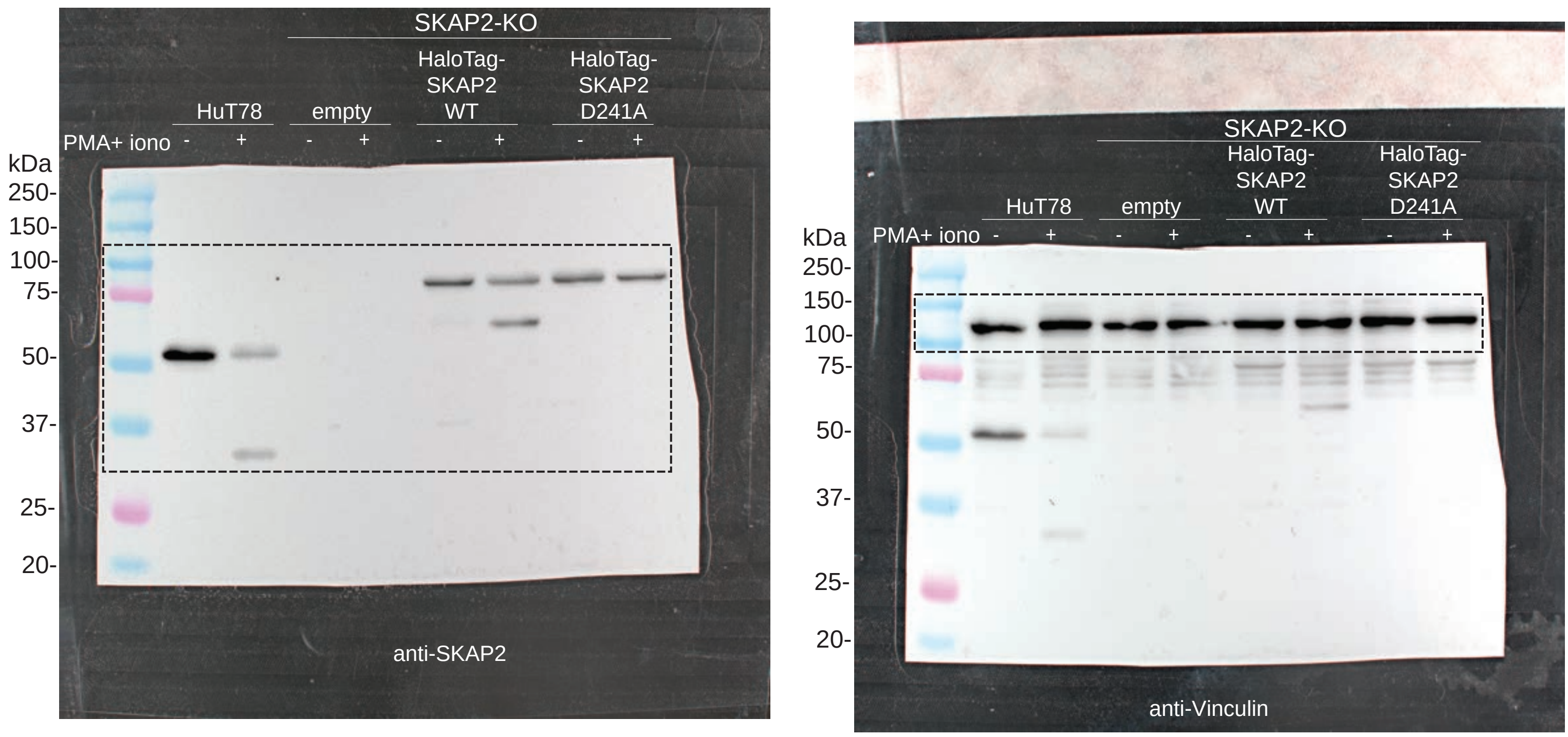

SKAP2-KO
HuT78
empty
HaloTag-SKAP2 WT
HaloTag-SKAP2 D241A
PMA+ iono - + - + - + - +
kDa
250-
150-
100-
75-
50-
37-
25-
20-
anti-SKAP2
SKAP2-KO
HuT78
empty
HaloTag-SKAP2 WT
HaloTag-SKAP2 D241A
PMA+ iono - + - + - + - +
kDa
250-
150-
100-
75-
50-
37-
25-
20-
anti-Vinculin
