# Supplementary material for: A genetically regulated SKAP2 switch modulates human T lymphocyte adhesion and migration
Source: J Exp Med. 2026 Oct 20;223(12):e20260507. doi: 10.1084/jem.20260507 (PMC13682646; doi:10.1084/jem.20260507)
Supplement: Supplementary file 4 [file jem_20260507_sourcedataf7.pdf]

A

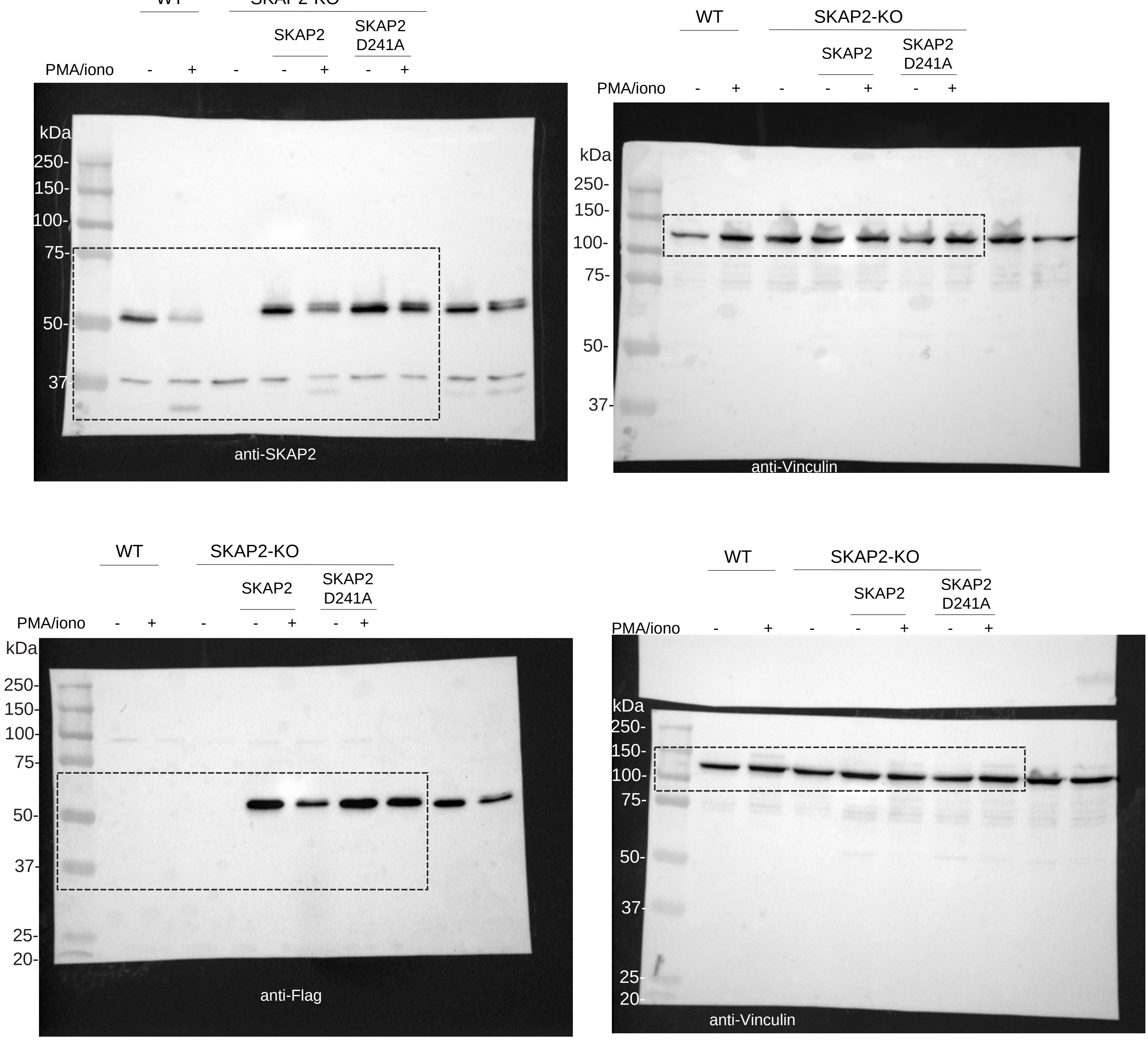
WT
SKAP2-KO
SKAP2
SKAP2 D241A
PMA/iono - + - - + - +
kDa
250-
150-
100-
75-
50-
37
anti-SKAP2
WT
SKAP2-KO
SKAP2
SKAP2 D241A
PMA/iono - + - - + - +
kDa
250-
150-
100-
75-
50-
37-
anti-Vinculin
WT
SKAP2-KO
SKAP2
SKAP2 D241A
PMA/iono - + - - + - +
kDa
250-
150-
100-
75-
50-
37-
25-
20-
anti-Flag
WT
SKAP2-KO
SKAP2
SKAP2 D241A
PMA/iono - + - - + - +
kDa
250-
150-
100-
75-
50-
37-
25-
20-
anti-Vinculin

B

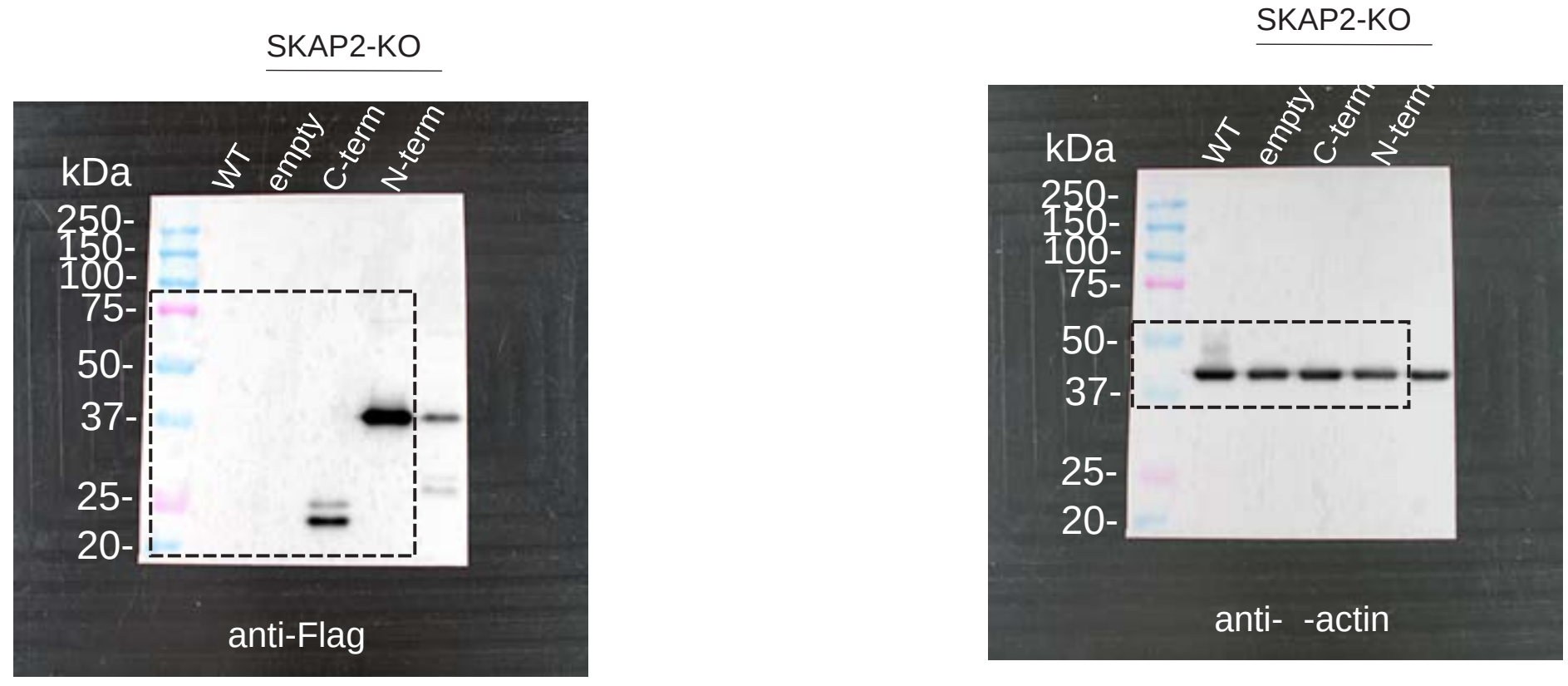
SKAP2-KO
kDa
WT
empty
C-term
N-term
250-
150-
100-
75-
50-
37-
25-
20-
anti-Flag
SKAP2-KO
kDa
WT
empty
C-term
N-term
250-
150-
100-
75-
50-
37-
25-
20-
anti- -actin

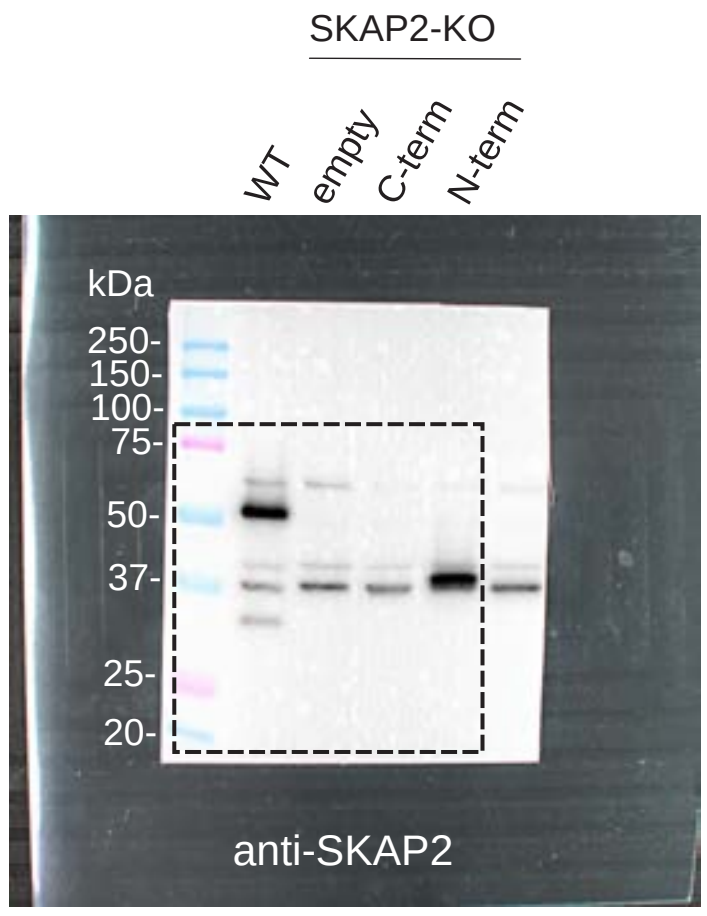
SKAP2-KO
WT
empty
C-term
N-term
kDa
250-
150-
100-
75-
50-
37-
25-
20-
anti-SKAP2

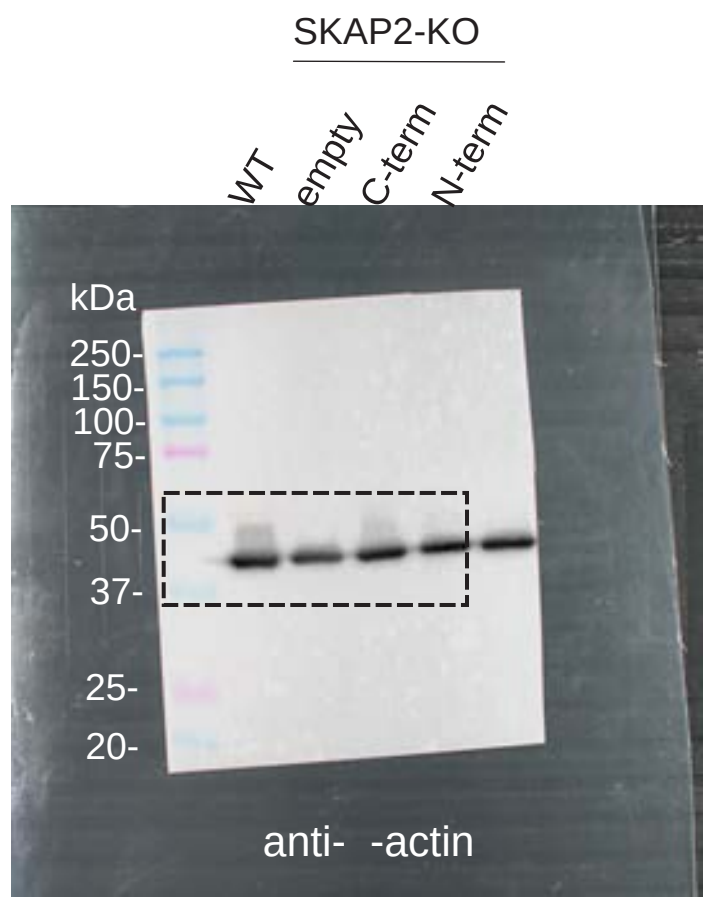
SKAP2-KO
WT
empty
C-term
N-term
kDa
250-
150-
100-
75-
50-
37-
25-
20-
anti- -actin

E

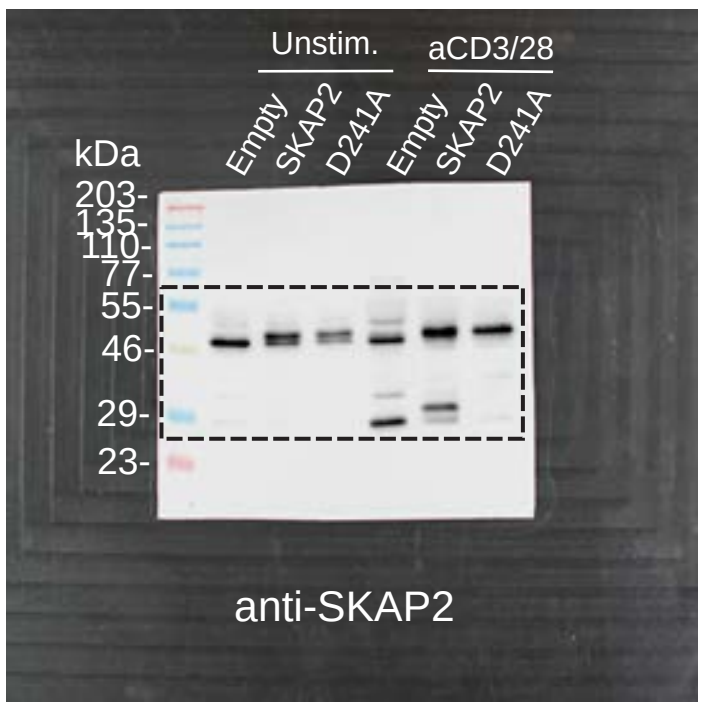
Unstim.
aCD3/28
Empty
SKAP2
D241A
Empty
SKAP2
D241A
kDa
203-
135-
110-
77-
55-
46-
29-
23-
anti-SKAP2

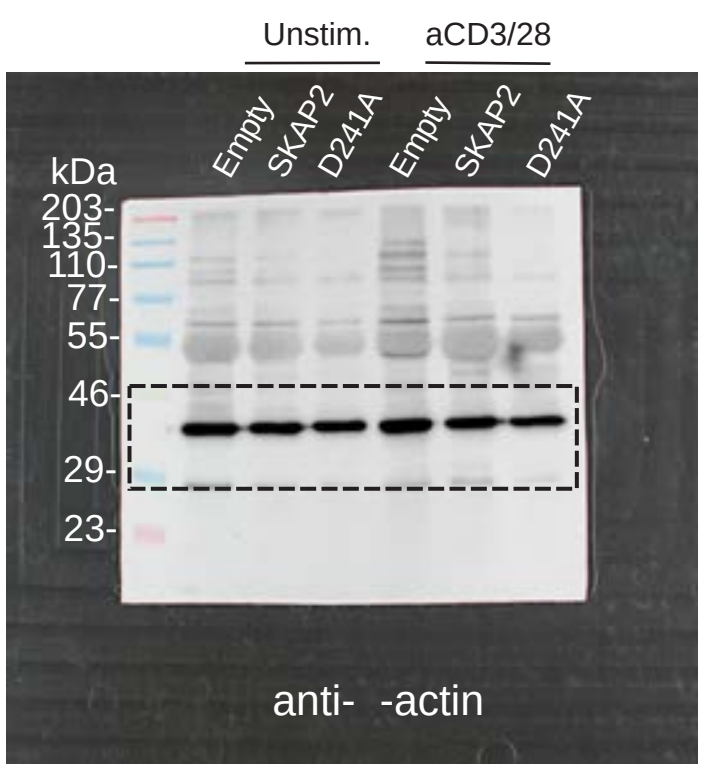
Unstim.
aCD3/28
Empty
SKAP2
D241A
Empty
SKAP2
D241A
kDa
203-
135-
110-
77-
55-
46-
29-
23-
anti- -actin
