# Supplementary material for: A genetically regulated SKAP2 switch modulates human T lymphocyte adhesion and migration
Source: J Exp Med. 2026 Oct 20;223(12):e20260507. doi: 10.1084/jem.20260507 (PMC13682646; doi:10.1084/jem.20260507)
Supplement: Supplementary file 5 [file jem_20260507_sourcedatafs3.pdf]

F

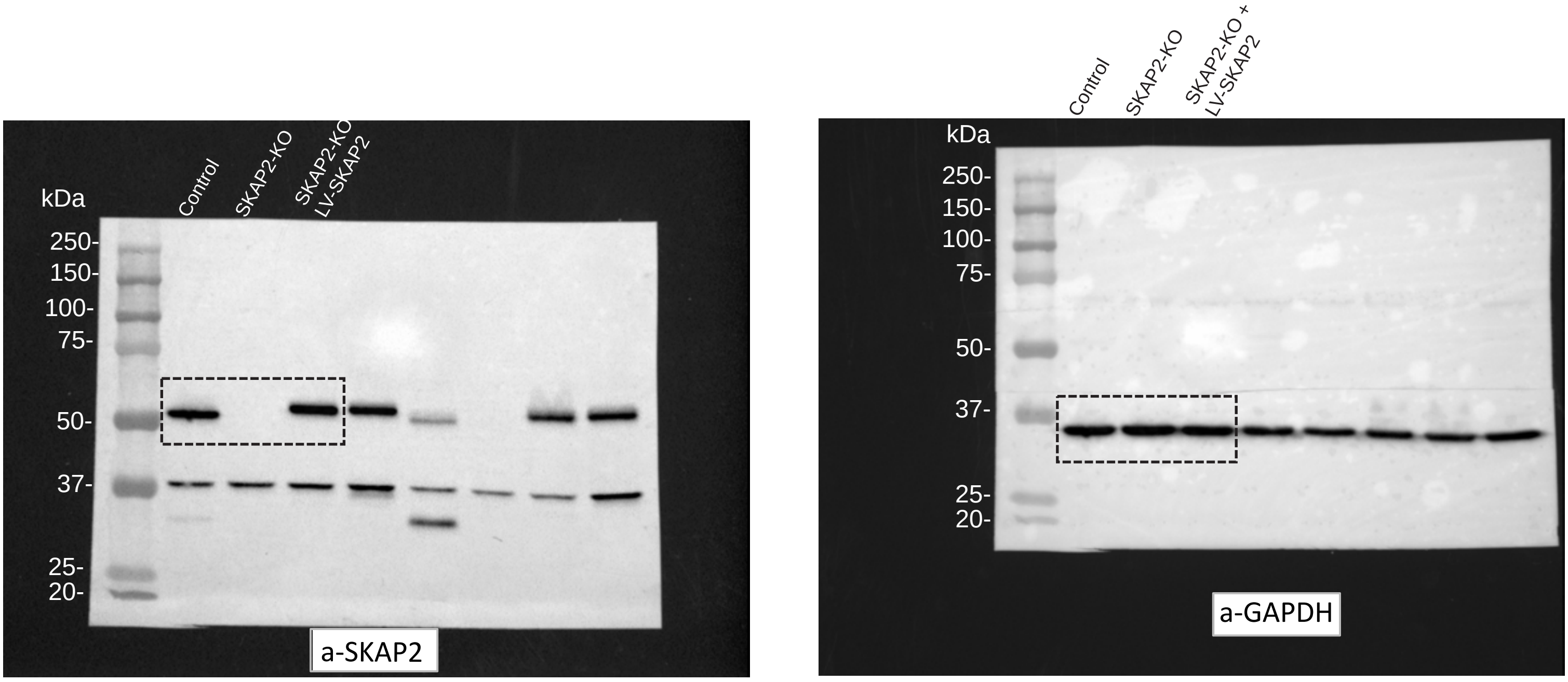

HuT78

K

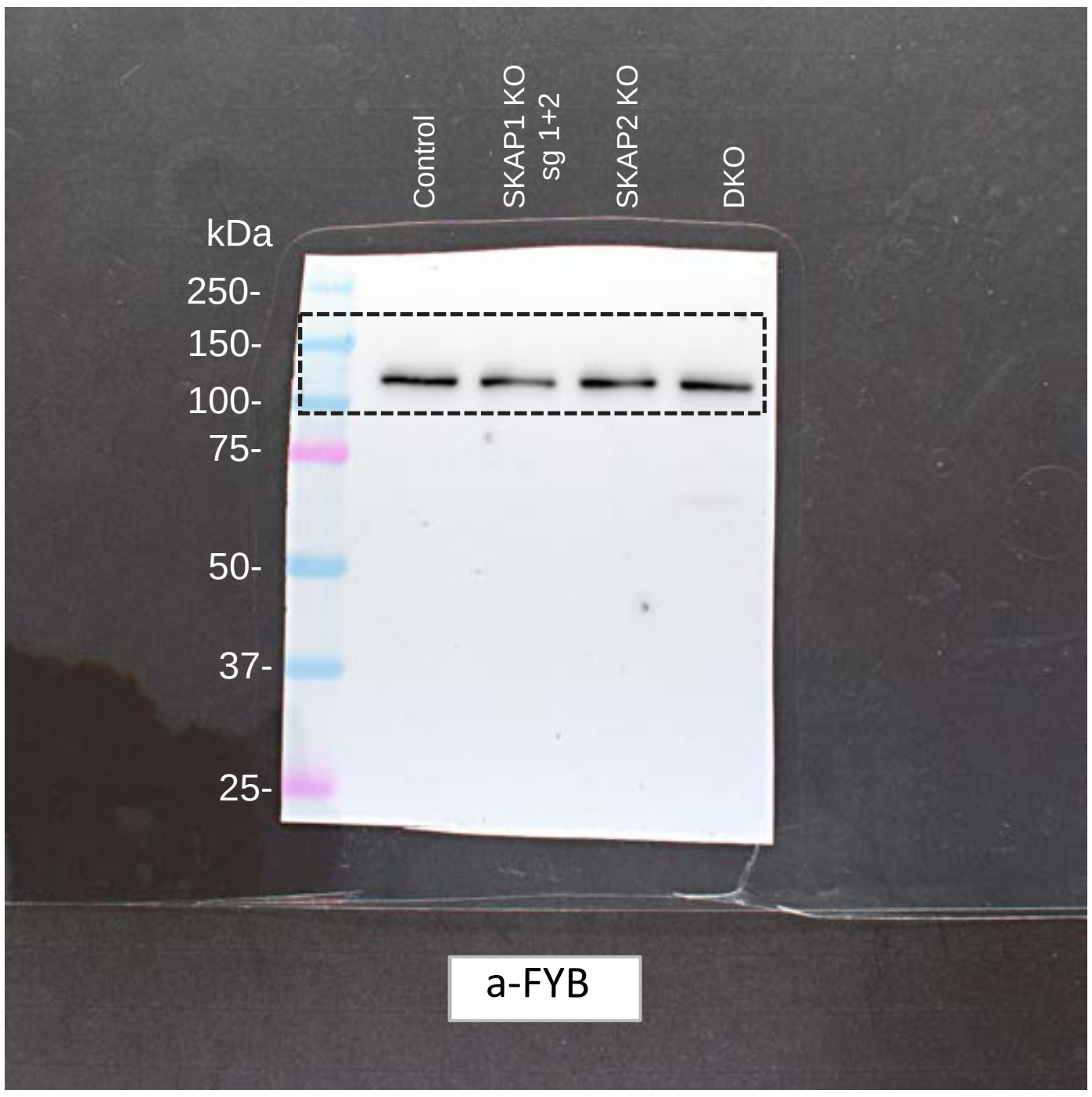

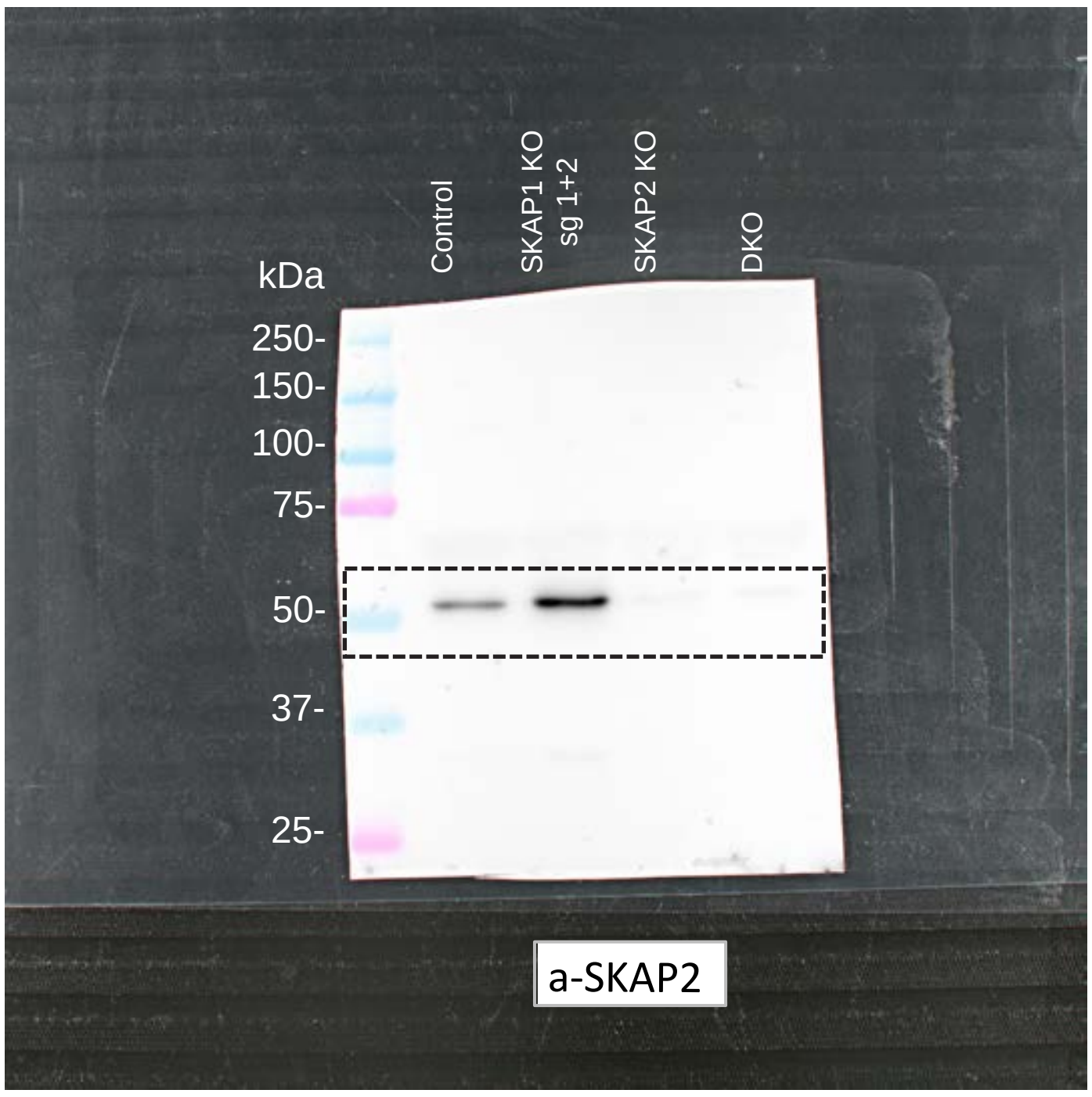

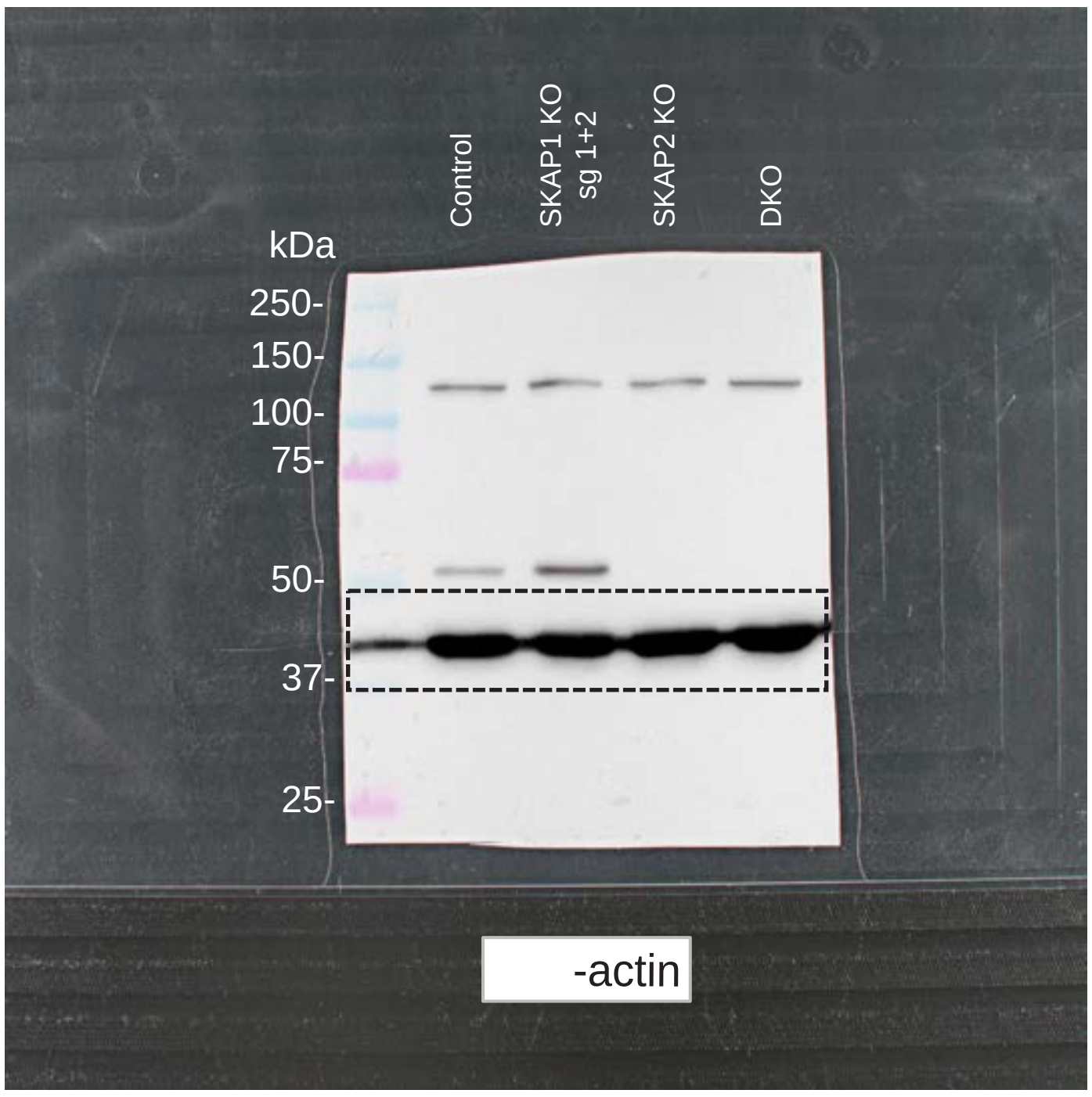

Primary CD4^{+} T memory cells
