# Supplementary material for: A genetically regulated SKAP2 switch modulates human T lymphocyte adhesion and migration
Source: J Exp Med. 2026 Oct 20;223(12):e20260507. doi: 10.1084/jem.20260507 (PMC13682646; doi:10.1084/jem.20260507)
Supplement: Supplementary file 7 [file jem_20260507_sourcedatafs5.pdf]

B

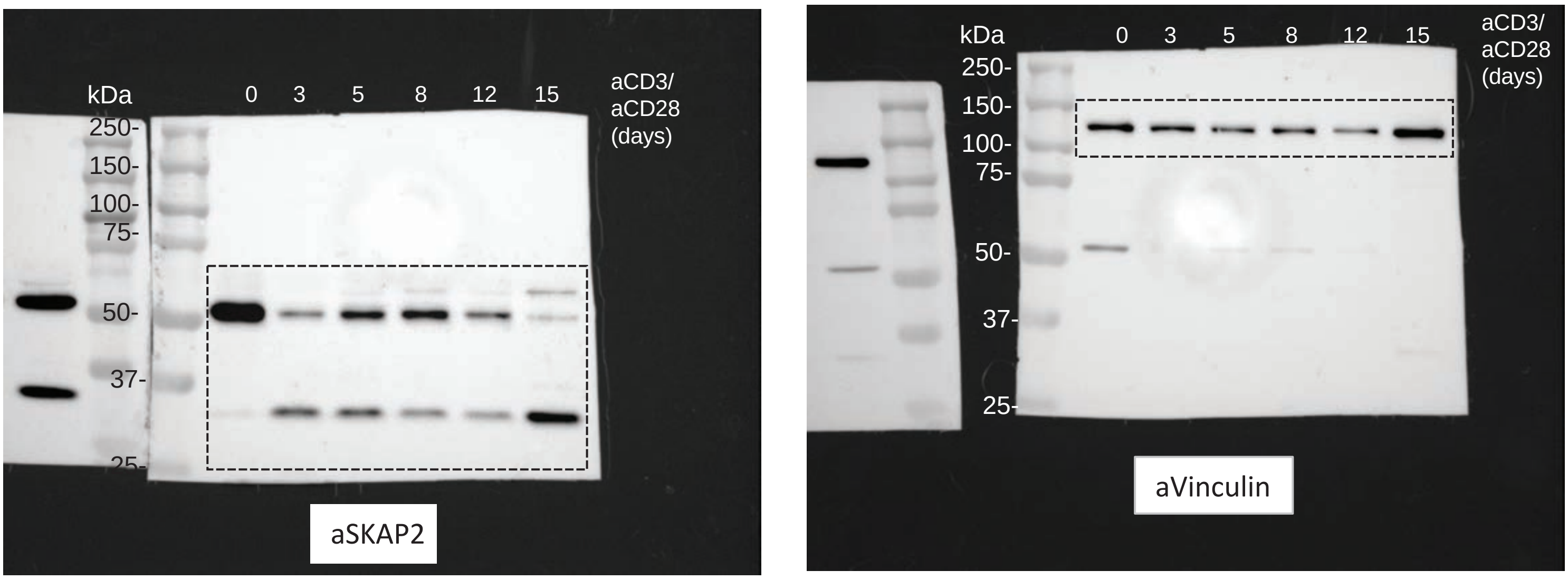
kDa
250-
150-
100-
75-
50-
37-
25-
0
3
5
8
12
15
aCD3/
aCD28
(days)
aSKAP2
kDa
250-
150-
100-
75-
50-
37-
25-
0
3
5
8
12
15
aCD3/
aCD28
(days)
aVinculin

C

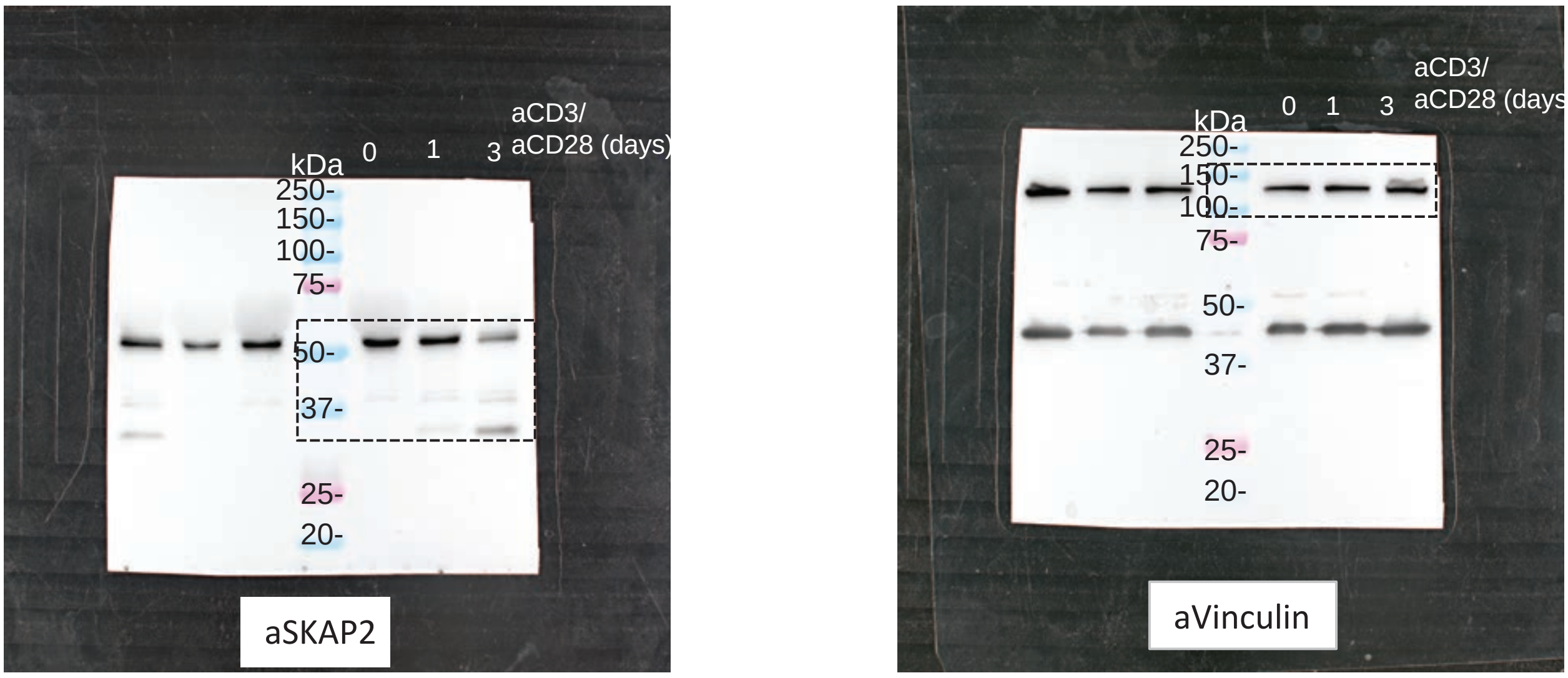
kDa
250-
150-
100-
75-
50-
37-
25-
20-
0
1
3
aCD3/
aCD28 (days)
aSKAP2
kDa
250-
150-
100-
75-
50-
37-
25-
20-
0
1
3
aCD3/
aCD28 (days
aVinculin

D

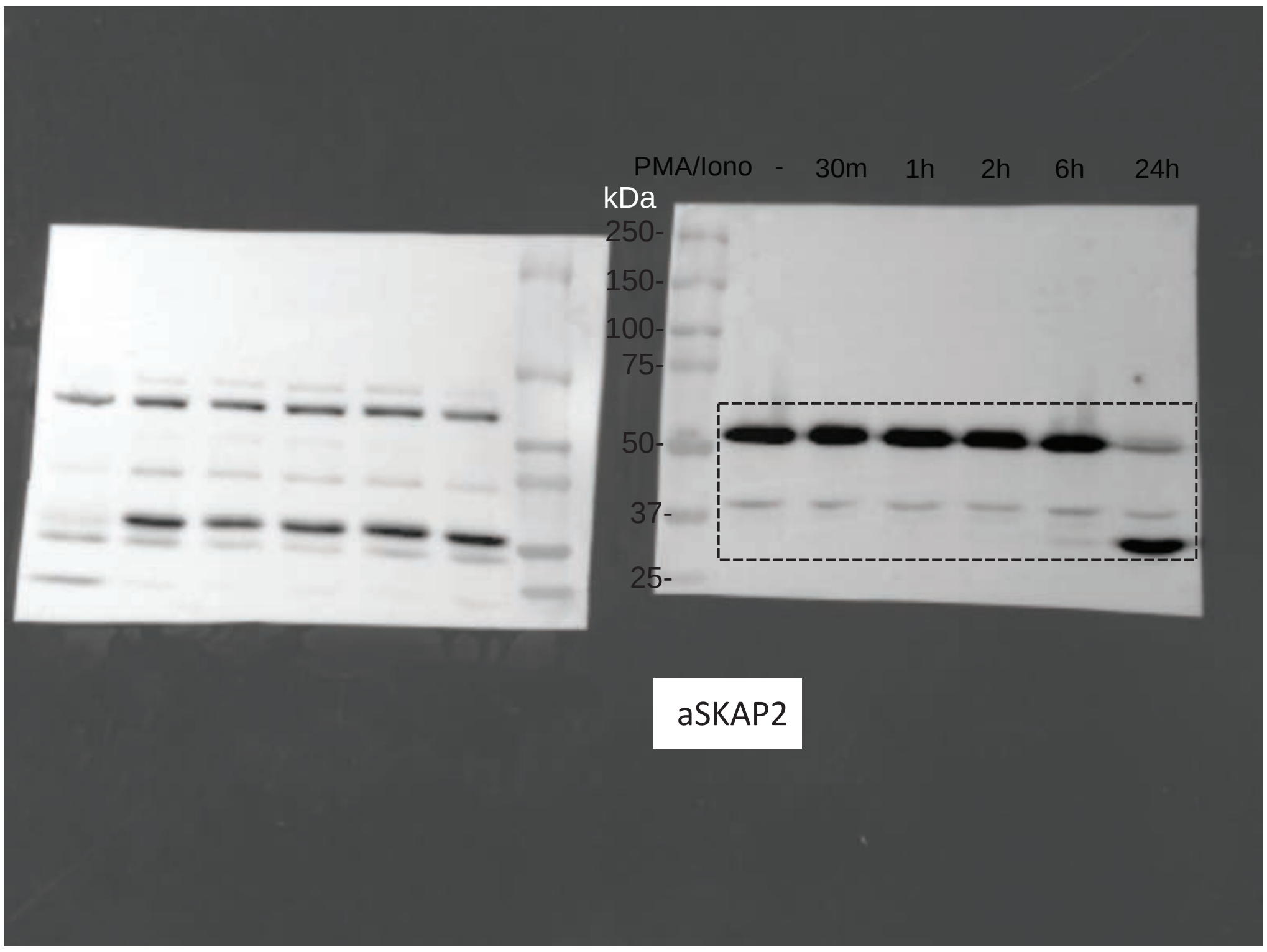
PMA/Iono
-
30m
1h
2h
6h
24h
kDa
250-
150-
100-
75-
50-
37-
25-
aSKAP2

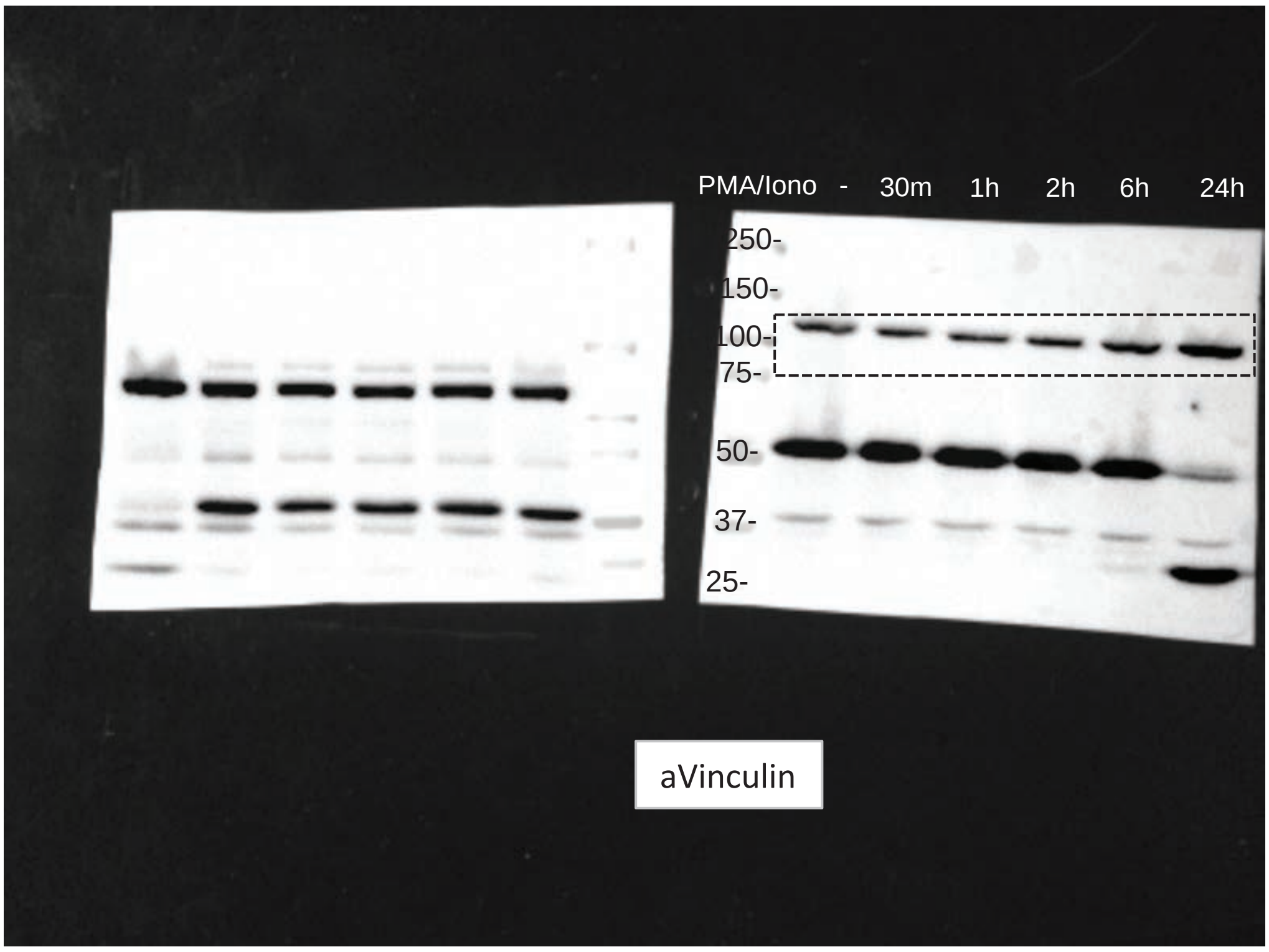

PMA/Iono - 30m 1h 2h 6h 24h
250-
150-
100-
75-
50-
37-
25-
aVinculin

E

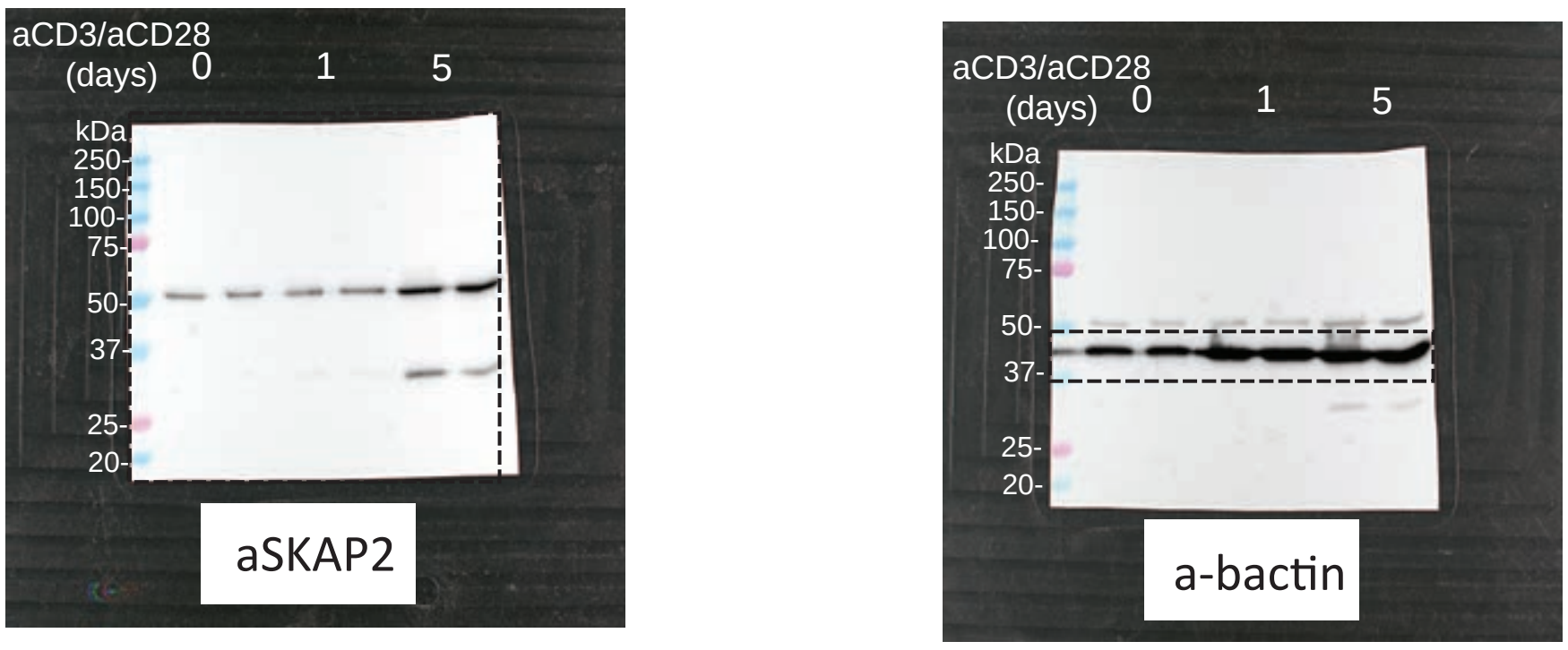

aCD3/aCD28 (days) 0 1 5
kDa
250-
150-
100-
75-
50-
37-
25-
20-
aSKAP2
aCD3/aCD28 (days) 0 1 5
kDa
250-
150-
100-
75-
50-
37-
25-
20-
a-bactin

G

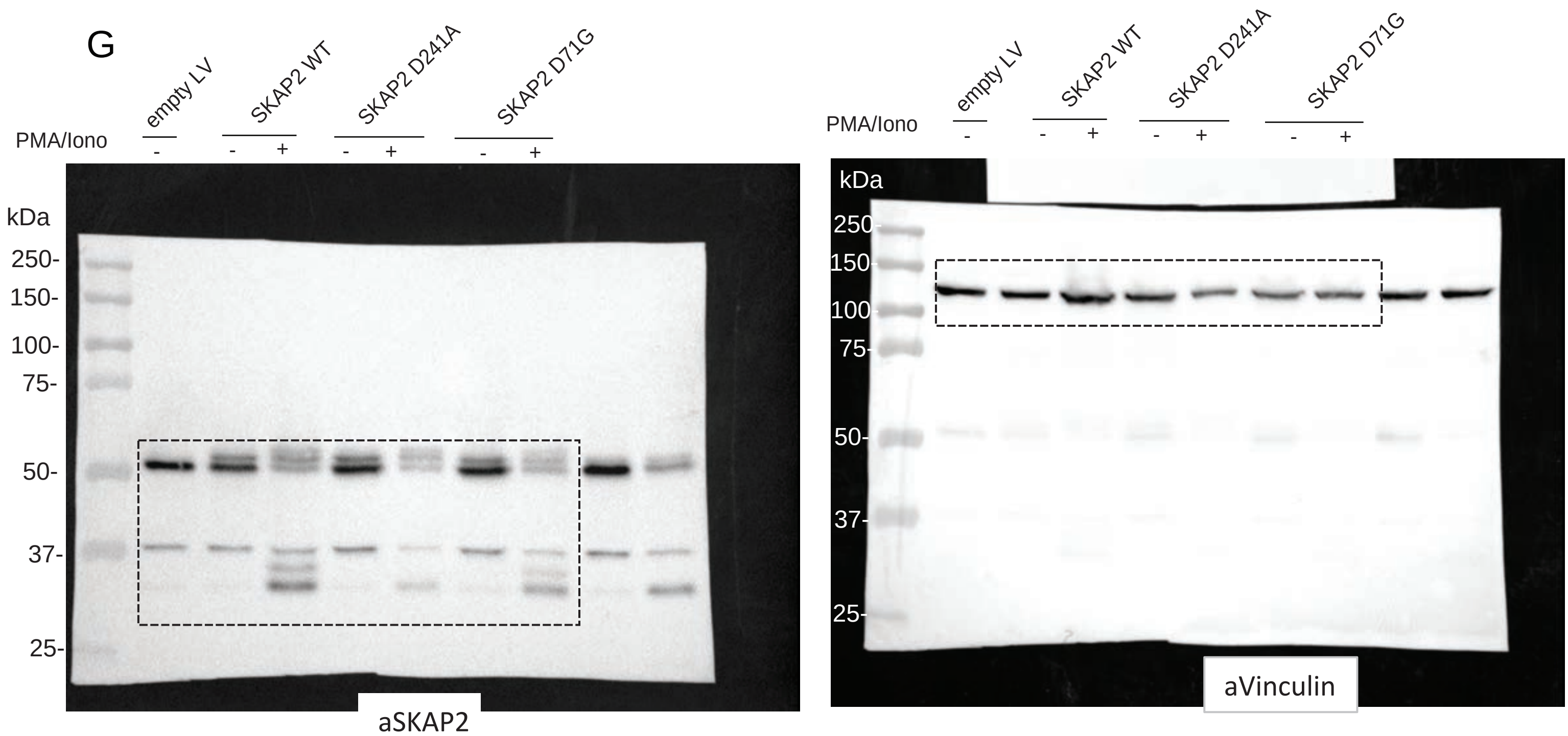

empty LV
SKAP2 WT
SKAP2 D241A
SKAP2 D71G
PMA/Iono - - + - + - +
kDa
250-
150-
100-
75-
50-
37-
25-
aSKAP2
empty LV
SKAP2 WT
SKAP2 D241A
SKAP2 D71G
PMA/Iono - - + - + - +
kDa
250
150
100
75
50
37
25
aVinculin
