# Supplementary material for: A genetically regulated SKAP2 switch modulates human T lymphocyte adhesion and migration
Source: J Exp Med. 2026 Oct 20;223(12):e20260507. doi: 10.1084/jem.20260507 (PMC13682646; doi:10.1084/jem.20260507)
Supplement: Supplementary file 8 [file jem_20260507_tables1.docx]

***Supplementary Table 1. Materials used in this study***

| **REAGENT or RESOURCE** | **SOURCE** | **IDENTIFIER** |
| --- | --- | --- |
| Antibodies | | |
| Mouse monoclonal anti-human CD4 PE-Texas Red | Invitrogen, ThermoFisher Scientific | Catalog # MHCD0417; clone S3.5 |
| Monoclonal anti-human CD25 PC5 | Beckman Coulter | Catalog # IM2646U; clone B1.49.9 |
| Mouse monoclonal anti-human CD45RA Qdot 655 Conjugate | Invitrogen, ThermoFisher Scientific | Catalog # Q10069; clone MEM-56 |
| Mouse monoclonal anti-human CD197 (CCR7) Brilliant Violet 421 | BioLegend | Catalog # 353208; clone G043H7 |
| Mouse monoclonal anti-human CD8 FITC | BioLegend | Catalog #344703; clone SK1 |
| Mouse monoclonal anti-human CD3 | Recombinant, prepared in house | Clone TR66 |
| Mouse monoclonal anti-human CD28 | BD Biosciences | Catalog # 555725; clone CD28.2 |
| Mouse monoclonal anti-human IFN-γ APC/Cyanine7 | BioLegend | Catalog # 502530; clone 4S.B3 |
| Brilliant Violet 421 anti-human CD25 Antibody | BioLegend | Catalog # 356113; clone  M-A251 |
| Purified anti-mouse CD3e Monoclonal Antibody (145-2C11) | ThermoFisher Scientific | Catalog # 14-0031-82 |
| Purified anti-mouse CD28 Antibody | BioLegend | Catalog # 102102; clone 37.51 |
| PE anti-mouse CD28 Antibody | BioLegend | Catalog # 102105; clone 37.51 |
| Rat anti-mouse CD4 Antibody APC/Fire 750 | BioLegend | Catalog # 100459; clone GK1.5 |
| Anti-SKAP2 antibody produced in rabbit | Sigma-Aldrich | Catalog # HPA024045 |
| Mouse Anti-Human FYB | BD Biosciences | Catalog # 610944, clone 5 |
| Anti-HaloTag pAb | Promega | Catalog # G9281 |
| Monoclonal ANTI-FLAG M2 antibody produced in mouse | Sigma-Aldrich | Catalog # F1804 |
| Human anti Human Actin Beta:HRP | Bio-Rad Laboratories | Catalog # HCA147P, clone AbD12141 |
| Anti-GAPDH antibody | Abcam | Catalog # ab8245, clone 6C5 |
| Vinculin | Santa Cruz Biotechnology | Catalog # sc-736144, clone 7F9 |
| anti-rabbit IgG (H+L) Alexa Fluor 647 | Sigma-Aldrich | Catalog # SAB4600393 |
| Goat Anti-Mouse IgG1-FITC, | Southernbiotech | Catalog # 1070-02 |
| Goat anti-Mouse IgG (H+L) Alexa Fluor 647 | Sigma-Aldrich | Catalog # A-21236 |
| Rabbit IgG | Cell Signaling Technology | Catalog # 2729 |
| anti-rabbit IgG-HRP | Cell Signaling Technology | Catalog # sc-7074 |
| anti-mouse IgG-HRP | Cell Signaling Technology | Catalog # 7076 |
| BrdU Monoclonal Antibody (BU20A), eFluor 450 | eBioscience, Invitrogen | Catalog # 48-5071-42 |
| SKAP55 Antibody (35) | Santa Cruz Biotechnology | Catalog# sc-136068 |
| SKAP2 Polyclonal antibody | Proteintech Europe | Catalog # 12926-1-AP |
| SKAP55 Polyclonal antibody | Proteintech Europe | Catalog # 19247-1-AP |
| α-Adaptin 1/2 Antibody (C-8) | Santa Cruz Biotechnology | Catalog # sc-17771 |
| CD14-FITC | Beckman Coulter | Catalog # B36297 |
|  |  |  |
| Chemicals, peptides, and recombinant proteins | | |
| Human IL-2 | Recombinant, prepared in house |  |
| ICAM1/CD54 human | Sigma-Aldrich | Catalog # SRP6491 |
| Dynabeads T Cell Activation kit | ThermoFisher Scientific | Catalog # 11132D |
| Phorbol 12-myristate 13-acetate (PMA) | Sigma-Aldrich | Catalog # P1585 |
| Ionomycin calcium salt | Sigma-Aldrich | Catalog # I0634 |
| Brefeldin A | Sigma-Aldrich | Catalog # B6542 |
| Ficoll-Paque PLUS Medium | Sigma-Aldrich | Catalog # GE17-1440-03 |
| z-VAD-FMK (N-Benzyloxycarbonyl-Val-Ala-Asp(O-Me) fluoromethyl ketone) | Selleckchem | Catalog # S7023 |
| Z-DEVD-FMK (Benzyloxycarbonyl-Asp(OMe)-Glu(OMe)-Val-Asp(OMe)-fluoromethylketone) | Selleckchem | Catalog # S7312 |
| Ac-DEVD-CHO | Selleckchem | Catalog # S7901 |
| Dulbecco's Modified Eagle Medium (DMEM) | Gibco | Catalog # 61965059 |
| Roswell Park Memorial Institute (RPMI) 1640 Medium | Gibco | Catalog # 31870025 |
| Fetal bovine serum | Gibco | Catalog # 10270106 |
| GlutaMAX supplement | Gibco | Catalog # 35050038 |
| MEM Non-Essential Amino Acids | Gibco | Catalog # 11140068 |
| Penicillin-Streptomycin | Gibco | Catalog # 15070063 |
| Sodium pyruvate | Gibco | Catalog # 11360088 |
| β-mercaptoethanol | Gibco | Catalog # 31350010 |
| Polyethylenimine (PEI) | Polysciences Inc | Catalog # 23966 |
| True Cut Cas9 Protein v2 | Thermo Fisher Scientific | Catalog # A36499 |
| Alt-R CRISPR-Cas9 tracrRNA | Integrated DNA Technologies | Catalog # 1072532 |
| Alt-R CRISPR-Cas9 tracrRNA, ATTO 550 | Integrated DNA Technologies | Catalog # 1075927 |
| Nuclease-free duplex buffer | Integrated DNA Technologies | Catalog # 11-01-03-01 |
| Alt-R CRISPR-Cas9 Electroporation Enhancer | Integrated DNA Technologies | Catalog # 1075915 |
| Poly-glutamic acid sodium salt | Sigma | Catalog # P4761-25MG |
| PEG-8000 | Promega | Catalog # V3011 |
| Laemmli buffer | Biorad Laboratories | Catalog # 1610747 |
| Protease inhibitor cocktail | Sigma-Aldrich | Catalog # P8340 |
| PhosSTOP | Sigma-Aldrich | Catalog # 4906845001 |
| Triton X-100 | Sigma-Aldrich | Catalog # X100 |
| Glycine | Sigma-Aldrich | Catalog # G7126 |
| Clarity Western ECL Substrate | Bio-Rad | Catalog # 1705060 |
| Dynabeads Protein G | Thermo Fisher Scientific | Catalog # 10003D |
| Halo-Trap Agarose beads | Cromotek | Catalog # ota |
| Formaldehyde | Sigma | Catalog # F8775 |
| KOD Hot Start DNA Polymerase | Sigma-Aldrich | Catalog # 71086 |
| Q5 High-Fidelity DNA Polymerase | New England Biolabs | Catalog # M0491S |
| SuRE/Cut Buffer A | Roche | Catalog # 11417959001 |
| RNasin Plus RNase Inhibitor | Promega | Catalog # N2615 |
| TRI Reagent RT | Molecular Research Center, Inc. | Catalog # RT111 |
| TaqMan SNP Genotyping Assay, human - SNP ID: rs17323934 | Thermo Fisher Scientific | Catalog # 4351379, Assay ID C__34585256_10 |
| CD4 (L3T4) MicroBeads, mouse | Miltenyi Biotec | Catalog # 130-117-043 |
| CD8 MicroBeads, human | Miltenyi Biotec | Catalog # 130-045-201 |
| CD4 MicroBeads, human | Miltenyi Biotec | Catalog # 130-045-101 |
| CD14 MicroBeads, human | Miltenyi Biotec | Catalog # 130-050-201 |
| Recombinant Human SDF-1/CXCL12 Protein (isoform a) | Sino Biological | Catalog # 13511-HNCE |
| TRIzol Reagent | Invitrogen | Catalog # 15596026 |
| RNase-Free DNase Set | Qiagen | Catalog # 79254 |
| Collagen Type I, bovine, 5mg/ml, 1x5ml | IBIDI | Catalog # 50201 |
|  |  |  |
| Commercial kits | | |
| Quick RNA Miniprep kit | Zymo Research | Catalog # R1054 |
| Direct-zol RNA Microprep kit | Zymo Research | Catalog # R2062 |
| qScript cDNA SuperMix | Quantabio | Catalog # 733-1177 |
| PerfeCTa SYBR Green FastMix | Quantabio | Catalog # 95055-500 |
| DNeasy Blood & Tissue Kit | Qiagen | Catalog #69504 |
| Pierce BCA Protein Assay Kit | Thermo Fisher Scientific | Catalog # 23227 |
| DC Protein Assay | Bio-Rad | Catalog # 5000111 |
| iST Sample Preparation Kit | Preomics | Catalog # P.O.00027 |
| Neon Transfection System 10 µL Kit | Thermo Fisher Scientific | Catalog # MPK1025 |
| Neon Transfection System 100 µL Kit | Thermo Fisher Scientific | Catalog # MPK10025 |
| P3 Primary Cell 4D-Nucleofector X Kit S | Lonza Bioscience | Catalog # LZ-V4XP-3032 |
| Q5 Site-Directed Mutagenesis Kit | New England Biolabs | Catalog # E0554S |
| Qubit RNA High Sensitivity assay kit | Thermo Fisher Scientific | Catalog # Q32852 |
| TruSeq Stranded Total RNA Sample preparation kit | Illumina | Catalog # RS-122-9007 |
| Quantifluor dsDNA | Promega | Catalog # E2670 |
| IntraSure Kit | BD Biosciences | Catalog # 641778 |
| LIVE/DEAD Fixable Blue Dead Cell Stain Kit, for UV excitation | Invitrogen, ThermoFisher Scientific | Catalog # L23105 |
| BrdU Flow Kit | BD Biosciences | Catalog # 552598 |
| High Sensitivity RNA ScreenTape Assay | Agilent | Catalog # 5067-5579 |
| Illumina TruSeq Stranded Total RNA Library Prep Gold Kit | Illumina | Catalog # 20020598 |
| Agilent TapeStation D5000 ScreenTape Assay | Agilent | Catalog # 5067-5592 |
|  |  |  |
|  |  |  |
| Deposited data | | |
| Gene Expression Omnibus (GEO) | accession number **GSE288974** |  |
| Experimental models: | | |
| HEK293T | ATCC | CRL-3216 |
| HuT78 | ATCC | TIB-161 |
| Jurkat, Clone E6-1 | ATCC | TIB-152 |
| DND41 | DSMZ | ACC 525 |
| C57BL/6 mice | Charles River Laboratories Italia |  |
| DH10B Competent Cells | Thermo Fisher Scientific | Catalog # EC0113 |
| Oligonucleotides | | |
| Primer for pScalps Flag-SKAP2 D71G mutagenesis, Forward 5’- GAATTATATGATGATGTTGGTCATCCTCTACCAATAAGC -3’ | This paper | N/A |
| Primer for pScalps Flag-SKAP2 D71G mutagenesis, Reverse 5’- GCTTATTGGTAGAGGATGACCAACATCATCATATAATTC -3’ | This paper | N/A |
| Primer for pScalps Flag-SKAP2 D241A mutagenesis, Forward 5’- GAATTATATGATGATGTTGCTCATCCTCTACCAATAAGC -3’ | This paper | N/A |
| Primer for pScalps Flag-SKAP2 D241A mutagenesis, Reverse 5’- GCTTATTGGTAGAGGATGAGCAACATCATCATATAATTC -3’ | This paper | N/A |
| Primer for S*KAP2* cDNA Forward 5’- AACCCCAGCAGCACCTCCTCTC -3’ | This paper | N/A |
| Primer for S*KAP2* cDNA Reverse 5’- ATCTCCATTATGTAGGCTTTAGG -3’ | This paper | N/A |
| Primer for HaloTag-SKAP2 cloning into pScalps-Puro (BamHI- NotI digestion) Forward 5’- GCTGGATCCGACACCATGGCAGAAATCGGTACTGGCTTTCC -3’ | This paper | N/A |
| Primer for HaloTag-SKAP2 cloning into pScalps-Puro Reverse Fusion 5’- TCCTCCCGATCCGCCGGAAATCTCGAGCGTCGAC -3’ | This paper | N/A |
| Primer for HaloTag-SKAP2 cloning into pScalps-Puro Forward Fusion 5’- ATTTCCGGCGGATCGGGAGGACCCAACCCCAGCAGCACCTCCTCTC -3’ | This paper | N/A |
| Primer for HaloTag-SKAP2 cloning into pScalps-Puro Reverse (BamHI- NotI digestion) 5’- CTGTGCTGGGCGGCCGCTCAAATATCATACATCTCCATTATGTAGGCTTTAGGC -3’ | This paper | N/A |
| Primer for HaloTag cloning into pScalps-Puro Forward (BamHI- NotI digestion) 5’- GCTGGATCCGACACCATGGCAGAAATCGGTACTGGCTTTCC -3’ | This paper | N/A |
| Primer for HaloTag cloning into pScalps-Puro Reverse (BamHI- NotI digestion) 5’- TGTGCTGGGCGGCCGCTCAGCCGGAAATCTCGAGCGTCG -3’ | This paper | N/A |
| Primer for pScalps HaloTag-SKAP2 D241A mutagenesis, Forward 5’- CACTAACAAGCAGTCAACCGATCGATGATGAAATTTATGAAGAACTTC -3’ | This paper | N/A |
| Primer for pScalps HaloTag -SKAP2 D241A mutagenesis, Reverse 5’- GATTGCTTATTGGTAGAGGATGAGCAACATCATCATATAATTCTCC -3’ | This paper | N/A |
| Primer for cloning C-term SKAP2 (242aa-359aa) into pScalps 3xFlag Forward 5’- GCTGGATCCGACACCATGCATCCTCTACCAATAAGCAATCCAC -3’ | This paper | N/A |
| Primer for cloning C-term SKAP2 (242aa-359aa) into pScalps 3xFlag Reverse 5’- CGAGCGGCCGCACAATATCATACATCTCCATTATGTAGGCTTTA -3’ | This paper | N/A |
| Primer for cloning N-term SKAP2 (1aa-241aa) into pScalps 3xFlag Forward 5’- GCTGGATCCGACACCATGCCCAACCCCAGCAGCACCTC -3’ | This paper | N/A |
| Primer for cloning N-term SKAP2 (1aa-241aa) into pScalps 3xFlag Reverse 5’- TATATATGCGGCCGCTTAATCAACATCATCATATAATTCTCCTCTCTCA -3’ | This paper | N/A |
| Primer for cloning sgNTC 1 into pXPR_502 Forward 5’- CACCGTAGCGAACGTGTCCGGCGT -3’ | This paper | N/A |
| Primer for cloning sgNTC 1 into pXPR_502 Reverse 5’- AAACACGCCGGACACGTTCGCTAC -3’ | This paper | N/A |
| Primer for cloning sgNTC 2 into pXPR_502 Forward 5’- CACCGACCGGAACGATCTCGCGTA -3’ | This paper | N/A |
| Primer for cloning sgNTC 2 into pXPR_502 Reverse 5’-  AAACTACGCGAGATCGTTCCGGTC -3’ | This paper | N/A |
| Primer for cloning sgNTC 3 into pXPR_502 Forward 5’- CACCGCTTGAGCACATACGCGAAT -3’ | This paper | N/A |
| Primer for cloning sgNTC 3 into pXPR_502 Reverse 5’- AAACATTCGCGTATGTGCTCAAGC -3’ | This paper | N/A |
| Primer for cloning sgNTC 4 into pXPR_502 Forward 5’- CACCGTGGTAGAATAACGTATTAC -3’ | This paper | N/A |
| Primer for cloning sgNTC 4 into pXPR_502 Reverse 5’- AAACGTAATACGTTATTCTACCAC -3’ | This paper | N/A |
| Primer for cloning sgNTC 5 into pXPR_502 Forward 5’-  CACCGTCATACATGGATAAGGCTA -3’ | This paper | N/A |
| Primer for cloning sgNTC 5 into pXPR_502 Reverse 5’- AAACTAGCCTTATCCATGTATGAC -3’ | This paper | N/A |
| Primer for cloning sgNTC 0 into pXPR_502 Forward 5’- CACCGTAGGCGCGCCGCTCTCTAC  -3’ | This paper | N/A |
| Primer for cloning sgNTC 0 into pXPR_502 Reverse 5’- AAACGTAGAGAGCGGCGCGCCTAC -3’ | This paper | N/A |
| Primer for cloning sgA SKAP2 into pXPR_502 Forward 5’-  CACCGAACAGCGGGGCTACGAGTC -3’ | This paper | N/A |
| Primer for cloning sgA SKAP2 into pXPR_502 Reverse 5’-  AAACGACTCGTAGCCCCGCTGTTC -3’ | This paper | N/A |
| Primer for cloning sgB SKAP2 into pXPR_502 Forward 5’-  CACCGCTACGAGTCGGGACACTGCC -3’ | This paper | N/A |
| Primer for cloning sgB SKAP2 into pXPR_502 Reverse 5’-  AAACGGCAGTGTCCCGACTCGTAGC -3’ | This paper | N/A |
| Primer for cloning sgC SKAP2 into pXPR_502 Forward 5’-  CACCGCCGGGCCGGGGCTCACAACA -3’ | This paper | N/A |
| Primer for cloning sgC SKAP2 into pXPR_502 Reverse 5’-  AAACTGTTGTGAGCCCCGGCCCGGC -3’ | This paper | N/A |
| Primer for cloning sgD SKAP2 into pXPR_502 Forward 5’-  CACCGACAGTCCAGCTGCAGCTCGC -3’ | This paper | N/A |
| Primer for cloning sgD SKAP2 into pXPR_502 Reverse 5’-  AAACGCGAGCTGCAGCTGGACTGTC -3’ | This paper | N/A |
| Primer for cloning sgE SKAP2 into pXPR_502 Forward 5’-  CACCGAGGGCCGGGGCGGGGAGAT -3’ | This paper | N/A |
| Primer for cloning sgE SKAP2 into pXPR_502 Reverse 5’-  AAACATCTCCCCGCCCCGGCCCTC -3’ | This paper | N/A |
| Primer for cloning sgF SKAP2 into pXPR_502 Forward 5’-  CACCGGGACACGAAGGGCCTGAG -3’ | This paper | N/A |
| Primer for cloning sgF SKAP2 into pXPR_502 Reverse 5’-  AAACCTCAGGCCCTTCGTGTCCC -3’ | This paper | N/A |
| Primer for cloning sgG SKAP2 into pXPR_502 Forward 5’-  CACCGTGTATTCCTCGGTTTTCC -3’ | This paper | N/A |
| Primer for cloning sgG SKAP2 into pXPR_502 Reverse 5’-  AAACGGAAAACCGAGGAATACAC -3’ | This paper | N/A |
| Primer for cloning sgH SKAP2 into pXPR_502 Forward 5’-  CACCGGTGGGAAGGGGGCGGACTG -3’ | This paper | N/A |
| Primer for cloning sgH SKAP2 into pXPR_502 Reverse 5’-  AAACCAGTCCGCCCCCTTCCCACC -3’ | This paper | N/A |
| Primer for cloning sgI SKAP2 into pXPR_502 Forward 5’-  CACCGGCTCCTCCTCCTGCTTCC -3’ | This paper | N/A |
| Primer for cloning sgI SKAP2 into pXPR_502 Reverse 5’-  AAACGGAAGCAGGAGGAGGAGCC -3’ | This paper | N/A |
| Primer for cloning sgJ SKAP2 into pXPR_502 Forward 5’-  CACCGCTGTAAGGCGCTCCGGACGC -3’ | This paper | N/A |
| Primer for cloning sgJ SKAP2 into pXPR_502 Reverse 5’-  AAACGCGTCCGGAGCGCCTTACAGC -3’ | This paper | N/A |
| Primer for cloning sgK SKAP2 into pXPR_502 Forward 5’-  CACCGAGCGCCTTACAGCCATTTT -3’ | This paper | N/A |
| Primer for cloning sgK SKAP2 into pXPR_502 Reverse 5’-  AAACAAAATGGCTGTAAGGCGCTC -3’ | This paper | N/A |
| Primer for cloning sgL SKAP2 into pXPR_502 Forward 5’-  CACCGTATCATGGATACATTATTTTC -3’ | This paper | N/A |
| Primer for cloning sgL SKAP2 into pXPR_502 Reverse 5’-  AAACGAAAATAATGTATCCATGATAC -3’ | This paper | N/A |
| Primer for cloning sgM SKAP2 into pXPR_502 Forward 5’- CACCGACAAGAGAAAATTCCTTGT -3’ | This paper | N/A |
| Primer for cloning sgM SKAP2 into pXPR_502 Reverse 5’-  AAACACAAGGAATTTTCTCTTGTC -3’ | This paper | N/A |
| Primer for block containing rs2391362 and  rs10271396 SNPs Forward 5’- GGTCTCATTTGTGGCCCAA -3’ | This paper | N/A |
| Primer for block containing rs2391362 and  rs10271396 Reverse 5’- GCCTGCTAGTACCACATCCA-3’ | This paper | N/A |
| Primer for Sanger sequencing rs2391362 and  rs10271396 Forward 5’- TGTGTGCCTGTAGTCCTAGC-3’ | This paper | N/A |
| Primer for block containing rs3801835 and rs3801834 Forward 5’- GGAGATGGAGGGAAGAAACTAGT-3’ | This paper | N/A |
| Primer for block containing rs3801835 and rs3801834 Reverse 5’- TGGTAAAATGAAATGGCATCTGT-3’ | This paper | N/A |
| Primer for Sanger sequencing rs3801835 and rs3801834 Forward 5’-AACCGAAATTGCAAAGGGACA-3’ | This paper | N/A |
| Primer for block containing rs3801814 and rs3801815 Forward 5’- CCGAATTAGCACAGGTCACC-3’ | This paper | N/A |
| Primer for block containing rs3801814 and rs3801815 Reverse 5’- GGCAGGCTGAAAACAACGTA-3’ | This paper | N/A |
| Primer for Sanger sequencing rs3801814 and rs3801815 Forward 5’- TTCTATACGGTGCCTGGACC-3’ | This paper | N/A |
| Primer for block containing rs3801831  Forward  5’- TTGGAGACTGCAAACACCCA-3’ | This paper | N/A |
| Primer for block containing rs3801831  Reverse  5’- GCCCTGAATTGTTTGTTGGC -3’ | This paper | N/A |
| Primer for Sanger sequencing rs3801831 5’- TCTACCACCACAAAAGGAAAACC-3’ | This paper | N/A |
| Primer for *SKAP1* splice‑variant detection Forward 5’- CCAGTGCAGACCCACTATCTT | This paper | N/A |
| Primer for *SKAP1* splice‑variant detection Reverse 5’- CGTTGGAAGGACAGTTCATCT | This paper | N/A |
| Primer for Sanger sequencing of *SKAP1* splice variants Forward 5’- ATGAAGTCTTGCCAGATGAAGAG | This paper | N/A |
| *UBE2D2 expression primer* Forward 5’- GATCACAGTGGTCTCCAGCA -3’ | This paper | N/A |
| *UBE2D2 expression primer* Reverse 5’- CGAGCAATCTCAGGCACTAA -3’ | This paper | N/A |
| *SKAP2 expression primer* Forward 5’- ACCAGGGATTGTGGGATTGTAC -3’ | This paper | N/A |
| *SKAP2 expression primer* Reverse 5’-  TTCATTTCTCCTACCCACCAGC -3’ | This paper | N/A |
| *ITGA1 expression primer* Forward 5’- TGGCAGCTATAACCGAGGAAA -3’ | This paper | N/A |
| *ITGA1 expression primer* Reverse 5’-  TCAAATGAAGCTGCTGACTGG -3’ | This paper | N/A |
| *ITGB7 expression primer* Forward 5’-  GACGCCAAGATCCCATCCA -3’ | This paper | N/A |
| *ITGB7 expression primer* Reverse 5’- GACGCGGTGAAGTTCAGTTG -3’ | This paper | N/A |
| crRNA 1 for *SKAP2* knockout  5’- GAGGAAATTAGGAACCTGT -3’ | This paper | N/A |
| crRNA 2 for *SKAP2* knockout  5’- GGTTCCTAATTTCCTCAGGG -3’ | This paper | N/A |
| crRNA 1 for *SKAP1* knockout  5’- GAGGAGATCCGTTGGCTCC -3’ | This paper | N/A |
| crRNA 2 for *SKAP1* knockout  5’-GTAATCAAGCAAGGATACT -3’ | This paper | N/A |
| crRNA 3 for *SKAP1* knockout  5’- GATAATCACAGCGGGACTCT-3’ | This paper | N/A |
| Scrambled crRNA control  5’- GGTTCTTGACTACCGTAATT -3’ | Integrated DNA Technologies | N/A |
| crRNA NTC1 for CRISPRa  5’- GTAGCGAACGTGTCCGGCGT -3’ | Shifrut et al., 2018 | N/A |
| crRNA NTC2 for CRISPRa  5’- GACCGGAACGATCTCGCGTA -3’ | Shifrut et al., 2018 | N/A |
| crRNA NTC3 for CRISPRa  5’- GCTTGAGCACATACGCGAAT -3’ | Shifrut et al., 2018 | N/A |
| crRNA NTC4 for CRISPRa  5’- GTGGTAGAATAACGTATTAC -3’ | Shifrut et al., 2018 | N/A |
| crRNA NTC5 for CRISPRa  5’- GTCATACATGGATAAGGCTA -3’ | Shifrut et al., 2018 | N/A |
| crRNA NTC0 for CRISPRa  5’- GTAGGCGCGCCGCTCTCTAC -3’ | Doench et al., 2016 | N/A |
| crRNA sgA for *SKAP2* CRISPRa  5’- GAACAGCGGGGCTACGAGTC -3’ | This paper | N/A |
| crRNA sgB for *SKAP2* CRISPRa  5’- GCTACGAGTCGGGACACTGCC -3’ | This paper | N/A |
| crRNA sgC for *SKAP2* CRISPRa  5’- GCCGGGCCGGGGCTCACAACA -3’ | This paper | N/A |
| crRNA sgD for *SKAP2* CRISPRa  5’- GACAGTCCAGCTGCAGCTCGC -3’ | This paper | N/A |
| crRNA sgE for *SKAP2* CRISPRa  5’- GAGGGCCGGGGCGGGGAGAT -3’ | This paper | N/A |
| crRNA sgF for *SKAP2* CRISPRa  5’- GGGACACGAAGGGCCTGAG -3’ | This paper | N/A |
| crRNA sgG for *SKAP2* CRISPRa  5’- GTGTATTCCTCGGTTTTCC -3’ | This paper | N/A |
| crRNA sgH for *SKAP2* CRISPRa  5’- GGTGGGAAGGGGGCGGACTG -3’ | This paper | N/A |
| crRNA sgI for *SKAP2* CRISPRa  5’- GGCTCCTCCTCCTGCTTCC -3’ | This paper | N/A |
| crRNA sgJ for *SKAP2* CRISPRa  5’- GCTGTAAGGCGCTCCGGACGC -3’ | This paper | N/A |
| crRNA sgK for *SKAP2* CRISPRa  5’- GAGCGCCTTACAGCCATTTT -3’ | This paper | N/A |
| crRNA sgL for *SKAP2* CRISPRa  5’- GTATCATGGATACATTATTTTC -3’ | This paper | N/A |
| crRNA sgM for *SKAP2* CRISPRa  5’- GACAAGAGAAAATTCCTTGT -3’ | This paper | N/A |
| crRNA 1 for murine *Skap2* knockout  5’ - GGGATCCGACGTTATTCCTG - 3’ | This paper | N/A |
| crRNA 2 for murine *Skap2* knockout  5’ - AACAGGTTCCTAATTTCCTC - 3’ | This paper | N/A |
| crRNA 3 for murine *Skap2* knockout  5’ - AATATGACGATCCCTTTGCT - 3’ | This paper | N/A |
|  |  |  |
| Recombinant DNA | | |
| pScalps-Puro | Montagner et al., 2016 | Addgene plasmid # 99636 |
| pScalps Flag-SKAP2 | This paper | N/A |
| pScalps Flag-SKAP2 D241A | This paper | N/A |
| pScalps Flag-SKAP2 D71G | This paper | N/A |
| pScalps HaloTag-SKAP2 | This paper | N/A |
| pScalps HaloTag | This paper | N/A |
| pScalps 3xFlag - Nterm SKAP2 (1aa-241aa) | This paper | N/A |
| pScalps C-Term SKAP2 3xFlag (242aa-359aa) | This paper | N/A |
| pLVX EF1α – Flag-SKAP2 | This paper | N/A |
| pLVX EF1α – Flag-SKAP2 D241A | This paper | N/A |
| pLVX EF1α IRES-ZsGreen | Clontech | 631982 |
| pXPR_sgA SKAP2 | This paper | N/A |
| pXPR_sgB SKAP2 | This paper | N/A |
| pXPR_sgC SKAP2 | This paper | N/A |
| pXPR_sgD SKAP2 | This paper | N/A |
| pXPR_sgE SKAP2 | This paper | N/A |
| pXPR_sgF SKAP2 | This paper | N/A |
| pXPR_sgG SKAP2 | This paper | N/A |
| pXPR_sgH SKAP2 | This paper | N/A |
| pXPR_sgI SKAP2 | This paper | N/A |
| pXPR_sgJ SKAP2 | This paper | N/A |
| pXPR_sgK SKAP2 | This paper | N/A |
| pXPR_sgL SKAP2 | This paper | N/A |
| pXPR_sgM SKAP2 | This paper | N/A |
| pXPR_sgNTC 1 | This paper | N/A |
| pXPR_sgNTC 2 | This paper | N/A |
| pXPR_sgNTC 3 | This paper | N/A |
| pXPR_sgNTC 4 | This paper | N/A |
| pXPR_sgNTC 5 | This paper | N/A |
| pXPR_sgNTC 0 | This paper | N/A |
| pZR112_Lenti-SFFV-mCherry-2A-dCas9-VP64 | Schmidt et al., 2022 | Addgene plasmid # 180263 |
| pLenti-SFFV-ZsGreen-2A-dCas9-VP64 | This paper | N/A |
| pXPR_502 | Sanson et al., 2018 | Addgene plasmid # 96923 |
| pMD2.G | http://n2t.net/addgene:12259 | Addgene plasmid # 12259 |
| psPAX2 | http://n2t.net/addgene:12260 | Addgene plasmid # 12260 |
|  |  |  |
| Software and algorithms | | |
| Fiji ImageJ v1.53h | Schindelin et al., 2012 | https://imagej.net/software/fiji/ |
| Image Lab software | Bio-Rad Laboratories | Version 6.1 |
| MetaXpress PowerCore | Molecular Device Software |  |
| FlowJo v10.6.0 | FlowJo, LLC | https://www.flowjo.com/ |
| GraphPad Prism v9 | GraphPad Software | https://www.graphpad.com/ |
| IGV 2.16.0 | Robinson et al., 2011 | https://software.broadinstitute.org/software/igv/2.16.x |
| Cascleave | Wang et al., 2014 |  |
| QuikChange Primer Design | Agilent | http://www.genomics.agilent.com/primerDesignProgram.jsp |
| Others | | |
| EVOS M5000 Imaging System | Thermo Fisher Scientific | Catalog # AMF5000 |
| Molecular Devices ImageXpress Micro 4 | Molecular Device |  |
| QuantStudio 3 Real-Time PCR System | Thermo Fisher Scientific | Catalog # A28136 |
| ABI 7900HT Fast Real-Time PCR system | Applied Biosystem |  |
| Fusion FX7 EDGE Imaging System | Witec | Catalog # 1511 0200 1 |
| ChemiDoc XRS+ System | Bio-Rad Laboratories |  |
| BD FACSCelesta Cell Analyzer | BD Biosciences |  |
| BD FACSymphony A5 Cell Analyzer | BD Biosciences |  |
| BD SORP FACSymphony S6 | BD Biosciences |  |
| Neon Transfection System | Thermo Fisher Scientific | Catalog # MPK5000 |
| Qubit 3 Fluorometer | Thermo Fisher Scientific | Catalog # Q33216 |
| GloMax Discover | Promega | Catalog # GM3000 |
| DynaMag-2 Magnet | Thermo Fisher Scientific | Catalog # 12321D |
| EASY-nLC 1200 HPLC System | Thermo Fisher Scientific | Catalog # LC140 |
| Q Exactive HF mass spectrometer | Thermo Fisher Scientific | Catalog # 1372090 |
| Corning 6.5 mm Transwell with 8.0 µm Pore Polycarbonate Membrane Insert | Corning | Catalog # 3422 |
| Agilent TapeStation system | Agilent |  |
| Illumina NovaSeq 6000 platform | Illumina |  |
| Amaxa 4D X Nucleofection System | Lonza Bioscience | Catalog # AAF-1003X |
| µ-Slide Chemotaxis 3D | IBIDI | Catalog # 80326 |
| HTS Transwell-96 Permeable Support with 3.0 μm pore polycarbonate membrane | Corning | Catalog # 3386 |
